# Phylogenetic relationship and domain organisation of SET domain proteins of Archaeplastida

**DOI:** 10.1186/s12870-017-1177-1

**Published:** 2017-12-11

**Authors:** Supriya Sarma, Mukesh Lodha

**Affiliations:** Centre for Cellular and Molecular Biology (CSIR), Uppal Road, Habsiguda, Hyderabad, 500007 India

**Keywords:** Archaeplastida, Histone modifications, Epigenetics, SET domain, Polycomb, Phylogenetic analysis, Evolution

## Abstract

**Background:**

SET is a conserved protein domain with methyltransferase activity. Several genome and transcriptome data in plant lineage (Archaeplastida) are available but status of SET domain proteins in most of the plant lineage is not comprehensively analysed.

**Results:**

In this study phylogeny and domain organisation of 506 computationally identified SET domain proteins from 16 members of plant lineage (Archaeplastida) are presented. SET domain proteins of rice and Arabidopsis are used as references. This analysis revealed conserved as well as unique features of SET domain proteins in Archaeplastida. SET domain proteins of plant lineage can be categorised into five classes- E(z), Ash, Trx, Su(var) and Orphan. Orphan class of SET proteins contain unique domains predominantly in early Archaeplastida. Contrary to previous study, this study shows first appearance of several domains like SRA on SET domain proteins in chlorophyta instead of bryophyta.

**Conclusion:**

The present study is a framework to experimentally characterize SET domain proteins in plant lineage.

**Electronic supplementary material:**

The online version of this article (10.1186/s12870-017-1177-1) contains supplementary material, which is available to authorized users.

## Background

Epigenetic cellular memory is heritable during mitosis and/meiosis but is not encoded in the genetic material [1, 2]. Histone post-translational modifications, DNA methylation, and non-coding RNAs and chromatin remodelling are the major components of epigenetic inheritance [3]. Numerous histone modifications like acetylation, methylation and phosphorylation occur mainly in the N-terminal tails of the histones [2, 4–6]. These histone modifications are associated with transcriptional repression or activation, DNA repair and DNA recombination [7]. Among various histone modifications, lysine methylation is rigorously studied in plant, fungi, and animal models [8, 9]. Lysine residues can be mono, di and tri-methylated [8]. Lysine methylation is catalyzed by 130–150 amino acid protein domain. The name SET is derived from Drosophila proteins, *Su*(*var*)*3–9* [10], *Enhancer of zeste, E*(*z*) [11], and *Trithorax* (*trx*) [12] having this domain. The only exception is the H3K79 methylation mark deposited by DOT1 methyltransferase that does not contain SET domain [13].

Based on SET domain sequence similarity to Drosophila homologues, plant SET domain proteins are classified into 4 main classes [14], Enhancer of Zeste, E(z) homologs; Absent, Small, or Homeotic disks (Ash) homologs and related proteins; Trithorax (Trx) homologs and related proteins; and Suppressor of Variegation, Su(var) homologs and related proteins [14–16]. Su(var) and E(z) group proteins are mainly involved in transcription repression, whereas Trx and Ash group are mainly involved in transcriptional de-repression [16, 17]. A separate category is later added to include proteins with an interrupted SET domain, lowly conserved SET domain and proteins with TPR and Rubisco domains [18–20]. This category of proteins are not well characterized for their biochemical activity.

Here we have taken a computational approach to analyse SET domain sequences of 16 different taxa of Archaeplastida. 506 SET proteins are identified that are classified into five classes. Our work will serve as a guideline for functional and biochemical characterization of SET proteins of plant lineage.

## Methods

### Identification of SET domain proteins in Archaeplastida

To uncover the SET proteins in Archaeplastida, the conserved SET domain amino acid sequences from *Arabidopsis thaliana* (At), *Oryza sativa* (Os), *Picea abies* (Pa), *Selaginella meollendorffii* (Sm), *Physcomitrella patens* (Pp), *Micromonas pusila* (Mpu), *Micromonas RCC299* (Mr), *Ostreococcus tauri* (Ot), *Ostreococcus lucimerinus* (Ol), *Chlorella vulgaris* (Cv), *Chlamydomonas reinhardtii* (Cr) and *Volvox carteri* (Vc) are retrieved by BLAST search using SET domain (PF00856) as query from PFAM database Version 30.0 (http://pfam.xfam.org/family/PF00856) [21]. The SET domain protein sequences are also retrieved from Uniprot (http://www.uniprot.org/) and Phytozome v12.1 (https://phytozome.jgi.doe.gov/pz/portal.html) that are not available in PFAM. To better understand the evolutionary history and functionality of SET domain-containing proteins in plant lineages, we have use assembled transcriptome data from, *Nitella mirabilis* (Nm) (https://www.ncbi.nlm.nih.gov/bioproject/PRJNA158153 158153)*, Klebsormidium flaccidum* (Kf) (https://www.ncbi.nlm.nih.gov/bioproject/ PRJNA 51159). Cyanophora *paradoxa* (http://cyanophora.rutgers.edu/cyanophora/) and predicted proteome data for *Marchantia polymorpha* (Mp) (https://www.ncbi.nlm.nih.gov/bioproject/PRJNA218052) and Gymnosperm, *Picea abies* (Pa) (http://marchantia.info/tom/blast/blast.html tom/blast/blast.html). In in silico proteome datasets, if two or more overlapping protein sequences arose from same genetic locus, we have selected the longest sequence. Transcript sequences obtained from NCBI website are transdecoded into predicted proteins using Transdecoder (https://transdecoder.github.io/) that gave the longest open reading frame. Local Blastp (ftp://ftp.ncbi.nlm.nih.gov/blast/executables/blast+/LATEST/. nih.gov/blast/executables/blast+/LATEST/) is carried out with these predicted proteins against 130–150 length SET domain sequences from *Arabidopsis thaliana* to filter the SET domain containing sequences. Thereafter, redundant SET domain amino acid sequences are removed from all the SET domain sequences obtained from the online search. The *Arabidopsis thaliana* and *Oryza sativa* SET domains protein sequences are used as queries because it is a representative of flowering plants and possess relatively complete annotated protein information.

### Domain architecture

Presence or absence of specific domain and its organisation might provide an intimation to the functional divergence of the different protein groups. Additionally, it is conventional that proteins sharing identical domain may share close evolutionary relationship and may be functionally related. Predicted proteins from PFAM and local Blast were searched for the signature SET domain and their associated domain using NCBI Batch CD search tool (http://www.ncbi.nlm.nih.gov/Structure/bwrpsb/bwrpsb.cgi/) and NCBI-CD Hit (https://ww w.ncbi.nlm.nih.gov/Structure/cdd/wrpsb.cgi).

### Phylogenetic analysis

For phylogenetic tree construction, 251 SET domain sequences retrieved from the E(z), Ash, Trx and Su(var) group are included. SET domain containing proteins from the Orphan, SETD, and TPR are excluded due to low sequence similarity with E(z), Ash, Trx and Su(var). Multiple sequence alignments of SET domain sequences are performed using the ClustalW program. Evolutionary analyses are conducted in MEGA7.0 program [22]. The evolutionary history is inferred by using the Maximum Likelihood method based on the JTT matrix based model with a bootstrap test of 1000 replicates for internal branch reliability and use all sites option as Gaps/Missing data treatment. The tree with the highest log likelihood (−39,059.190) is shown. Initial tree(s) for the heuristic search are obtained automatically by applying Neighbour-Joining and BioNJ algorithms to a matrix of pairwise distances estimated using a JTT model, and then selecting the topology with superior log likelihood value.

### Multiple alignments of SET domain sequences

Multiple sequence alignment is performed for the unannotated conserved SET domain sequences of plant lineages with reference *Arabidopsis* and rice SET domain sequences using multalin program (http://multalin.toulouse.inra.fr/multalin/) with the default parameters. SET domain sequences sharing significant homology with *Arabidopsis* and rice. SET domain sequences are categorized to the corresponding group of the SET domain. Classification is also interpreted based on neighbouring domain organization.

### Nomenclature and classification of SET domain proteins

SET domain proteins are named according to the nomenclature of annotated plant and animal SET domain histone methyltransferases with generic capitalized letter followed by species name in the lower case using the common specific protein name. SET domain proteins are named according to the name of the closest *Arabidopsis thaliana* and *Oryza sativa* homolog. If two or more proteins were equally close to a single *Arabidopsis thaliana* and *Oryza sativa* SET domain containing proteins, then the same name is used followed by the letters “a”, “b”, etc. The following nomenclature is adopted for classes and subclasses of the SET domain-containing proteins: For e.g. protein type, II-2C would belong to Class II, subclass 2 and subdivision of subclass C.

## Results

### Identification and classification of SET domain proteins of Archaeplastida into five classes

**Fig. 1:**
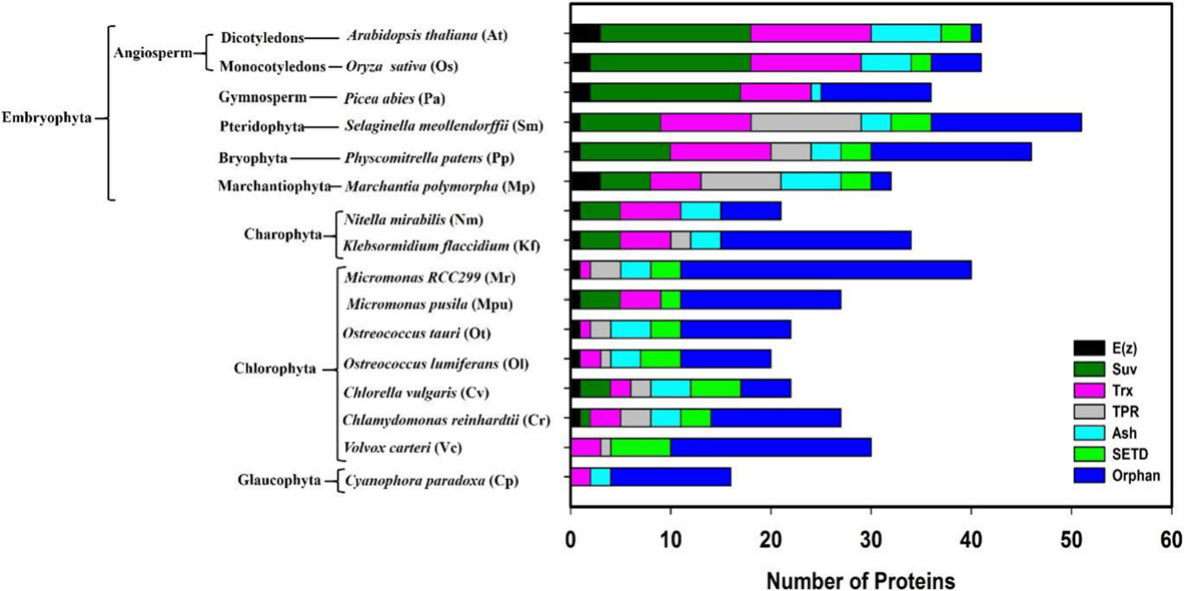
Graphical representation of SET domain containing proteins in 16 representative Archaeplastida. SET domain proteins can be grouped into 5 classes. Class I, E(z); Class II, Ash; Class III, Trx; Class IV, Su(var); Class V includes Orphan, SETD, and TPR. Each class is color coded

To get an insight into the SET domain proteins from plant lineage and their probable functional relationship, we performed a homology search from 16 Archaeplastida against SET domain sequences retrieved from PFAM and NCBI transcriptome assembly. A total of 506 candidate SET domain containing protein sequences are identified (Fig. 1, Additional file 1: Table S1 and Additional file 2: Table S2). This SET domain homology search identified a range of 25–50 SET domain containing proteins in each plant species. SET proteins identified in this study range from 200 to 3500 amino acid in length. The numbers of the SET domain-containing proteins show a non-linear increment with the evolution of compelxification (Fig. 1).

SET domain proteins can be grouped into 5 classes. Class I as E(z), Class II as Ash, Class III as Trx and Class IV as Su(var) and Class V. Class II, III and IV each has also a subclass called Orphan that has SET domain with significantly low homology to the conserved SET domain or absence of signature domains of the respective class. Class V includes Orphan, SETD, and TPR with low sequence similarity in the SET domains to other 4 groups. SETD protein family has a characteristic Rubisco domain. TPR has tetratricopeptide domain while Orphan family have either an interrupted SET domain or have SET domain with few associated domains (Fig. 1). Classification presented here is uniform with already annotated histone methyltransferases from other plant species with few modifications. Few of the genomes considered in the present study are in draft stage, hence some proteins in our analysis may be missing or may have errors in the predicted protein structure.

The numbers of the SET domain containing proteins in unicellular glaucophyta species, *Cyanophora paradoxa*, (16) are about 3-fold lower compared to pteridophyta species, *Selaginella meollendorffii* (51). Nonetheless, small number of SET domain proteins detected in *Cyanophora paradoxa* may also be due to missing proteins in the currently available proteome. E(z) group of proteins is not detected in, glaucophyta and chlorophyta sp. *Volvox carteri* though they are present in another chlorophyta species, *Chlamydomonas reinhardtii*. Ash and Trx group of proteins are identified as early as in glaucophyta and there is a nonlinear increase in the number of Ash group with the increase in complexification of species. Among the four main subfamilies, Ash proteins have maintained nearly stable numbers among plant species. However, a noteworthy and surprising feature is the absence of Ash group of proteins in *Micromonas pusila* (Fig. 1). Trx proteins show a sudden doubling in number in charophyta, marchantiophyta, bryophyta, and pteridophyta as compared to chlorophyta, glaucophyta. Su(var) proteins are absent in glaucophyta and chlorophyta species, *volvox carteri*, *Ostreococcus* sp. and *Micromonas RCC*299 and then there is a gradual increase in the number among, marchantiophyta, bryophyta, and pteridophyta. Tetratricopeptide repeat (TPR) group of proteins is first identified in chlorophyta, *Chlamydomonas reinhardtii* with their highest abundance in pteridophytes. SETD proteins are identified in chlorophyta but there is no proportional increase in number in various species of plant lineage. The absence of SETD in glaucophyta and charophyta remains controversial. Unlikely, Orphan proteins are identified since the emergence of glaucophyta. Orphan protein shows highest abundance in *Micromonas RCC299*, while *Marchantia polymorpha* possess a minimum number of Orphan family proteins. Sequence comparisons are performed among SET domain containing proteins from other Archeaplastida members and with reference species, *Arabidopsis thaliana* and *Oryza sativa*. Increased numbers and diversification of encoded SET domain proteins in embryophyta perhaps reflect increased diversity and complexification of multicellular programs. Characterization of these domains will be useful in understanding functions of SET domain proteins.

### Phylogenetic analysis

**Fig. 2:**
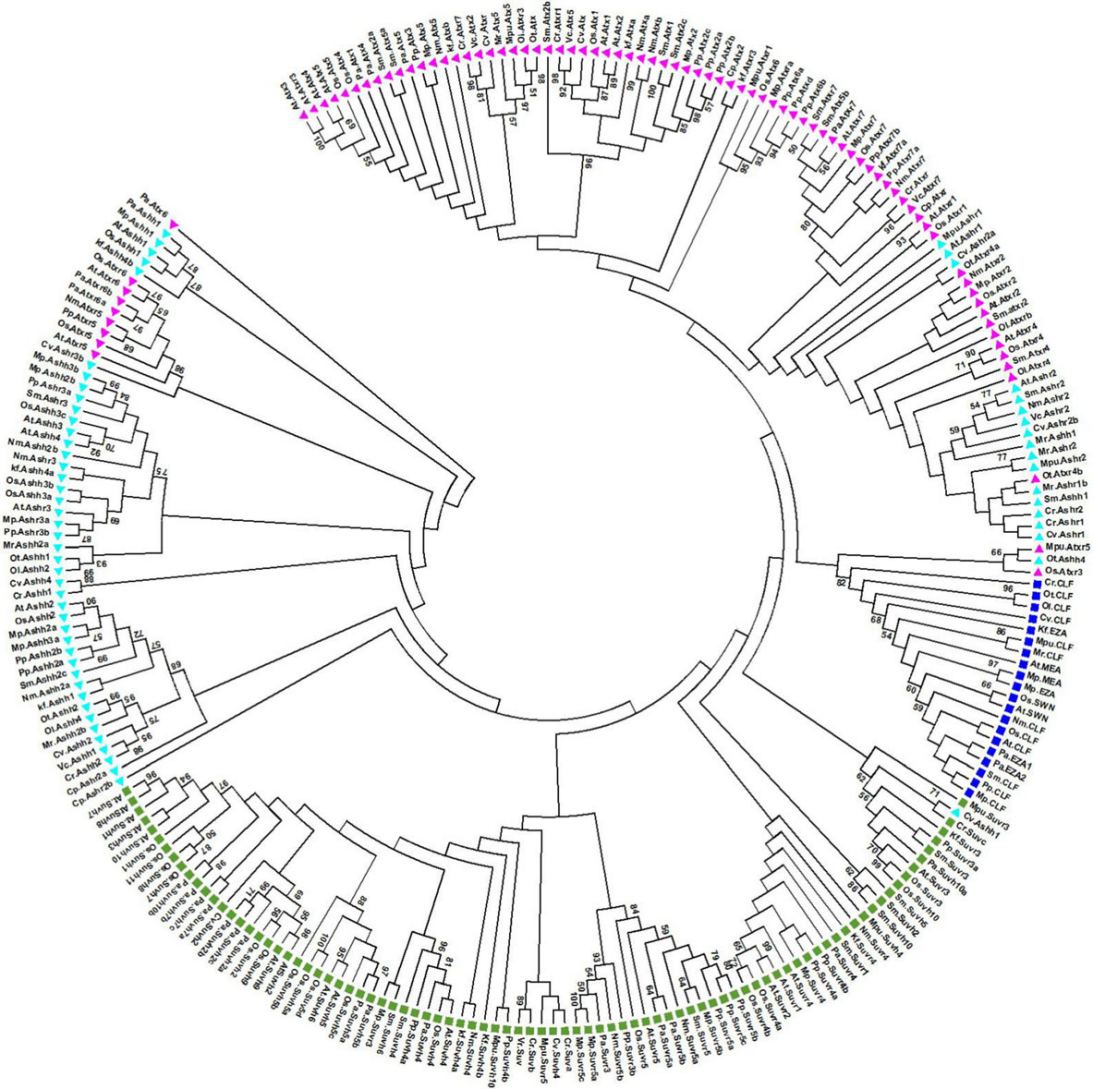
Phylogenetic tree of SET-domain proteins in Archaeplastida. The SET domain sequences of 251 proteins from 16 species are aligned using ClustalW. Phylogenetic analysis is performed using MEGA 7.0. SET domain proteins of Archaeplastida are divided into four distinct groups: E(z), Ash, Trx and Su(var)3–9 proteins. Branch nodes are colored distinctly for separate clade mentioned in the accompanying legends. The dark blue rectangle indicates E (z) group, Cyan blue triangle indicates Ash group, the Pink triangle indicates Trx group while green rectangle indicates Suv group. The evolutionary history is inferred by using the Maximum Likelihood method based on the JTT matrix-based model. The tree with the highest log likelihood (−39,445.1504) is shown. Initial trees for the heuristic search are obtained automatically by applying Neighbor-Join and BioNJ algorithms to a matrix of pairwise distances estimated using a JTT model, and then selecting the topology with superior log likelihood value

Two hundred and fifty one SET domain proteins belonging to E(z), Ash, Trx, and Su(var) are considered for phylogenetic clustering. Remaining SET domain candidate proteins from Orphan, SETD, and TPR are excluded from phylogenetic analysis due to low sequence similarity. The multiple sequence alignment of the 251 SET domain protein sequences are provided in Additional file 3: Figure S1. Based on the presence and absence of group-specific architectural motifs and sequence alignment, these 251 SET domain protein sequences are classified into E(z), Ash, Trx, and Su(var). A noteworthy point is that many Ash and Trx related proteins diverge from their respective branch (Fig. 2).

### Class I-enhancer of Zeste, E(z)

**Fig. 3:**
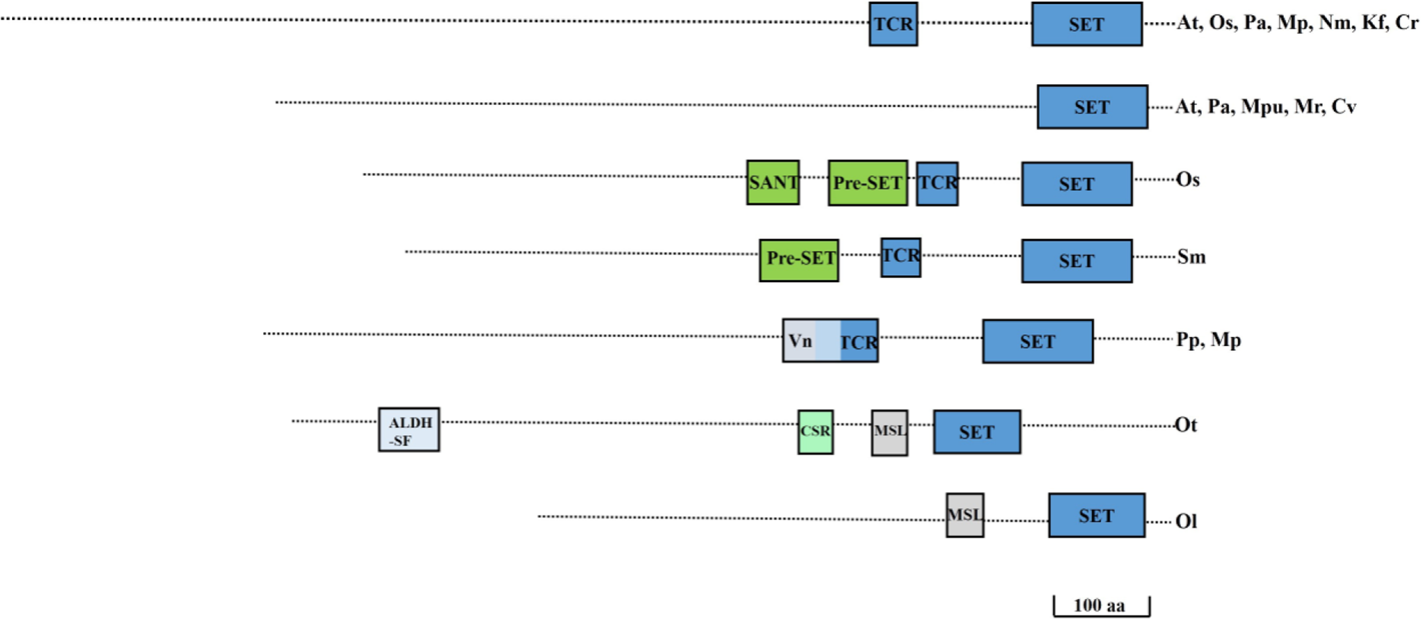
Domain architecture of the E(z) family. Schematic diagrams show the domain organization of E(z) proteins. For the E(z) family, seven major representative subgroups differing by the positioning of the SET domain and type of associated domains are shown. The corresponding name of the species sharing the specific domain architecture are placed on the right side. The divergent domains are indicated by different color in the figure with the names on top. SANT: Swi3, Ada2, N-Cor, and TFIIIB; TCR:Tesmin/TSO1; Vn: Vanadium binding protein; ALDH-SF: aldehyde dehydrogenase superfamily; CSR: Cysteine/serine-rich nuclear protein; MSL: Male-specific lethal. Domains are not drawn to scale. Scale bars indicate 100 amino acids. (At- *Arabidopsis thaliana*; Os-*Oryza sativa*; Pa-*Picea abies*; Sm-*Selaginella meollendorffii;* Pp*-Physcomitrella patens;* Mp*-Marchantia polymorpha;* Nm*-Nitella mirabilis;* Kf-*Klebsormidium flaccidium;* Mr.*-Micromonas RCC299;* Mpu*-Micromonas pusila;* Ot*-Ostreococcus tauri; Ol-Ostreococcus lumiferans;* Cv*-Chlorella vulgaris; Cr-Chlamydomonas reinhardtii;* Vc*-Volvox carteri;* Cp*-Cyanophora paradoxa*)

E(z) is the catalytic component of Polycomb Repressive complex2 characterized in few members of plant lineage, catalysing the di- and tri-methylation of H3K27 [23]. E(z) class of proteins are named as Class I SET domain proteins in our study. Based on presence of other domains in combination of SET domain, seven variations of E(z) proteins is noted. Unlike other classes, this class of proteins appears to have strict and less diversified domain combinations. (Fig. 3). A PreSET domain is present in moncot, rice and pteridophyte, *Selaginella*. A vanadium binding domain is present in *Physcomitrella patens* and *Marchantia polymorpha*. Cysteine/serine-rich nuclear protein, (CSR) and Male-specific lethal (MSL) domains are *Ostreococcus specific* ***.*** *Cyanophora* and *Volvox* lack this class of protein. Interestingly most members in plant lineage contain only one E(z) homolog whereas *Marchantia* and *Arabidopsis* contain three and *Picea* contains two homologs.

### Class II- absent, small, or Homeotic disks (ash) and related proteins

**Fig. 4:**
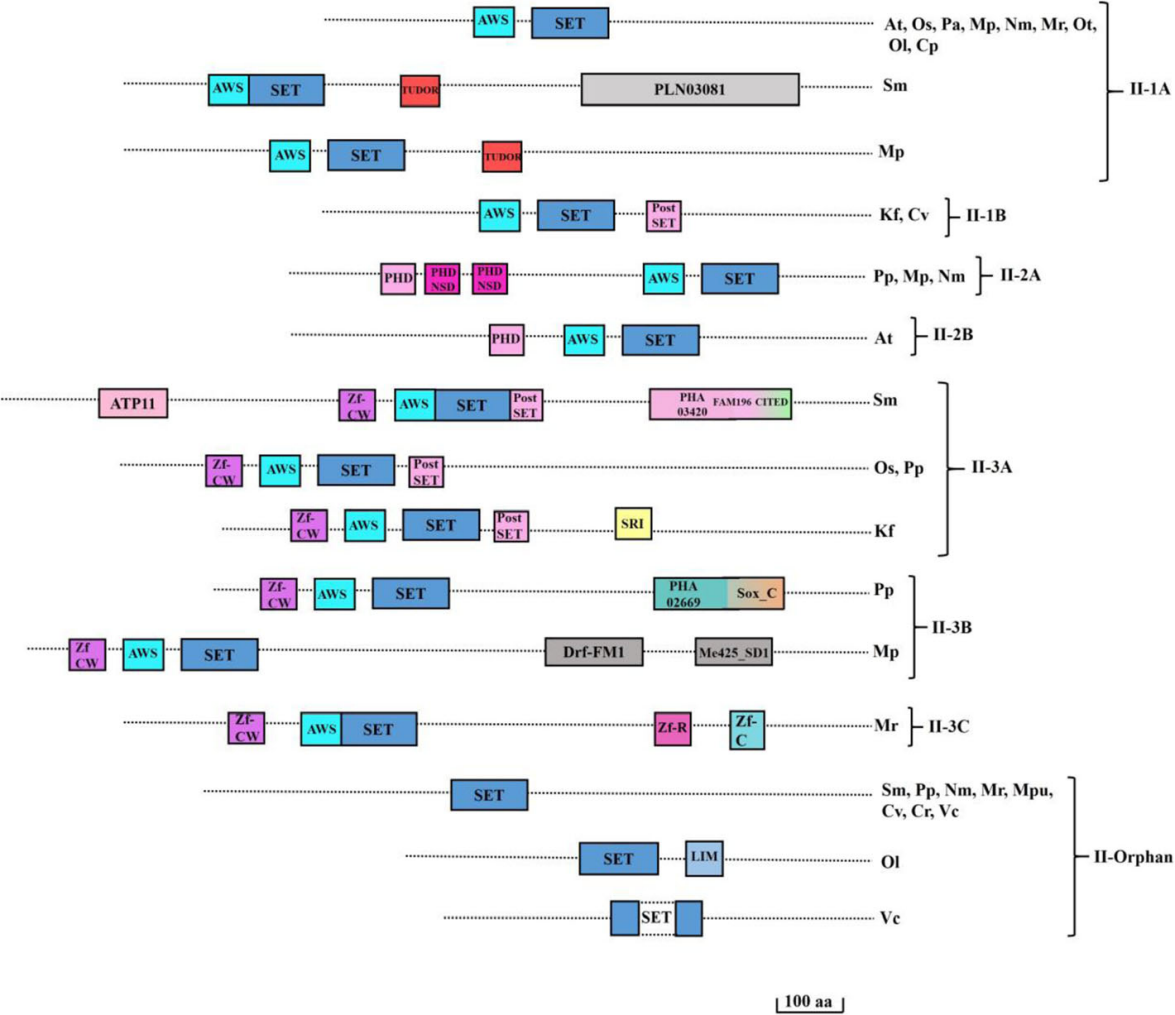
Domain architecture of the Ash family. Schematic representation of Ash family proteins. Three major representative subgroups differing by the positioning of the SET domain and type of associated domain domains are shown along with an orphan group. These groups are further sub divided. The right side of the protein indicates the abbreviated form of the species sharing a particular protein architecture. Associated domains are indicated by different colored boxes with the names. AWS: Associated with SET; PHD: Plant homeodomain; PHD_NSD: Plant homeodomain found in nuclear receptor-binding SET domain-containing proteins; Zf-MYND: Myeloid nervy, deaf; Zf_C: Zinc binding motif composed of cysteine motif; TPR: Tetratricopeptide Repeat; Zf-CW: Zinc finger domain with conserved cysteine and tryptophan residues; TUDOR: Royal family protein; PLN03081: pentatricopeptide (PPR) repeat containing protein; PKC: Protein kinase catalytic domain; TNG2: T-cell leukemia neighbouring genes; ATP_11: Adenosine tri phosphate 11; PHA_03420: E4 protein; PHA02669: Hypothetical protein; CITED: CBP/p300-interacting transactivator with ED-rich tail, FAM196: Family of unknown function; SRI: Set2 Rbp1 interacting; Zf_R: Zn-finger in Ran binding protein and others; SoxC: Sry-related HMG box; Drf_FM1: Diaphanous related formin homology region1; Me425_SD1: Mediator complex 25 iynapsin 1; LIM: Lin11, Isl-1 & Mec-3. Domains are not drawn to scale. Scale bars indicate 100 amino acids. (At- *Arabidopsis thaliana*; Os-*Oryza sativa*; Pa-*Picea abies*; Sm-*Selaginella meollendorffii;* Pp*-Physcomitrella patens;* Mp*-Marchantia polymorpha;* Nm*-Nitella mirabilis;* Kf-*Klebsormidium flaccidium;* Mr.*-Micromonas RCC299;* Mpu*-Micromonas pusila;* Ot*-Ostreococcus tauri; Ol-Ostreococcus lumiferans;* Cv*-Chlorella vulgaris; Cr-Chlamydomonas reinhardtii;* Vc*-Volvox carteri;* Cp*-Cyanophora paradoxa*)

Ash class of proteins have diverse domain combinations. Based on domain combinations a novel classification of Ash proteins is presented here. Ash proteins are classified into 4 classes, Class II-1 to Class II-3 and II-Orphan (Table 1). Class II-1 and II-2 are divided into 2 subclasses whereas Class II-3 is subdivided into 3 subclasses. Except orphan class, all proteins have AWS (Associated with SET domain) suggesting its indispensible role in functions of Ash class of proteins (Fig. 4). Class II-1A has AWS domain followed by SET domain, whereas Class II-1B has PostSET domain. A chlorophyta member, *Chlorella vulgaris* shows the initial presence of PostSET domain in Ash family (Additional file 4: Figure S2) Class II-2 has Plant Homeo Domain (PHD) followed by AWS and SET domain. Very interestingly, *Physcomitrella patens*, *Marchantia polymorpha*, and *Nitella mirabilis* have three PHD domains in tandem. Most of the class II-3 proteins have zinc fingers. This domain is introduced in charophyta species, *Micromonas RCC299* (Additional file 4: Figure S2*)*. This class of proteins are likely to methylate multiple amino acids at different positions in histones.

**Table 1:** Classification of Class II-Ash proteins

Class		Type	Domain Architecture	Species																Extra Domains
				At	Os	Pa	Sm	Pp	Mp	Nm	Kf	Mr	Mpu	Ot	Ol	Cv	Cr	Vc	Cp	
II	II-1	1A	AWS-SET	+	+	+	+	–	+	+	–	+	–	+	+	–	–	–	+	TUDOR, PLN03081,
		1B	AWS-SET-PostSET	–	–	–	–	–	–	–	+	–	–	–	–	+	–	–	–	
	II-2	2A	PHD-PHD-PHD-AWS-SET	–	–	–	–	+	+	+	–	–	–	–	–	–	–	–	–	
		2B	PHD-AWS-SET	+	–	–	–	–	–	–	–	–	–	–	–	–	–	–	–	
	II-3	3A	ZnF_C2H2-AWS-SET-PostSET	+	+	-	+	+	+	–	+	–	–	–	–	–	–	–	–	ATP11, PHA03420, CITED, FAM196, SRI.
		3B	ZnF_C2H2-AWS-SET	–	–	–	–	+	+	–	–	–	–	–	–	–	–	–	–	PHA, SOX, Drf_ FM1, Me425_SD1
		3C	ZnF_C2H2-AWS-SET- ZnF_C2H2 ZnF_C2H2	–	–	–	–	–	–	–	–	+	–	–	–	–	–	–	–	
	II-Orphan		SET	+	–	–	–	+	+	–	+	–	+	+	–	+	+	+	+	LIM

Ash proteins were classified into 4 classes, Class II-1 to Class II-3 and Class II-Orphan which were further divided into subclasses. The detailed domain arrangement with additional domains are shown. (At- *Arabidopsis thaliana*; Os-*Oryza sativa*; Pa-*Picea abies*; Sm-*Selaginella meollendorffii;* Pp*-Physcomitrella patens;* Mp*-Marchantia polymorpha;* Nm*-Nitella mirabilis;* Kf-*Klebsormidium flaccidium;* Mr.*-Micromonas RCC299;* Mpu*-Micromonas pusila;* Ot*-Ostreococcus tauri; Ol-Ostreococcus lumiferans;* Cv*-Chlorella vulgaris; Cr-Chlamydomonas reinhardtii;* Vc*-Volvox carteri;* Cp*-Cyanophora paradoxa.* Plus sign (+) and minus sign (−) indicate presence and absence of an above mentioned domain architecture

### Class III-Trithorax Homologs and related proteins (Trx)

**Fig. 5:**
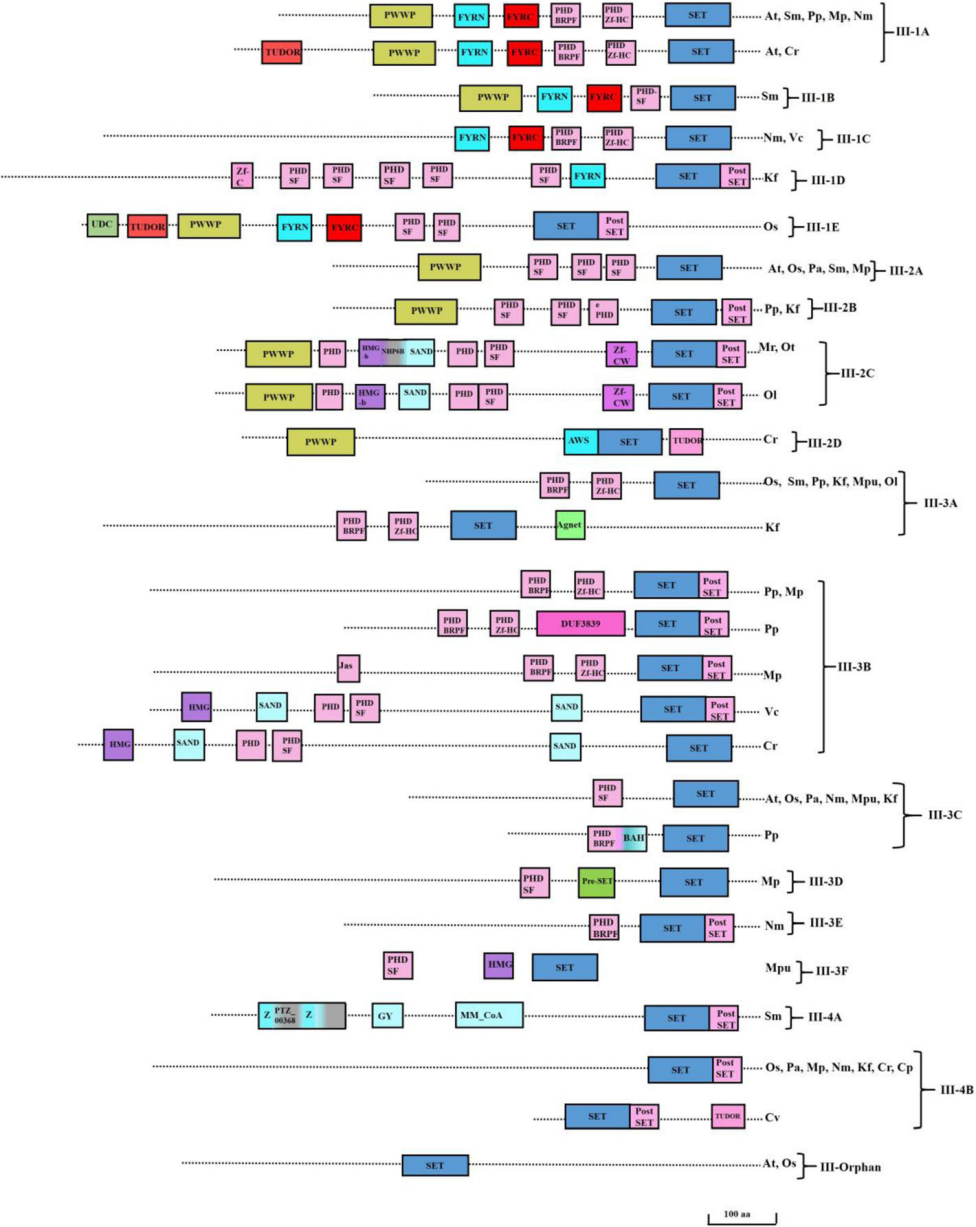
Domain architecture of the Trx family. Representative domain organization of the Trx proteins. Trx proteins are divided into four groups with subgrouping of each group. Right side panel indicates the abbreviated form of the species sharing the domain arrangement. PHD1: Plant homeodomain1; PHD_SF: Plant homeodomain superfamily; Ephd: extended plant homeodomain; FYRN_C: phenylalanine/tyrosine-rich domain in N-terminal and C-terminal region; Z: zinc knuckle is a zinc binding motif composed of cysteine residue motifs found mostly from retroviral gag proteins; HMG-b: High mobility group box; Bro-Bromodomain; AP2: Apetala2; TUDOR: Royal family protein; NHP6B: Chromatin-associated proteins containing the HMG domain; Agnet: Royal family domain; DUF_3839: Domain of unknown function; Jas: Jasmonate motif; BAH: Bromo-adjacent homology; MBT: Malignant brain tumor; PTZ_00368: hypothetical protein; GYF: GYF domain: contains conserved Gly-Tyr-Phe; MM_CoA: Methylmalonyl CO enzyme A; SAND: Sp100, AIRE-1, NucP41/75, DEAF-1 domain. Domains are not drawn to scale. Scale bars indicate 100 amino acids. (At- *Arabidopsis thaliana*; Os-*Oryza sativa*; Pa-*Picea abies*; Sm-*Selaginella meollendorffii;* Pp*-Physcomitrella patens;* Mp*-Marchantia polymorpha;* Nm*-Nitella mirabilis;* Kf-*Klebsormidium flaccidium;* Mr.*-Micromonas RCC299;* Mpu*-Micromonas pusila;* Ot*-Ostreococcus tauri; Ol-Ostreococcus lumiferans;* Cv*-Chlorella vulgaris; Cr-Chlamydomonas reinhardtii;* Vc*-Volvox carteri;* Cp*-Cyanophora paradoxa*)

Based on earlier classification, Trx proteins are designated as Class III and subdivided into four subclasses, Class III-1 to Class III-4 [24]. Each subclass is further divided into subclasses-III-1A to E, III-2A to D, III-3A to F and III-4A, B and orphan (Table 2). In addition to the SET domain, Class III proteins bear several domains like Proline-Tryptophan-Tryptophan-Proline residue domain (PWWP), Phenylalanine-Tyrosine residue domain (FYR), PHD and PostSET domains (Fig. 5). PWWP, PHD, FYR domains are detected as early as in chlorophyta species, *Volvox carteri* (Additional file 5: Figure S3). Class III-1 in Trx uniquely has FYR and PWWP domains. The FYR domain is composed of an FYR N-terminal portion and FYR C-terminal portion that usually occurs near each other. Few species like *Chlamydomonas reinhardtii*, *Arabidopsis thaliana* and *Oryza sativa* have Royal Family domain (TUDOR) in this class of proteins. Variations in the number of PHD domain were noted among the subclass of Class III-1. The III-2 group usually contains one PWWP domain, two to three PHD domain, and one PostSET domain without FYR domain (Fig. 5). Class III-2A lacks the PostSET domain. A unique feature of Class III-2C is the presence of Zinc Finger (ZnF), Sp100, AIRE-1, NucP41/75, DEAF-1 (SAND) and High mobility group box (HMGb) domain along with canonical PWWP, PHD, SET and PostSET domain. Another striking feature is the presence of both AWS and PWWP domain (signature domain of Class II and Class III respectively) in a single protein type in Class III-2D. Class III-3 lacks PWWP domain and is characterized by the presence of PHD and SET domains. Subclass III-3B and III-3E have an additional PostSET domain. Class III-3D possess a single PreSET domain whereas Class III-3F possess HMG domain. Class III-3 possess additional Apetala 2 (AP2), Agnet (Royal family domain), Bromo-adjacent homology (BAH) and Malignant brain tumor (MBT) domains in addition to the canonical domains. Glaucophyta lacks all other domains except SET and PostSET. Class III-4A contains one additional Glycine-Tyrosine-Phenylalanine residue (GYF), ZnF_C2H2 and HMG domains. These domains are reported for RNA binding or single-stranded DNA binding in eukaryotic proteins [25]. Class III-4B usually lack additional domains except for a SET and a PostSET domain without an additional domain.

**Table 2:** Classification of Class III-Trx proteins

Class		Type	Domain Architecture	Species																Extra Domains
				At	Os	Pa	Sm	Pp	Mp	Nm	Kf	Mr	Mpu	Ot	Ol	Cv	Cr	Vc	Cp	
III	III-1	1A	PWWP-FYR-PHD-PHD-SET	**+**	–	–	**+**	+	+	+	–	–	–	–	–	–	+	–	–	TUDOR
		1B	PWWP-FYR-PHD-SET	–	–	–	+	–	–	–	–	–	–	–	–	+	–	–	–	
		1C	FYR-PHD-PHD-SET	–	–	–	–	–	–	+	–	–	–	–	–	–	–	+	–	
		1D	ZnF_C2H2-PHD-PHD-PHD-PHD-PHD-FYR-SET-PostSET	–	–	–	–	–	–	–	+	–	–	–	–	–	–	–	–	
		1E	PWWP-FYR-PHD-PHD-SET-PostSET	–	+	–	–	–	–	–	–	–	–	–	–	–	–	–	–	TUDOR, UDS
	III-2	2A	PWWP-PHD-PHD-PHD-SET	+	+	+	+	–	+	–	–	–	–	–	–	–	–	–	–	COG5141
		2B	PWWP-PHD-PHD-PHD-SET-PostSET	–	–	–	–	+	–	–	+	–	–	–	–	–	–	–	–	
		2C	PWWP-PHD-HMGb-SAND-PHD-PHD-ZnF_C2H2-SET-PostSET	–	–	–	–	–	–	–	–	+	–	+	+	–	–	–	–	NHPGB
		2D	PWWP-AWS-SET	–	–	–	–	–	–	–	–	–	–	–	–	–	+	–	–	TUDOR
	II-3	3A	PHD-PHD-SET	–	+	–	+	+	–	–	+	–	+	–	+	–	–	–	–	Agnet
		3B	PHD-PHD-SET-PostSET	–	–	–	–	+	+	–	–	–	–	–	–	–	+	+	–	DUF3839, Jas
		3C	PHD-SET	+	+	+	–	+	–	+	–	–	+	–	–	–	–	–	–	BAH
		3D	PHD-PreSET-SET	–	–	–	–	–	+	–	–	–	–	–	–	–	–	–	–	
		3E	PHD-SET-PostSET	–	–	–	–	–	–	+	–	–	–	–	–	–	–	–	–	
		3F	PHD-HMG-SET	–	–	–	–	–	–	–	–	–	+	–	–	–	–	–	–	
	III-4	4A	ZnF_C2H2-GYF-HMGCOA-SET- PostSET	–	–	–	+	–	–	–	–	–	–	–	–	–	–	–	–	PTZ, GY, MM_COA
		4B	SET- PostSET	–	+	+	–	–	+	+	+	–	–	–	–	+	+	–	+	TUDOR
	III-Orphan		SET	+	+	–	–	–	–	–	–	–	–	–	–	–	–	–	–	

Trx proteins were classified into 4 classes, Class III-1 to Class III-4 which were further divided into subclasses. (At- *Arabidopsis thaliana*; Os-*Oryza sativa*; Pa-*Picea abies*; Sm-*Selaginella meollendorffii;* Pp*-Physcomitrella patens;* Mp*-Marchantia polymorpha;* Nm*-Nitella mirabilis;* Kf-*Klebsormidium flaccidium;* Mr.*-Micromonas RCC299;* Mpu*-Micromonas pusila;* Ot*-Ostreococcus tauri; Ol-Ostreococcus lumiferans;* Cv*-Chlorella vulgaris; Cr-Chlamydomonas reinhardtii;* Vc*-Volvox carteri;* Cp*-Cyanophora paradoxa.* Plus sign (+) and minus sign (−) indicate presence and absence of an above mentioned domain architecture

### Class IV-suppressor of variegation Homologs and relatives Su(var)3–9

**Fig. 6:**
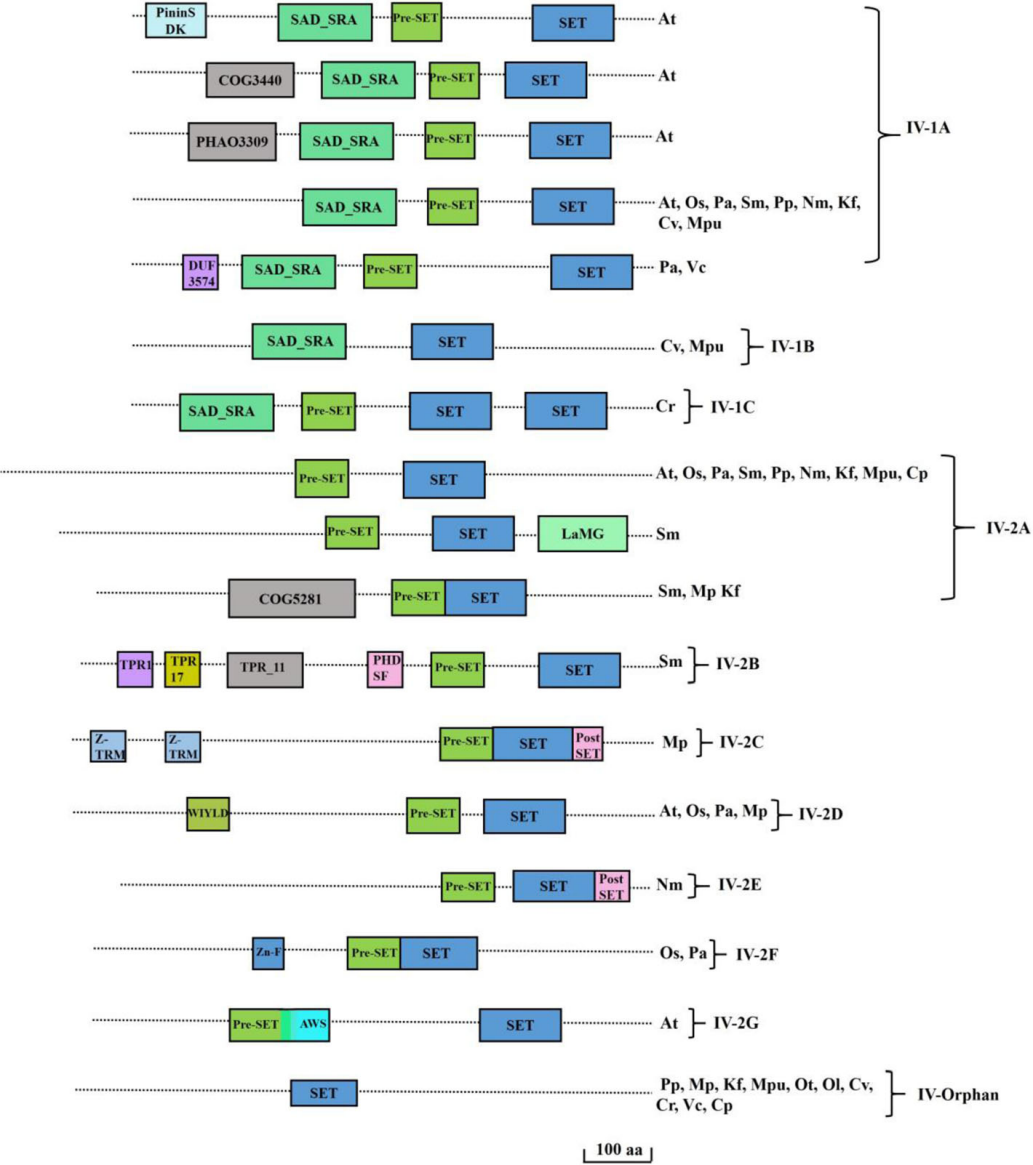
Domain organisations of the Su(var)3–9 family. Su(var)3–9 family proteins are grouped into two groups and further subgroups to a total of 17 different domain combinations. Species sharing the specific domain arrangement were indicated on right-hand side. Different protein domains are colored differently as indicated. SRA: YDG-SET and ring finger associated; TPR: Tetratricopeptide repeat; Z-TRM: tRNA 2′-O-methyltransferase Trm13; WIYLD: Ubiquitin-binding WIYLD domain; DUF3574: Domain of unknown function; COG5281: Phage related minor tail protein; LaMG: Laminin G domain; UBA: Ubiquitin associated domain. Domains are not drawn to scale. Scale bars indicate 100 amino acids. (At- *Arabidopsis thaliana*; Os-*Oryza sativa*; Pa-*Picea abies*; Sm-*Selaginella meollendorffii;* Pp*-Physcomitrella patens;* Mp*-Marchantia polymorpha;* Nm*-Nitella mirabilis;* Kf-*Klebsormidium flaccidium;* Mr.*-Micromonas RCC299;* Mpu*-Micromonas pusila;* Ot*-Ostreococcus tauri; Ol-Ostreococcus lumiferans;* Cv*-Chlorella vulgaris; Cr-Chlamydomonas reinhardtii;* Vc*-Volvox carteri;* Cp*-Cyanophora paradoxa*)

Su(var) comprises of a large group of proteins characterized by the presence of PreSET, SET and PostSET domains. This class is designated here as Class IV (Fig. 6). This class is divided in to two - IV-1 & 2. IV-1 is subdivided into IV-1A to C. IV-2 is subdivided into IV-2A to G. Most members of Class IV-1 has a characteristic arrangement of SET and Ring finger Associated (SAD_SRA) domain followed by a PreSET located N terminal to the SET domain. Only exception is the class IV-1B where PreSET domain is absent in between SAD_SRA and SET domain. SAD_SRA domain binds to methylated cytosines [26]. Class IV-1B proteins are present in *Chlorella vulgaris* and *Micromonas pusila*. Class IV-C possess two SET domains located C-terminal to SAD_SRA and PreSET domain. This class of protein is present in *Chlamydomonas reinhardtii*. All members of class IV-2 has a PreSET domain before SET domain with the absence of SAD_SRA domain. Class IV-2C contains one or more ZnF_C2H2 domain(s) at its N-terminus and only found in *Marchantia polymorpha* whereas Class IV-2D contains one Ubiquitin-binding WIYLD domain (WIYLD) at its N-terminus along with PreSET and SET, found in *Arabidopsis thaliana*, *Oryza sativa*, *Picea abies*, and *Marchantia polymorpha* (Table 3). This domain organization might have arisen relatively recently in *Marchantia polymorpha,* belonging to embryophyta (Additional file 6: Figure S4). It is worth noting that unlike we see that SRA_SET is already present in chlorophyte contrary to the previous report of its first appearance in bryophytes [27]. Our analysis shows that PostSET domain is present only in *Marchantia polymorpha* and *Nitella mirabilis,* categorized under Class IV-2C and Class IV-2E respectively. PostSET domain might have been lost by some members during the subsequent evolution (Additional file 6: Figure S4).

**Table 3:** Classification of Class IV-Su(var) proteins

Class		Type	Domain Architecture	Species																Extra Domains
				At	Os	Pa	Sm	Pp	Mp	Nm	Kf	Mr	Mpu	Ot	Ol	Cv	Cr	Vc	Cp	
IV	IV-1	1A	SRA-PreSET-SET	+	+	+	+	+	–	+	+	–	+	–	–	+	+	+	–	DUF3574, Treacle, PPR, Pinin_SDK, COG3440
		1B	SRA-SET	–	–	–	–	–	–	–	–	–	+	–	–	+	–	–	–	
		1C	SRA-PreSET-SET-SET	–	–	–	–	–	–	–	–	–	–	–	–	–	+	–	–	
	IV-2	2A	PreSET-SET	+	+	+	+	+	+	+	+	–	+	–	–	–	–	–	–	LaMG, COG5281
		2B	PHD-PreSET-SET	–	–	–	+	–	–	–	–	–	–	–	–	–	–	–	–	TPR
		2C	ZnF_C2H2-ZnF_C2H2-PreSET-SET-PostSET	–	–	–	–	–	+	–	–	–	–	–	–	–	–	–	–	
		2D	WIYLD-PreSET-SET	+	+	+	–	–	+	–	–	–	–	–	–	–	–	–	–	DUF5102, CNDH2_M
		2E	PreSET-SET-PostSET	–	+	–	–	–	–	+	–	–	–	–	–	–	–	–	–	
		2F	ZnF_PreSET-SET	–	+	+	–	–	–	–	–	–	–	–	–	–	–	–	–	
		2G	PreSET-AWS-SET	+	–	–	–	–	–	–	–	–	–	–	–	–	–	–	–	
	IV-Orphan		SET	–	–	–	–	+	+	–	+	–	+	+	+	+	+	+	+	

Su(var) proteins were classified into 3 classes, Class IV-1, Class IV-2 and Class Orphan which were further divided into subclasses. The detailed domain arrangement with additional domains are shown. (At- *Arabidopsis thaliana*; Os-*Oryza sativa*; Pa-*Picea abies*; Sm-*Selaginella meollendorffii;* Pp*-Physcomitrella patens;* Mp*-Marchantia polymorpha;* Nm*-Nitella mirabilis;* Kf-*Klebsormidium flaccidium;* Mr.*-Micromonas RCCC;* Mpu*-Micromonas pusila;* Ot*-Ostreococcus tauri; Ol-Ostreococcus lumiferans;* Cv*-Chlorella vulgaris; Cr-Chlamydomonas reinhardtii;* Vc*-Volvox carteri;* Cp*-Cyanophora paradoxa.* Plus sign (+) and minus sign (−) indicate presence and absence of an above mentioned domain architecture

### Class V-orphan, SETD and TPR

Class V category of proteins share a low level of sequence identity to conserved SET domain proteins, hence are grouped separately. Class V comprises of Orphan proteins, SETD and TPR. Diverse associated domains found in other groups are absent in Class V (Additional file 7: Figures S5, and Additional file 8: Figure S6). Few members also exhibit interrupted SET domain. Many unique domains in the Orphan group such as Regulator of chromosome condensation (RCC1); bHLH-MYC (Basic helix loop helix binding to MYC transcription factors); HLH (Helix loop helix); BASP1 (Brain acid soluble protein 1), Ribosomal protein L1(rp1A) and DUF4239 (Domain of unknown function) are found that are not common in other SET domain proteins [28–30]. SETD proteins contain a Rubisco Lysine Serine Methyl Transferase (LSMT) substrate binding domain, which allows the protein to bind to the N-terminal tails of histones H3 and H4 [31]. The SET domain of SETD also has highly divergent sequences compared to canonical SET proteins and propose to methylate non-histone targets [32, 33]. (Additional file 7: Figures S5, and Additional file 8: Figure S6). Nevertheless, the specific methylation features within the SET region are retained [31, 32]. TPR group of proteins too lack a significant homology to canonical SET domain sequence. This protein family has a unique domain of tetratricopeptide repeats of a minimum of 34 amino acids.

## Discussion

In the present study, we have computationally identified 506 SET domain proteins from the published genomes and transcriptomes of 16 members of plant lineage Archaeplastida. We have performed phylogenetic analysis of SET domains and classify proteins according to their domain organisations. Published SET domain phylogenies of rice and *Arabidopsis* are used as template [14, 15, 19]. This classification may reflect functional relatedness and is a basis to choose candidates to experimentally elucidate functions of SET domain proteins. In future it will be interesting to see if the loss and gain of various domains in SET domain proteins presented here is relevant to growth pattern, habitat, and alteration of generation in evolutionary history of plant lineage. Introduction of PHD domain in Ash group of proteins, Zn finger C2H2 in Trx group and PostSET in Su(var) in charophytes prompts to speculate these changes in aquatic to benthic lifestyle.

Like Arabidopsis, rice and other flowering plants plant lineage appears to have relatively higher number of SET domain proteins than yeasts and animals [15, 33]. Interestingly, numbers of individual class of proteins vary dramatically in different plant groups indicating lineage specific or redundant functions of these proteins.

Plant SET domain proteins have fewer domains. It might be that in plant lineage, domains remains dispersed in larger number of proteins. Tesmin/TSO1(TCR) domain is specifically associated with E(z) class of proteins. Glaucophyte neither have E(z) proteins nor TCR in SET domain proteins.

Other interesting feature in the evolution of the E(z) protein is the presence of the Swi3, Ada2, N-Cor, and TFIIIB (SANT) domain only in rice is indicative of monocot specific function. Aldehyde dehydrogenase superfamily (ALDH-SF); Cysteine/serine-rich nuclear protein (CSR); Male-specific lethal (MSI) are biochemically uncharacterized domains associated with SET domains of E (z) members.

Ash class of proteins have varied domain combinations, therefore we have newly classified them. Except in orphans, AWS domain is very tightly associated N terminally to the SET domain with Ash class of proteins. An interesting feature is the presence of single PHD domain in class II-2B whereas three of them on a protein in class II-2A. TUDOR domain is reported to be absent from plant lineage but we confirm its presence in Ash class proteins of *Selaginella meollendorffii* and *Marchantia polymorpha* [19]*.* This domain is also present in Trx class of proteins.

In general Trx class of proteins have larger number of domains. This is the trend towards SET domain proteins of animals. Predominantly PWWP and PHD domains are associated with Trx class of proteins. TUDOR domain has already appeared in SET domain protein in chlorophyta.

Phylogenetic classification of Su(var)3–9 SET proteins of Archaeplastida into two classes is based on the presence or absence of SRA domains. Our analysis reveal WIYLD and Zinc Finger domains of Class IV to have originated in marchantiophyta (*Marchantia polymorpha*). SRA domain emerged as early as chlorophyta and PostSET domain of Su(var)3–9 SET traced back to charophytes and then lost in some members during the subsequent evolution. (Fig. 6, Additional file 6: Figure S4, Table 3). We speculate that higher number of Su(var)3–9 SET proteins in flowering plant species is due to recent duplications after their divergence from other land plants and they play role in plant development related to flowering. This is consistent with role of one of the member of this class, KRYPTONITE in flower development [34]*.*

In context to the diversity of life forms and its connection with the evolution of epigenetic regulations, it is noteworthy that the complexification of a species in terms of multicellularity, habitat, and forms are supposedly not connected with the epigenetic regulations. This analysis suggests that diversification of the SET domain proteins may not be the sole determinant related to the different biology of the species. Another possibility might be that orthologous proteins play diverse roles in different species. Therefore, it appears that during evolution, an additional domain are acquired along with the SET domain to enable supplementary functions. Hence, we believe that the present identification and classification of SET domain proteins of Archaeplastida will accelerate biochemical characterization of these proteins.

## Conclusions

We have identified and analyzed 506 SET domain proteins from 16 members of Archaeplastida through sequence homology and phylogenetic approach. We grouped these SET domain proteins into five classes- E(z), Ash, Trx, Su(var) and Orphan. Our work provided framework for experimental characterization of plant SET domain proteins.

## Additional files

Additional file 1: Table S1. List of species considered in the present study with the corresponding number of SET domain containing protein. (PDF 10 kb)

Additional file 2: Table S2. List of the SET domain containing proteins from species considered in the present study with their corresponding sequence id. (PDF 65 kb)

Additional file 3: Figure S1. Multiple sequence alignment of the 251 SET domain protein sequences from E(z), Ash, Trx and Su(var) of the 16 Archeplastida species. (PDF 74 kb)

Additional file 4: Figure S2. Introduction of the domains in the Ash SET protein in cryptogam plant lineages. The black arrow indicates the introduction of the indicated domain in the specifically mentioned Archaeplastida species. (PDF 212 kb)

Additional file 5: Figure S3. Introduction of the domains in the Trx SET protein in plant lineages. The black arrow indicates the introduction of the indicated domain in the specifically mentioned Archaeplastida species. (PDF 221 kb)

Additional file 6: Figure S4. Introduction of the domains in the Su(var) SET protein in plant lineages. The black arrow indicates the introduction of the indicated domain in the specifically mentioned Archaeplastida species. (PDF 85 kb)

Additional file 7: Figure S5. Schematic diagrams showing the domain organization of Orphan proteins. (PDF 550 kb)

Additional file 8: Figure S6. Domain architecture of Class V- A) SETD and B) TPR family. (PDF 507 kb)
