# Supplementary material for: Phylogenetic relationship and domain organisation of SET domain proteins of Archaeplastida
Source: BMC Plant Biol. 2017 Dec 11;17:238. doi: 10.1186/s12870-017-1177-1 (PMC5725981; doi:10.1186/s12870-017-1177-1)
Supplement: Supplementary file 1 — List of species considered in the present study with the corresponding number of SET domain containing protein. (PDF 10 kb) [file 12870_2017_1177_MOESM1_ESM.pdf]

Additional file 1: Table S1.

Sl. No.	Archaeplastida species	Species Abbreviation	Number of proteins
1.	*Arabidopsis thaliana*	At	41
2.	*Oryza sativa*	Os	41
3.	*Pinus abies*	Pa	36
4.	*Selaginella moellendorffii*	Sm	51
5.	*Physcomitrella patens*	Pp	46
6.	*Marchantia polymorpha*	Mp	32
7.	*Nitella mirabilis*	Nm	21
8.	*Klebsormidium flaccidum*	Kf	34
9.	*Micromonas RCC299*	Mr	40
10.	*Micromonas pusilla*	Mpu	27
11.	*Ostreococcus tauri*	Ot	22
12.	*Ostreococcus lucimarinus*	Ol	20
13.	*Chlorella vulgaris*	Cv	22
14.	*Chlamydomonas reinhardtii*	Cr	27
15.	*Volvox carteri*	Vc	30
16.	*Cyanophora paradoxa*	Cp	16

List of species considered in the present study with the corresponding number of SET domain containing protein.
