# Supplementary material for: Phylogenetic relationship and domain organisation of SET domain proteins of Archaeplastida
Source: BMC Plant Biol. 2017 Dec 11;17:238. doi: 10.1186/s12870-017-1177-1 (PMC5725981; doi:10.1186/s12870-017-1177-1)
Supplement: Supplementary file 2 — List of the SET domain containing proteins from species considered in the present study with their corresponding sequence id. (PDF 65 kb) [file 12870_2017_1177_MOESM2_ESM.pdf]

Additional file 2: Table S2.

Sl. No.	Name	Sequence id	Sl. No.	Name	Sequence id
1.	At.CLF	AT2G23380	28.	At. Suvh6	AT2G22740
2.	At.MEA	AT1G02580	29.	At. Suvh7	AT1G17770
3.	At.SWN	AT4G02020	30.	At. Suvh8	AT2G24740
4.	At.Ashh1	AT1G76710	31.	At. Suvh9	AT4G13460
5.	At.Ashh2	AT1G77300	32.	At. Suvh10	AT2G05900
6.	At.Ashh3	AT2G44150	33.	At. Suvr1	AT1G04050
7.	At.Ashh4	AT3G59960	34.	At. Suvr2	AT5G43990
8.	At.Ashr1	AT2G17900	35.	At. Suvr3	AT3G03750
9.	At.Ashr2	AT2G19640	36.	At. Suvr4	AT3G04380
10.	At.Ashr3	AT4G30860	37.	At. Suvr5	AT2G23740
11.	At.Atx1	AT2G31650	38.	At.Orphan	At5G17240
12.	At.Atx2	AT1G05830	39.	At.Rubisco.1	AT1G24610
13.	At.Atx3	AT3G61740	40.	At.Rubisco.2	AT3G07670
14.	At.Atx4	AT4G27910	41.	At.Rubisco.3	AT4G20130
15.	At.Atx5	AT5G53430	42.	Os.CLF	LOC_OS06g16390
16.	At.Atxr1	AT1G26760	43.	Os.SWN	LOC_OS03g19480
17.	At.Atxr2	AT3G21820	44.	Os.Ashh1	LOC_OS04g34976
18.	At.Atxr3	AT4G15180	45.	Os.Ashh2	LOC_Os02g34850
19.	At.Atxr4	AT5G06620	46.	Os.Ashh3a	LOC_Os08g34370
20.	At.Atxr5	AT5G09790	47.	Os.Ashh3b	LOC_Os02g39800
21.	At.Atxr6	AT5G24330	48.	Os.Ashh3c	LOC_Os09g13740
22.	At.Atxr7	AT5G42400	49.	Os.Atx1	LOC_Os09g04890
23.	At. Suvh1	AT5G04940	50.	Os.Atx4	LOC_Os01g11952
24.	At. Suvh2	AT2G33290	51.	Os.Atx5	LOC_Os01g46700
25.	At. Suvh3	AT1G73100	52.	Os.Atx6	LOC_Os09g38440
26.	At. Suvh4	AT5G13960	53.	Os.Atxr1	LOC_Os03g07260
27.	At. Suvh5	AT2G35160	54.	Os.Atxr2	LOC_Os04g53700

Sl. No.	Name	Sequence id	Sl. No.	Name	Sequence id
55.	Os.Atxr3	LOC_Os08g08210	82.	Pa.EZA2	MA_7025g0010
56.	Os.Atxr4	LOC_Os10g27060	83.	Pa.Ashh1	MA_91554g0010
57.	Os.Atxr5	LOC_Os01g73460	84.	Pa.Atx1	MA_10435027g0010
58.	Os.Atxr6	LOC_Os02g03030	85.	Pa.Atx4	MA_908464g0010
59.	Os.Atxr7	LOC_OS12g41900	86.	Pa.Atx5	MA_10359605g0020
60.	Os.Suvh1	LOC_Os11g38900	87.	Pa.Atx6	MA_73622g0010
61.	Os.Suvh2	LOC_OS07g25450	88.	Pa,.Atxr6a	MA_125231g0010
62.	Os.Suvh4	LOC_OS01g70220	89.	Pa,.Atxr6b	MA_125231g0010
63.	Os.Suvh5a	LOC_OS04g45990	90.	Pa.Atxr7	MA_18041g0010
64.	Os.Suvh5b	LOC_OS09g19830	91.	Pa.Suvh2a	MA_24152g0010
65.	Os.Suvh5c	LOC_OS08g30910	92.	Pa.Suvh2b	MA_790260g0010
66.	Os.Suvh7	LOC_OS01g59620	93.	Pa.Suvh2c	MA_149700g0010
67.	Os.Suvh8	LOC_OS05g41172	94.	Pa.Suvh4	MA_22288g0010
68.	Os.Suvh9	LOC_OS08g45130	95.	Pa.Suvh5a	MA_106068g0010
69.	Os.Suvh10	LOC_Os01g56540.1	96.	Pa.Suvh5b	MA_10428046g0010
70.	Os.Suvh11	LOC_OS11g03700	97.	Pa.Suvh7a	MA_26178g0010
71.	Os.Suvr4a	LOC_OS02g40770	98.	Pa.Suvh7b	MA_7658g0020
72.	Os.Suvr4b	B8AS58.1/577-699	99.	Pa.Suvh7c	MA_54295g0010
73.	Os.Suvr5	LOC_OS02g47900	100.	Pa.Suvh10a	MA_77020g0010
74.	Os.Orphan.1	Q8LN53.1/26-252	101.	Pa.Suvh10b	MA_10076925g0010
75.	Os.Orphan.2	Q7XJY0.1/95-296	102.	Pa.Suvr3	MA_604033g0010
76.	Os.Orphan.3	Q6ZCF7.1/15-191	103.	Pa.Suvr4	MA_106792g0010
77.	Os.Orphan.4	Q6ZCF6.1/15-252	104.	Pa.Suvr5a	MA_10428226g0010
78.	Os.Orphan.5	Q0JD76.1/4-296	105.	Pa.Suvr5b	MA_10428046g0010
79.	Os.Rubisco.1	B8AHQ1.1/98-303	106.	Pa.Orphan.1	MA_9310110g0010
80.	Os.Rubisco.2	B8AI25.1/86-294	107.	Pa.Orphan.2	MA_8913147g0010
81.	Pa.EZA1	MA_10430132g0010	108.	Pa.Orphan.3	MA_197676g0010

Sl. No.	Name	Sequence id	Sl. No.	Name	Sequence id
109.	Pa.Orphan.4	MA_116780g0010	138.	Sm.Suvr5	D8SSK6
110.	Pa.Orphan.5	MA_10429168g0010	139.	Sm.Orphan.1	D8QMX5
111.	Pa.Orphan.6	MA_102811g0010	140.	Sm.Orphan.2	D8QPY7
112.	Pa.Orphan.7	MA_102702g0010	141.	Sm.Orphan.3	D8QPZ1
113.	Pa.Orphan.8	MA_84666g0010	142.	Sm.Orphan.4	D8QQY8
114.	Pa.Orphan.9	MA_89572g0010	143.	Sm.Orphan.5	D8R4Y0
115.	Pa.Orphan.10	MA_2465g0010	144.	Sm.Orphan.6	D8RGR5
116.	Pa.Orphan.11	MA_22501g0010	145.	Sm.Orphan.7	D8RV13
117.	Sm.CLF	D8S9D0	146.	Sm.Orphan.8	D8SCX3
118.	Sm.Ashr1	D8RF07	147.	Sm.Orphan.9	D8SD75
119.	Sm.Ashr2a	D8RED9	148.	Sm.Orphan.10	D8SKI0
120.	Sm.Ashr3	D8TFC7	149.	Sm.Orphan.11	D8T2D8
121.	Sm.Ashr2b	D8QVC8	150.	Sm.Orphan.12	D8T3Q4
122.	Sm.Ashh1	D8RRM9	151.	Sm.Orphan.13	D8T5D5
123.	Sm.Ashh2c	D8SGM1	152.	Sm.Orphan.14	D8T9U6
124.	Sm.Atx1	D8T9E2	153.	Sm.Rubisco.1	D8R0C9
124.	Sm.Atx2a	D8QZH7	154.	Sm.Rubisco.2	D8RS19
125.	Sm.Atx2b	D8RVW3	155.	Sm.Rubisco.3	D8SX31
126.	Sm.Atx2c	D8T9E2	156.	Sm.Rubisco.4	D8RS19
127.	Sm.Atx5a	D8QTI2	157.	Sm.TPR.1	D8QRY2
128.	Sm.Atx5b	D8QYK5	158.	Sm.TPR.2	D8QRY3
129.	Sm.Atxr2	D8R6G9	159.	Sm.TPR.3	D8QRY5
130.	Sm.Atxr7	D8QUH4	160.	Sm.TPR.4	D8QWB8
131.	Sm.Suvh2	D8RL88	161.	Sm.TPR.5	D8RLH2
132.	Sm.Suvh4	D8QYE1	162.	Sm.TPR.6	D8RRN0
133.	Sm.Suvh5	D8R2J4	163.	Sm.TPR.7	D8S7L0
134.	Sm.Suvh6	D8S8V8	164.	Sm.TPR.8	D8RRM9
135.	Sm.Suvh10	D8RL84	165.	Sm.TPR.9	D8SJZ3
136.	Sm.Suvr1	D8R6H6	166.	Sm.TPR.10	D8T899
137.	Sm.Suvr3	D8R0G6	167.	Pp.CLF	A9SP73

Sl. No.	Name	Sequence id	Sl. No.	Name	Sequence id
169.	Pp.Ashh2a	A9T8D5	196.	Pp.Orphan.3	A9RMX3
170.	Pp.Ashh2b	A9RU55	197.	Pp.Orphan.4	A9RZ51
171.	Pp.Ashr1	A9THX1	198.	Pp.Orphan.5	A9SV96
172.	Pp.Ashr3a	A9SPZ8	199.	Pp.Orphan.6	A9SV99
173.	Pp.Ashr3b	A9SGL5	200.	Pp.Orphan.7	A9SXI4
174.	Pp.Atxa	A9RQ81	201.	Pp.Orphan.8	A9SXS8
175.	Pp.Atxb	A9RXF6	202.	Pp.Orphan.9	A9SZA6
176.	Pp.Atxc	A9SIE6	203.	Pp.Orphan.10	A9T182
177.	Pp.Atxd	A9SJ97	204.	Pp.Orphan.11	A9TLI8
178.	Pp.Atxe	A9SM72	205.	Pp.Orphan.12	A9TT20
179.	Pp.Atxf	A9TPQ7	206.	Pp.Orphan.13	A9U0J8
180.	Pp.Atxr5	A9TE94	207.	Pp.Orphan.14	A9SZA6
181.	Pp.Atxrb	A9TG42	208.	Pp.Orphan.15	A9T182
182.	Pp.Atxr7a	A9SPD9	209.	Pp.Orphan.16	A9TLI8
183.	Pp.Atxr7b	A9SXI4	210.	Pp.Rubisco.1	A9TYS2
184.	Pp.Suvr3a	A9RDH9	211.	Pp.Rubisco.2	A9RJA3
185.	Pp.Suvr3b	A9RRR7	212.	Pp.Rubisco.3	A9RZT2
186.	Pp.Suvr4b	A9RS53	213.	Pp.TPR.1	A9RJ30
187.	Pp.Suvr5a	A9TUJ1	214.	Pp.TPR.2	A9TID7
188.	Pp.Suvr5b	A9T6Q6	215.	Pp.TPR.3	A9U604
189.	Pp.Suvr5c	A9SP28	216.	Mp.CLF	OAE22047
190.	Pp.Suvr4a	A9U4M6	217.	Mp.MEA	OAE18855
191.	Pp.Suvh4b	A9U3I2	218.	Mp.EZA	OAE28386
192.	Pp.Suvh4a	A9U327	219.	Mp.Ashh1	OAE25419
193.	Pp.Orphan.1	A9TYS2	220.	Mp.Ashh2a	OAE31350
194.	Pp.Orphan.2	A9RJU7	221.	Mp.Ashh2b	OAE30629
195.	Pp.Orphan.2	A9RJU7	222.	Mp.Ash3a	OAE29839

Sl. No.	Name	Sequence id	Sl. No.	Name	Sequence id
223.	Mp.Ashr3a	OAE23350	253.	Nm.Ashh2a	Gene.61624
224.	Mp.Ashh3b	OAE30629	254.	Nm.Atxa	Gene.59661
225.	Mp.Atxr7	OAE29888	255.	Nm.Atxb	Gene.41564
226.	Mp.Atxra	OAE25983	256.	Nm.Atx5	Gene.73511
227.	Mp.Atx2	OAE25819	257.	Nm.Atxr5	Gene.64583
228.	Mp.Atxr2	OAE34582	258.	Nm.Atxr7	Gene.55137
229.	Mp.Atx5	OAE29150	259.	Nm.Suvr4	Gene.70782
230.	Mp.Suvr3	OAE24745	260.	Nm.Suvr5a	Gene.56032
231.	Mp.Suvr4	OAE22194	261.	Nm.Suvr5b	Gene.68420
232.	Mp.Suvr5a	OAE19953	262.	Nm.Suvh4	Gene.54286
233.	Mp.Suvr5b	OAE22758	263.	Nm.Orphan.1	Gene.31202
234.	Mp.Suvr5c	OAE19953	264.	Nm.Orphan.2	Gene.72380
235.	Mp.Orphan.1	OAE30793	265.	Nm.Orphan.3	Gene.48604
236.	Mp.Orphan.2	OAE22154	266.	Nm.Orphan.4	Gene.23543
237.	Mp.Rubisco.1	OAE30725	267.	Nm.Orphan.5	Gene.41487
238.	Mp.Rubisco.2	OAE28872	268.	Nm.Orphan.6	Gene.56815
239.	Mp.Rubisco.3	OAE24941	269.	Kf.EZA	kfl00079_0080
240.	Mp.TPR.1	OAE33201	270.	kf.Ashh1	kfl00373_0160
241.	Mp.TPR.2	OAE25709	271.	kf.Ashh4a	kfl00081_0090
242.	Mp.TPR.3	OAE20475	272.	kf.Ashh4b	kfl00076_0310
243.	Mp.TPR.4	OAE33527	273.	kf.Atxa	kfl00733_0020
244.	Mp.TPR.5	OAE35631	274.	kf.Atxb	kfl00346_0030
245.	Mp.TPR.6	OAE21964	275.	kf.Atxr3	kfl00127_0110
246.	Mp.TPR.7	OAE23736	276.	kf.Atxr7a	kfl00067_0030
247.	Mp.TPR.8	OAE28902	277.	kf.Atxr7b	kfl00081_0100
248.	Nm.CLF	Gene.62250	278.	Kf.Suvr3	kfl00005_0100
249.	Nm.Ashh2b	Gene.73939	279.	Kf.Suvr4	kfl00140_0100
250.	Nm.Ashr2	Gene.66948	280.	kf.Suvh4a	kfl00217_0080
251.	Nm.Ashr3	Gene.29565	281.	Kf.Suvh4b	kfl00415_0100
252.	Nm.Atxr2	Gene.75130	282.	Kf.Orphan.1	kfl00205_0110

Sl. No.	Name	Sequence id	Sl. No.	Name	Sequence id
283.	Kf.Orphan.2	kfl00636_0060	313.	Mr.Orphan.7	C1EGG0
284.	Kf.Orphan.3	kfl00476_0020	314.	Mr.Orphan.8	C1EIX1
285.	Kf.Orphan.4	kfl00289_0080	316.	Mr.Orphan.9	C1EIY0
286.	Kf.Orphan.5	kfl00024_0470	317.	Mr.Orphan.10	C1EJ93
287.	Kf.Orphan.6	kfl00810_003	318.	Mr.Orphan.11	C1FG43
288.	Kf.Orphan.7	kfl00328_0090	319.	Mr.Orphan.12	C1FGI0
289.	Kf.Orphan.8	kfl00052_0360	320.	Mr.Orphan.13	C1DXZ9
290.	Kf.Orphan.9	kfl00199_0070	321.	Mr.Orphan.14	C1DY71
291.	Kf.Orphan.10	kfl00823_0040	322.	Mr.Orphan.15	C1DZ87
292.	Kf.Orphan.11	kfl00387_0040	323.	Mr.Orphan.16	C1E2J3
293.	Kf.Orphan.12	kfl00057_0280	324.	Mr.Orphan.17	C1E382
294.	Kf.Orphan.13	kfl00212_0140	325.	Mr.Orphan.18	C1E668
295.	Kf.Orphan.14	kfl00084_0180	326.	Mr.Orphan.19	C1E6T9
296.	Kf.Orphan.15	kfl00289_0080	327.	Mr.Orphan.20	C1E8U5
297.	Kf.Orphan.16	kfl00476_0040	328.	Mr.Orphan.21	C1EAU4
298.	Kf.Orphan.17	kfl00081_0100	329.	Mr.Orphan.22	C1ED04
299.	Kf.Orphan.18	kfl00199_0070	330.	Mr.Orphan.23	C1FGT6
300.	Kf.Orphan.19	kfl00823_0040	331.	Mr.Orphan.24	C1FHY4
301.	Mr.CLF	C1EG84	332.	Mr.Orphan.25	C1FI20
302.	Mr.Ashh1	C1ECR2	333.	Mr.Orphan.26	C1FIJ2
303.	Mr.Ashh2a	C1E8P0	334.	Mr.Orphan.27	C1DZ87
304.	Mr.Ashh2b	C1E7A5	335.	Mr.Orphan.28	C1FI08
305.	Mr.Ashr1a	C1E7A5	336.	Mr.Orphan.29	C1FI43
306.	Mr.Atx5	C1DZT5	337.	Mr.Rubisco.1	C1E9B1
307.	Mr.Orphan.1	C1DXZ9	338.	Mr.Rubisco.2	C1EGH5
308.	Mr.Orphan.2	C1DY71	339.	Mr.Rubisco.3	C1FH92
309.	Mr.Orphan.3	C1E2J3	340.	Mr.TPR.1	C1EAF2
310.	Mr.Orphan.4	C1E4M9	341.	Mr.TPR.2	C1EDU7
311.	Mr.Orphan.5	C1E640	342.	Mpu.CLF	C1MVG4
312.	Mr.Orphan.6	C1EGG0	343.	Mpu.Atx5	C1N3U5

Sl. No.	Name	Sequence id	Sl. No.	Name	Sequence id
344.	Mpu.Atxr5	C1N497	373.	Ot.Ashh2	A0A096P8M9
345.	Mpu.Atxr	C1MUM1	374.	Ot.Atx	A0A096P7N1
346.	Mpu.Atxr1	C1MPU8	375.	Ot.Orphan.1	A0A096PAR9
347.	Mpu.Suvr3	C1N2W7	376.	Ot.Orphan.2	Q00T11
348.	Mpu.Suvh4	C1N8R6	377.	Ot.Orphan.3	Q016D2
349.	Mpu.Suvr5	C1MIM2	378.	Ot.Orphan.4	A0A090M1Z0
350.	Mpu.Suvh10	C1MNX1	379.	Ot.Orphan.5	A0A090M3L6
351.	Mpu.Orphan.1	C1MUX9	380.	Ot.Orphan.6	A0A090N324
352.	Mpu.Orphan.2	C1MXS8	381.	Ot.Orphan.7	A0A096P7V0
353.	Mpu.Orphan.3	C1MYR2	382.	Ot.Orphan.8	A0A090M6A5
354.	Mpu.Orphan.4	C1MZZ6	383.	Ot.Orphan.9	Q00VK7
355.	Mpu.Orphan.5	C1N2K0	384.	Ot.Orphan.10	A0A090M6E6
356.	Mpu.Orphan.6	C1N4C0	385.	Ot.Orphan.11	A0A090N324
357.	Mpu.Orphan.7	C1MMM8	386.	Ot.Rubisco.1	A0A090M1H3
358.	Mpu.Orphan.8	C1MQU3	387.	Ot.Rubisco.2	A0A096P8D4
359.	Mpu.Orphan.9	C1MS34	388.	Ot.Rubisco.3	A0A096PAX8
360.	Mpu.Orphan.10	C1MT33	389.	Ot.TPR.1	Q00Z98
361.	Mpu.Orphan.11	C1MJ25	390.	Ol.CLF	A4SB06
362.	Mpu.Orphan.12	C1MGP5	391.	Ol.Ashr2	A4RWK6
363.	Mpu.Orphan.13	C1MHE0	392.	Ol.Ashh2	A4S6X8
364.	Mpu.Orphan.14	C1MJ95	393.	Ol.Ashh4	A4S9D3
365.	Mpu.Orphan.15	C1MM13	394.	Ol.Atxrb	A4S6Z7
366.	Mpu.Orphan.16	C1MMM2	395.	Ol.Atxr3	A4RTZ1
367.	Mpu.Rubisco.1	C1MLT4	396.	Ol.Orphan.1	A4RYG6
368.	Mpu.Rubisco.2	C1MSM7	397.	Ol.Orphan.2	A4RZG0
369.	Ot.CLF	A0A096PBK5	398.	Ol.Orphan.3	A4S531
370.	Ot.Ashh1	Q00WL8	399.	Ol.Orphan.4	A4S8I0
371.	Ot.Ashr1	Q018N4	400.	Ol.Orphan.5	A4RVA6
372.	Ot.Ashr2	A0A096PA69	401.	Ol.Orphan.6	A4RZG0

Sl. No.	Name	Sequence id	Sl. No.	Name	Sequence id
402.	Ol.Orphan.7	A4S531	432.	Cr.Ashh2	A8J069
403.	Ol.Orphan.8	A4S8I0	433.	Cr.Ashr1	A8HPL7
404.	Ol.Orphan.9	A4S9K0	434.	Cr.Atxr	A8JFP3
405.	Ol.Rubisco.1	A4RXQ6	435.	Cr.Atxr1	A8IWW5
406.	Ol.Rubisco.2	A4S2V8	436.	Cr.Atxr7	A8JHD4
407.	Ol.Rubisco.3	A4S7V0	437.	Cr.Suv	A8I904
408.	Ol.Rubisco.4	A4S805	438.	Cr.Orphan.1	ASHMJ9
409.	Ol.TPR.1	A4S4E3	439.	Cr.Orphan.2	A8JFC2
410.	Cv.CLF	E1ZI62	440.	Cr.Orphan.3	A8JIA0
411.	Cv.Ashh2	E1Z3C6	441.	Cr.Orphan.4	A8JEB7
412.	Cv.Ashh4	E1ZH19	442.	Cr.Orphan.5	A8JCM2
413.	Cv.Ashr1a	E1ZMK7	443.	Cr.Orphan.6	A8J252
414.	Cv.Ashr2b	E1ZK66	444.	Cr.Orphan.7	A8J215
415.	Cv.Atxr2	E1ZTC5	445.	Cr.Orphan.8	A8IYY9
416.	Cv.Suvh2	E1Z7S7	446.	Cr.Orphan.9	A8IQ95
417.	Cv.Suvr3	E1Z4Y1	447.	Cr.Orphan.10	A8HSP6
418.	Cv.Suvh4	E1ZIE0	448.	Cr.Orphan.11	A8HTU8
419.	Cv.Orphan.1	E1ZCG6	449.	Cr.Orphan.12	A8IFJ6
420.	Cv.Orphan.2	E1ZPL6	450.	Cr.Orphan.13	A8J457
421.	Cv.Orphan.3	E1ZR56.	451.	Cr.Rubisco.1	A8J093
422.	Cv.Orphan.4	E1ZCE2	452.	Cr.Rubisco.2	A8JAC4
423.	Cv.Orphan.5	E1ZCE2	453.	Cr.Rubisco.3	A8JHE6
424.	Cv.Rubisco.1	E1Z7M4	454.	Cr.TPR.1	A8I6N2
425.	Cv.Rubisco.2	E1ZKX8	455.	Cr.TPR.2	A8I6N5
426.	Cv.Rubisco.3	E1ZLY3	456.	Cr.TPR.3	A8I6N8
427.	Cv.Rubisco.4	E1ZR21	457.	Vc.Ashr1b	D8UDD9
428.	Cv.Rubisco.5	E1ZTR9	458.	Vc.Ashr2	D8TS63
429.	Cv.TPR.1	E1Z212	459.	Vc.Ashh1	D8U7V2
430.	Cr.CLF	Cre17.g746247.t1.	460.	Vc.Atx2	D8TNR8
431.	Cr.Ashh1	A8I7A5	461.	Vc.Atx5	D8UBR4

Sl. No.	Name	Sequence id	Sl. No.	Name	Sequence id
462.	Vc.Atxr7	D8U8M3	484.	Vc.Rubisco.2	D8TNE3
463.	Vc.Orphan.1	D8TND4	485.	Vc.Rubisco.3	D8TQK7
464.	Vc.Orphan.2	D8TN92	486.	Vc.Rubisco.4	D8TTN7
465.	Vc.Orphan.3	D8TRT9	487.	Vc.Rubisco.5	D8U1T7
466.	Vc.Orphan.4	D8TS63	488.	Vc.Rubisco.6	D8UAM1
467.	Vc.Orphan.5	D8TSB7	489.	Cp.Ashh1a	Contig6713
468.	Vc.Orphan.6	D8TTI3	490.	Cp.Ashh1b	Contig8823
469.	Vc.Orphan.7	D8TUI1	491.	Cp.Atx2	Contig6840
470.	Vc.Orphan.8	D8TWF6	492.	Cp.Atxr	Contig56056
471.	Vc.Orphan.9	D8TZ02	493.	Cp.Atxr	Contig56056
472.	Vc.Orphan.10	D8TZ25	495.	Cp.Orphan.1	Contig25695
473.	Vc.Orphan.11	D8U0M3	496.	Cp.Orphan.2	Contig6860
474.	Vc.Orphan.12	D8U366	497.	Cp.Orphan.3	Contig43182
475.	Vc.Orphan.13	D8U5L8	498.	Cp.Orphan.4	Contig15015
476.	Vc.Orphan.14	D8U610	499.	Cp.Orphan.5	Contig39496
477.	Vc.Orphan.15	D8U6W9	500.	Cp.Orphan.6	Contig17150
478.	Vc.Orphan.16	D8UD47	501.	Cp.Orphan.7	Contig10195
479.	Vc.Orphan.17	D8UDH7	502.	Cp.Orphan.8	Contig41234
480.	Vc.Orphan.18	D8UI63	503.	Cp.Orphan.9	Contig43085
481.	Vc.Orphan.19	D8UIF3	504.	Cp.Orphan.10	Contig10998
482.	Vc.Orphan.20	D8UIW5	505.	Cp.Orphan.11	Contig7038
483.	Vc.Rubisco.1	D8TJD6	506.	Cp.Orphan.12	Contig11180

List of the SET domain containing proteins from species considered in the present study with their corresponding sequence id.
