# Supplementary material for: Phylogenetic relationship and domain organisation of SET domain proteins of Archaeplastida
Source: BMC Plant Biol. 2017 Dec 11;17:238. doi: 10.1186/s12870-017-1177-1 (PMC5725981; doi:10.1186/s12870-017-1177-1)
Supplement: Supplementary file 3 — Multiple sequence alignment of the 251 SET domain protein sequences from E(z), Ash, Trx and Su(var) of the 16 Archeplastida species. (PDF 74 kb) [file 12870_2017_1177_MOESM3_ESM.pdf]

```
                                        1        10        20        30
Pa.EZA1     ...........................QQRVLLGKSDVAGWGAFLKNPVN.KHEYLGEYT
Sm.CLF      .............................RVLLGRSDVAGWGAFLKTPVN.KHDYLGEYT
Pa.EZA2     ..............................VLLGRSDVSGWGAFLKNPVN.KNDYLGEYT
Os.CLF      .............................RVLLGRSDVSGWGAFLKNSVG.KHEYLGEYT
Nm.CLF      .............................RVLLAKSDVQGWGAFLQNSVG.RHDYLGEYT
Mp.CLF      .............................RVLLSRSDVAGWGAFLKSTVN.KHDYLGEYT
Pp.CLF      .............................RVLLGRSDVAGWGAFLKKTVA.KHEYLGEYT
Os.SWN      .............................RILLGKSDVAGWGAFIKNPVN.RNDYLGEYT
At.CLF      .......................................GWGAFLKNSVS.KHEYLGEYT
Mp.EZA      ............................KAVLLARSDVSGWGLFMKEAAI.THDYLGEYT
At.SWN      .......................................GWGAFLKNSVS.KNEYLGEYT
Mpu.CLF     ..............................ICLGVSAIAGWGAFLKDGAK.KNELLGEYT
Mr.CLF      ...................................SGVEGWGCFLKNGAR.KNELLGEYT
Kf.EZA      ............................KKILLGPSEVAGWGAFICGGAQ.AGELLGEYT
Ot.CLF      ............................EHVCLGKSGVAGWGAHVLHGAR.KDDFIGEYV
Ol.CLF      ............................EHICLGRSGVAGWGAFVLKGAR.KGEFIGEYV
Cv.CLF      ............................KRVLMGLSGIQGWGAFLQQDAQ.KDDFIGEYC
At.MEA      .......................................GWGAFTWDSLK.KNEYLGEYT
Mp.MEA      ...DSSTISASGRKQSKCQNMRILLNQKKSVLLARSDVIGWGLFLKSDAK.KDDYLGEYT
Pp.Atx6a    ............................TKLGVYKSGIHALGLYTTDFIA.EGEVVVEYV
Pp.Atx6b    ............................KKLAVYKSIIHALGLYTTDFIA.EREVVVEYV
Pp.Atxd     ............................KRLAVYKSGIHALGLYTTDFIA.EGEVVVEYV
Mp.Atxra    ............................RRLVVYKSHIHALGLYTAELIT.KDEMVVEYI
Os.Atx6     ............................KHLVVYKSSIHGLGLYTSEFIP.RGSMVVQYV
Pp.Atxr7a   ...............SDLLRFNMLKARKKQLKFQRSKIHDWGLVAAEPID.AEEFVIEYV
Pp.Atxr7b   ............................KHLKFQRSKIHDWGLLALESIE.AEDFVIEYV
Mp.Atxr7    ............................KRLKFQRSKIHEWGLIAVDPID.PEDFIIEYV
Pa.Atxr7    ............................KRLKFQRSKIHDWGLVALEPIE.AEDFVIEYV
Os.Atxr7    ............................KRLRFQRSKIHEWGLVALESID.AEDFVIEYV
Nm.Atxr7    ............................KKLKFQRSKIHDWGLVALEPIE.AGDLVIEYV
kf.Atxr7a   ............................KKLKFVRSKIHDWGIVTDEAIE.AEDFVIEYI
Sm.Atx5b    ............................KSLKFQRSKIHSWGVIALQSIE.PEDFVIEYI
Cp.Atxr     ............................KRLKFERSTIHEWGLFALEAIS.RDDMVIEYV
Sm.Atxr7    ...........................KKLLKFQRSKIHAWGVVAMEVIE.PEDFIVEYV
At.Atxr7    ........................................WGLVALEPIE.AEDFVIEYV
Pp.Atx2a    .............................RLVFGKSAIHGMGVFTKRVHY.ANDMIIEYA
Pp.Atx2b    .............................RLVFGKSAIHGMGVFTKQVHY.ANDMIIEYA
Pp.Atx2c    .............................RLAFGKSAIHGMGVFTKQVHY.ANDMIIEYA
Mp.Atx2     .............................RLTFGKSAIHGWGVFTKRAHC.AGDMVIEYA
Nm.Atxa     ............................ERLTFGKSAIHGWGVFTKKFHR.GGDMVIEYG
Nm.Atxb     ............................ERLTFGKSSIHGWGVFTKKPHR.GGDMVIEYG
Sm.Atx2b    ............................RRLTFGKSAIHGWGLFAKEPHG.AGDMVIEYA
Sm.Atx1     .............................RLTFGKSGIHGWGVFAKQIHK.AGDMMAEYA
Sm.Atx2c    .............................RLTFGKSGIHGWGVFAKQIHK.AGDMMAEYA
kf.Atxa     .............................RLAFGKSAIHGWGVFAKQRHA.QASFVTEYA
Cv.Atx      .............................RVTIGKSGIHGWGAFAKRRHA.EHDMVIEYV
Cr.Atxr1    ............................VRLAAGKSAIHGWGAFAKVPHK.RGDMLIEYA
Vc.Atx5     ..................................KSAIHGWGAFAKVPHK.RGDMLIEYA
At.Atx1     .......................................GFGIFAKLPHR.AGDMMIEYT
At.Atx2     .......................................GFGIFAKLPHR.AGDMVIEYT
Cr.Atxr     .........................................GLFAVEPIE.AGEFVIEYC
Vc.Atxr7    .........................................GLFAVEPIE.PSEFVIEYC
Cp.Atx2     .................................................K.SGSMVFEYV
Pp.Atx3     ..............................VCFGKSAIHGWGLFSRRAIQ.EGEMVIEYR
Mp.Atx5     .............................RVCFGKSGIHGWGLFARRAIR.EGETVLEYR
Sm.Atx2a    .............................RVCFGKSGIHGWGLFARRYIK.EGEMVVEYR
Sm.Atx5a    ..............................VCFGKSGIHGWGLFARRTIE.EGEIVVEYR
Os.Atx5     ..............................VCFGRSGIHRWGLFARRGIQ.EGEMVLEYR
Os.Atx4     ............................KRVSCGRSGIHGWGLFAAKKIQ.EGQMVIEYR
At.Atx3     ............................FRVCFGKSGIHGWGLFARKSIQ.EGEMIIEYR
At.Atxr3    .......................................GWGLFARKSIQ.EGEMIIEYR
At.Atx4     ......................................HGWGLFARRNIQ.EGEMVLEYR
At.Atx5     .......................................GWGLFARRNIQ.EGEMVLEYR
Nm.Atx5     ............................RRLCFGQSAIHGWGLMARRAIR.NGEIVGEYR
kf.Atxb     .............................RLSFGKSGIHGWGLIARKTIL.EGDYVVDYR
Pa.Atx5     ............................NRVCFGKSGVHGYGLFATKHIR.GGDMVIEYR
Cr.Atxr7    ............................ARITFGKSGIHGWGVFARVDIP.QDAVVTEFR
Vc.Atx2     .............................RITFGKSGIHGWGIFARTDIP.QDAIVTEFR
Ol.Atxr3    .............................RLTFAKSNIHGWGLVAKVFHK.AGSIVTQFK
Ot.Atx      ..................................KSNIHGWGLLAKTAHK.AGSIVTEFK
Mpu.Atx5    ............................ERFTFGKSNIHGWGLIAKKPVK.AGSMVIEFR
Mr.Atx5     ............................ERLTFGKSNIHGWGLIAKQFLK.AGSMVVEYV
Pa.Ashh1    ............................VKTKLFNTDGRGWGLLADQDIK.TGQFIIEYC
Os.Ashh1    ..................................KTEGRGWGLLADENIM.AGQFVMEYC
kf.Ashh4b   ..................................KTEGRGWGLYAQEDIK.AGQFVVEYC
At.Ashh1    .......................................GWGLVALEEIK.AGQFIMEYC
Mp.Ashh1    ......................................RGWGLAASQDLK.AGDFVMEYC
Os.Ashh2    ............................AKLGKFHTGKKGYGLQLKEDVS.EGRFLIEYV
Pp.Ashh2a   ............................ANVELFRCGKKGHGLRALENIP.RGTFIIEYV
Pp.Ashh2b   ............................ANVELFRCGKKGHGLRALENIP.RGTFIIEYV
Mp.Ashh3a   ............................APVATFRCGKKGFGLKVLKYTP.KDSFLIEYV
Nm.Ashh2a   ............................AKVRVVNCGRKGQGLQVLVDVP.KGGFIIEYV
Sm.Ashh2c   .....................................KKGFGLKALENIA.KGSFVIEYV
kf.Ashh1    ..............................LEVARCGSKGFGLRACEDIP.KQTFFIEYT
Cr.Ashh2    ............................ANLEIRRAGAKGFGLFALEDIK.AGQFIIEYI
Vc.Ashh1    ............................ANLEIRRAGAKGFGLFALEDIK.AGQFIIEYI
Cv.Ashh2    .............................KLDVKRAGAKGFGLFAAEDMK.AGQFLIEYL
Ol.Ashh4    ................................VRRTGKKGHGLFAAERVG.AGEFVLEYC
Ot.Ashh2    ............................KEVNVKRTGKKGHGLFAAQDVR.AGEFVMEYC
Mr.Ashh2b   ............................AKLDIQRTGRKGHGLFTKQALK.KGQFIIEYI
Mp.Ashh2a   .....................................KKGYGLRIMENAR.KDTFLIEYV
At.Ashh2    .......................................GYGLRLLEDVR.EGQFLIEYV
Os.Ashh3c   ............................RKTKLIKTEKCGNGVVAEEDIK.KGEFVIEYV
Nm.Ashh2b   ............................RKIRPVKTERCGWGVEALEDIK.AGDFIIEYV
Sm.Ashr3    ............................RRLRLKETENCGWGLFADENIE.RGDFLIEYI
Pp.Ashr3a   ..................................KTERCGWGLVADEDIK.AGSFLVEYV
Mp.Ashh3b   ..................................KTDHCGWGLKADEAIK.AGDFLVEYV
Os.Ashh3b   ............................KKIKAVKTKRCGWGAISLEPLE.KGDFIIEYV
Mp.Ashr3a   .............................RLKIVKTLQCGWGAESAELIR.KGEFIIEYI
Pp.Ashr3b   ............................KRLKVSKTAHCGWGAFTSVAIK.KDEFVIEYT
Nm.Ashr3    ............................KKMSIVKTRYCGWGVEAGEDVK.EGEFIIEYA
Os.Ashh3a   ......................................CGWGSRALEAIE.KDDFVIEFV
At.Ashr3    .......................................GWGVEAAESIN.KEDFIVEYI
```

Contd...

```
At.Ashh4      .......................................GYGIVADEDIN.SGEFIIEYV
At.Ashh3      .......................................GSGIVAEEEIE.AGEFIIEYV
kf.Ashh4a     ............................KKLELHKVRESEWTVGAGEDVA.AEDFVVEYV
Ol.Ashh2      ............................PKTKIIRTENRGWGLTLQEPVR.AGTFIVEYA
Ot.Ashh1      ............................PKTKIIRTENRGWGLMLQEPVT.AGTFIVEYT
Mr.Ashh2a     .......................................GWGLFLAEPVK.AGTFIVEYV
Cv.Ashh4      ...............................EVYLTPDKGWGVRAMAFIP.RGTFIVEYA
Cv.Ashh1      .................................KR.SGKGWGVYADEPLP.AGSLLGQYG
Pp.Suvr3a     ...........................ARVVVTRQ.RFTGWGLHAAQHIS.KGSFVCEYA
Sm.Suvr3      ...........................AELKVVRH.PTKGWSLHAAQDIK.PGAFICEYA
Kf.Suvr3      ...........................ARVKVVRH.RQKGWCLHAAEDIR.RGAFVCQYA
Os.Suvr3      ...........................VRLRVVRH.REKGWGLHAAEVLR.RGQFVCEYA
At.Suvr3      .......................................GWCLYADQLIK.QGQFICEYA
Mpu.Suvr3     .................................RR.NGYGFGVFANEKIE.RGTFVCEYA
Pp.Suvr3b     ............................PIELYKT.VNKGWAVRTLVAIPSRGRFVIEYV
Mp.Suvr5a     ............................SLELFKT.KDRGWGVRALEQIP.RGKFVVEYV
Mp.Suvr5c     ...................................................AGKFVVEYV
Nm.Suvr5b     ............................RLELFKT.PDRGWGVRTLERIP.RGRFVVEYV
Pa.Suvr3      ...........................VHLEVFKT.EERGWGVRTLDQIP.RGKFILEYV
Pp.Suvr5a     ...........................VKLEVFKS.RHKGWAVRSAQPIP.SGTFVCEYI
Nm.Suvr5a     ...........................VKLEVFRT.KHKGWAVRAMEAIP.RGTFVCEYI
Mp.Suvr5b     ...........................VKLEVFKT.HYKGWAVRAAQKII.RGTFVCEYI
Sm.Suvr5      ...........................VKLEVFKT.RHKGWAVRAAQNIS.RGTFVCEYL
Pa.Suvr5b     ...........................VKLEVFKT.WHKGWAVRAAESIS.RGTFICEYN
Pa.Suvr5a     ...........................VKIEVYKT.KHKGWAVRAAQAIS.RGTFICEYL
Pp.Suvr5b     ............................KLEVFIS.PHKGWGVRAAEAIS.RGTFVCEYV
Pp.Suvr5c     ............................KLEVFKS.RHKGWGVRAAEPIS.RGTFVCEYI
Os.Suvr5      ...........................VKLEVFRT.ENKGWAVRAAEPIP.QGTFVCEYI
At.Suvr5      .......................................GWGLRACEHIL.RGTFVCEYI
Pp.Suvr4b     ...........................ARLQVFWTG.GKGWGVRALDYLP.AGTFVCEYV
Pp.Suvr4a     ...........................RKLEVYMTPEGKGWGIRTLEDLP.AGAFVFEYV
Mp.Suvr4      ...........................RNLQIFFTQEGKGWGLRTLEQLP.PGAFVCEYV
Os.Suvr4a     ...........................RHLQVFLTPEKKGWGLRSTEKLP.RGAFVCEYV
Os.Suvr4b     ............................NLQVFFTGEGKGWGLRTLDELP.KGAFVCEYV
Sm.Suvr1      ...........................YKLEVFWTPTGKGWGVRTTEDLP.MGAFVCEYI
At.Suvr4      .......................................GWGLRTLQDLP.KGTFICEYI
Kf.Suvr4      ...........................VKLEVFWA.VGKGWAVRCLQDLP.AGTFVCEYV
At.Suvr1      .......................................GWGLRTLEKLP.KGAFICEYI
At.Suvr2      .......................................GWGLRTLEKLP.KGAFVCELA
Nm.Suvr4      ........................................WGVRSRERLL.KGTFVFEYI
Sm.Suvh5      ...........................KRLAIFKT.ESCGWGVRAAETIP.KDSFVCEYV
Sm.Suvh10     ............................KLAIFRT.EGKGLGLHAEEAIS.RGSFVCEYV
At.Suvh5      .......................................GWGVRSLESIP.IGSFICEYA
Mpu.Atxr1     .............................FRVDRSGV.HGMGVFTTVALP.GREMIMEYK
Os.Atx1       ...........................RRLAFGKSRI.HGFGVFAKVSHK.AGDMMIEYI
kf.Atxr3      ...........................ARVRVGASEI.HGKGLFAKTMIM.HREMVIEYV
Pp.Suvh4a     ...........................YRLEVYKTVS.KGWACRSWDFIP.AGAPICEYF
Sm.Suvh4      ...........................YRLEVYKTQH.KGWAVRSWDSIP.AGAPVCEYF
Os.Suvh4      ...........................YRLEVFKTAS.KGWGVRTWDTIL.PGAPICEYT
At.Suvh4      .......................................GWAVRSWEYIP.AGSPVCEYI
Pa.Suvh4      ...........................YHLEVFLTPN.KGWAVRSWDSIP.AGALVCEYT
Kf.Suvh4b     ............................KLEVFRTSN.KGWGLRSWNHIA.QGTVVCEYV
Pp.Suvh4b     ............................HLEIFRTMS.KGWGVRTLEFIP.SGSFLCELT
kf.Suvh4a     ............................RLEVFKTRN.RGFGVRSWDFIQ.VGSFICEYT
Mp.Suvr3      ...........................YDLEVFKTEG.KGWGVRSSNFIP.QGSFICEYI
Sm.Suvh6      ...........................RKLEVFKTEN.RGWGVRSWEAIP.FGSFICEYV
Nm.Suvh4      ............................RLEVFKTEQ.KGWGLRSWNSIP.AGSFVCEYA
Pa.Suvh5b     .............................LEVFKTAK.RGWGVRSLTSIS.SGSFICEYT
Pa.Suvh5a     ...........................YNLEVFKTDK.RGWGVRSSDSIP.SGSFICEYT
Os.Suvh5c     ...........................IHLEVFRTAN.KGWGVRSLRSIS.SGSFICEYV
Os.Suv5d      ...........................FRLQVFKTKL.MGWGVRTLDFIP.SGSFVCEYI
Os.Suvh5a     ...........................FRLQVFKTKL.MGWGVRTLDFIP.SGSFVCEYI
Os.Suvh5b     ...........................IPLEIFKTGN.KGWGVRSLSSIS.SGSFVCEYA
At.Suvh6      .......................................GWGVRCLKSIP.IGSFICEYV
Pa.Suvh7b     .............................LEVFKTNG.KGWGLRSWDPIP.AGSFICEYT
Pa.Suvh7c     ...........................FHLEVFRKDN.GGWGVRSWDPIP.AGTFICEYV
Pa.Suvh7a     .............................LEVFRTSD.KRWGVRSWDPIA.AGTFISEYT
Os.Suvh7      ...........................IHLEVFRTTN.RGWGLRSWDPIR.AGSFICEYV
Os.Suvh8      ...........................FHFEVFRTAN.RGWGLRCWDPIR.AGAFICEYT
Os.Suvh11     ............................NFEVFWTGD.RGWGLRSWDPIR.AGTFICEYA
Os.Suvh1      ...........................IHFEVFKTGD.RGWGLRSWDPIR.AGTFICEYA
Cv.Suvh4      ............................PLEIFRTRG.KGWGVRCARDIA.GGSYVCSYE
AtSuvh8       ......................................CGWGLRSWDPIR.AGTFICEFT
At.Suvh7      .......................................GWGLRSWDPIR.AGTFICEFA
At.Suvh3      .......................................GWGLRSWDSLR.AGSFICEYA
At.Suvh1      .......................................GWGLRSWDAIR.AGSFICIYV
Pa.Suvh2c     ............................KIEIFRTPR.KDWGVRTLDFIQ.AGAFIAEYT
Pa.Suvh2b     ............................ELEIFRTPY.KDWGVRTLEFIQ.AGAFIAEYT
Pa.Suvh2a     .............................LEIFRTSN.NEWGVRTTQCIE.KGAFIAEYT
Os.Suvh2      ...........................NRLEVFRSRE.TGWGVRSLDLIK.AGTFICEFS
Os.Suvh9      ............................RLEVFRSKE.TGWGVRTLDLIQ.PGAFICEYA
AtSuvh2       .......................................GWGVRTLDLIE.AGAFICEYA
At.Suvh9      .......................................GWGVRSLDVLH.AGAFICEYA
Cv.Suvh2      ...........................YRLEIFKTRNGRGWGVRSLDTIP.QFGFVVAYV
Mpu.Suvh4     ...........................FPLHVYQDDNGKGFCVRAKQKIP.KGGLICEYV
Mpu.Suvr5     ........................................WGLRCRAEIS.SGAFVCEYA
Cr.CLF        .......................................GWGLFAAQYVA.RNGFLGEYT
Pa.Suvh10a    ...........................VRVAVVRHPR.KGWGLHSLEPIT.RGAFVCEYA
Cp.Ashr2a     ...........................AETEVFNTGS.KGWGIRALEDIP.KGTFV..YS
Cp.Ashr2b     ...........................AKVEVFHTRE.RGWGARAAEAIP.AGGFVVEYA
Mp.Ashh2b     .................................KTDH.CGWGLKADEAIK.AGDFLVEYV
Pa.Suvr4      ............................KLQVFFTSEGKGWGVRTLEDIP.RGAFVCEYV
Cr.Ashh1      ...........................PKTEVFRTEN.RGWGVRAMEPVT.KVGLYIDAR
Cr.Suva       ...........................LPLEVFMTES.KGWGVRCREEVP.AGAFVCCYV
Os.Suvh10     ...........................VRLRVVRHRE.KGWGLHAAEVLR.RGQFVCEYA
Pa.Suvh10b    .............................LEVFKTNG.KGWGMWSWDPIP.ANRFICEYT
Pa.Atx6       .FCEECGLGDCENEMLLCDKCDRGYHMYCLRPIVARVPAGRWFCPNCKADPIKEF.....
Cr.Suvb       LVIDARTTGNVGRFINHSCDGNLTIQAVFAGVYRSTLLYHVGLYACRNIPQLEELSYNYG
Vr.Suv        LVLDARSMGNVGRFINHSCDGNLTIQAVFAGSHRNTFLYHVGLYACTDIPPMTELTYNYG
At.Suvh10     ....AKKSGNVARFMNHSCSPNVFWQSI.AREQNGLWCLYIGFFAMKHIPPLTELRYDYG
Ot.Ashh4      ............................................................
Os.Atxr3      ............................................................
Mpu.Atxr5     ............................................................
```

Contd...

```
Pp.Atxr5        ............................................................
Nm.Atxr5        ............................................................
Pa.Atxr6a       ............................................................
Pa.Atxr6b       ............................................................
At.Atxr6        ............................................................
Os.Atxr6        ............................................................
At.Atxr5        ............................................................
Os.Atxr5        ............................................................
Cv.Ashr3b       ........................................WGVFATRPLP.AGTLLGELC
Sm.Ashh1        ............................................................
At.Atxr1        ............................................................
Cr.Suvc         .............................RVRLSWVPGKGWAAFAAEPLP.AGAFVCRYE
Mpu.Suvh10      .............................RMEVFRTEGKGWGVRSWDPIK.AGEFVCEFT
At.Ashr2        .........................................................TSF
Sm.Ashr2        ...........................................................M
Nm.Ashr2        ...........................................RRERRVRAYGIYPVASL
Cv.Ashr2b       .........................................................ASL
Vc.Ashr2        .........................................................ASL
Mr.Ashr2        ..........................................................SR
Mpu.Ashr2       ..........................................................SR
Ol.Atxr4        ........................................................YVSL
At.Ashr1        ..........................................................SI
Mr.Ashr1b       ............................................................
Os.Atxr1        ...........................................................F
Cr.Ashr1        ...................................NCHTV..CDEELRPLGTALYPSGAL
Cv.Ashr1        ................................FGCNSHTI..SDDELQPLAVGIFPLGAM
Cr.Ashr2        ...................................................TLMVPYADL
Ot.Atxr4a       ..................................................GVVLLGSASN
Sm.Atxr4        ..........................................................SM
At.Atxr4        ..........................................................SF
Os.Atxr4        ..............ARIRINAFRIELVASSYENLLSSAVA..SVSCDAAVGNAVYMLPSF
Cv.Ashr2a       ............................................................
Mpu.Ashr1       ............................................................
Ot.Atxr4b       ............................................................
Mr.Ashh1        MIKFAGQVTDSYAEERTLDPTLALEEWRW.AMRAVHS.RTFRVEDERGVRPTRRALIAAA
Sm.Suvh2        .........KNLRFSRALKKNLRFSGLRAKARRRSHSQRQFRMQICGEVLEDKGSPSTYK
Cv.Atxr         ...............GVLVRASVAEARERRYRQQGRDCYLFNLDDQHVLDATRAGAISKF
Pa.Atx1         .......................................LFKISEEVVVDATEKGNVARL
Pa.Atx4         .........................................................ARL
Mp.Atxr2        ............................................................
Nm.Atxr2        ............................................................
Os.Atxr2        ...............FIHIDDLPDDEKEEAEKVTRPFLDALGEDYAAPCEGTAFFPLQSC
Sm.atxr2        ............................................................
Ol.Atxrb        ............................................................
At.Atxr2        ............................................................
consensus>50    .......................................gwg....e.i...g.fv.ey.
```

Contd...

```
                    40        50                  60
Pa.EZA1    GELISHKEADKR....GKIYDRE.........DSSFL...........FNLNDQ.....
Sm.CLF     GELISHREADKR....GKIYDRE.........NSSFL...........FNLNDQ.....
Pa.EZA2    GELISHREADKR....GKIYDRE.........DSSFL...........FNLNDQ.....
Os.CLF     GELISHKEADKR....GKIYDRE.........NSSFL...........FNLNNE.....
Nm.CLF     GELISHTEADKR....GKIYDRV.........NSSFL...........FNLNDQ.....
Mp.CLF     GELISHREADKR....GKIYDRE.........NSSFL...........FNLNDQ.....
Pp.CLF     GELISHREADKR....GKIYDRE.........NSSFL...........FNLNDQ.....
Os.SWN     GELISHREADKR....GKIYDRA.........NSSFL...........FDLNEQ.....
At.CLF     GELISHKEADKR....GKIYDRE.........NCSFL...........FNLNDQ.....
Mp.EZA     GELVTQQEADKR....GKIYDLV.........NLSFL...........FDLNET.....
At.SWN     GELISHHEADKR....GKIYDRA.........NSSFL...........FDLNDQ.....
Mpu.CLF    GELITQVEADRR....GKIYDRV.........NCSFL...........FNLNDQ.....
Mr.CLF     GELISQTEADRR....GKIYDKL.........NSSFL...........FNLNDQ.....
Kf.EZA     GELVTDQEAEQR....GRVYDAN.........NKSFL...........FQLNDQ.....
Ot.CLF     GELVTQDEADRR....GMVYDRN.........NCSYL...........FDLNSE.....
Ol.CLF     GELVTQDEAERR....GTVYDVN.........NCSYL...........FNLNSE.....
Cv.CLF     GELINHEEADRR....GTVYDRD.........DNSYL...........LE........
At.MEA     GELITHDEANER....GRIEDRI.........GSSYL...........FTLNDQ.....
Mp.MEA     GELISQREADKR....GKIYDIV.........NLSFL...........FDLNDK.....
Pp.Atx6a   GEIVGSRVADKR....EAEYHSG..KRL.QYQGACYL..........FRIDTEQII..
Pp.Atx6b   GEIVGHRVADKR....EVEYHSR..KRL.QYQGACYL..........FRIDTEQII..
Pp.Atxd    GEIVGSRVADKR....EAEYHSG..KRL.QYQGACYL..........FRIDTEQII..
Mp.Atxra   GEIVGLRVADKR....EADYHSG..KLLISHQGACYL..........FRIDKERII..
Os.Atx6    GEIVGQCVADKR....EIEYQSG..KRQ.QYKSACYF..........FKIGKEHII..
Pp.Atxr7a  GEVIRNRVTDIR....EKRYEA.......IGIGSSYM..........FRVDDEHTLNT
Pp.Atxr7b  GEIIRRQVSNFR....ERQYEI.......MGIGSSYL..........FRVDDELVV..
Mp.Atxr7   GELIRTKISDLR....EHLYES.......RGIGSSYL..........FRIDDEFVV..
Pa.Atxr7   GELIRPKISDIR....ERQYEK.......MGIGSSYL..........FRVDHEYVV..
Os.Atxr7   GELIRRQVSDIR....EDQYEK.......SGIGSSYL..........FRLDDDYVV..
Nm.Atxr7   GELIRRKVSDIR....EKKYEA.......MGIGSSYL..........FRIDDDYVV..
kf.Atxr7a  GQVIRRPVSELR....EQRYEK.......LGIGSSYL..........FRIDDDNVI..
Sm.Atx5b   GELVRSKVADLR....ERRYEK.......MGIDSSYL..........FRVDAENVV..
Cp.Atxr    GEVIRQKVADER....EKAYEK.......VGIGSSYL..........FRIDEDSIV..
Sm.Atxr7   GEVLRPKVADVR....EVRYLR.......QGLGSSYF..........FRVGDGFVI..
At.Atxr7   GELIRSSISEIR....ERQYEK.......MGIGSSYL..........FRLDDGYVL..
Pp.Atx2a   GEVVRPVVADSR....ERRHYDS......LVGAGTYM..........FRIDDERVV..
Pp.Atx2b   GEVVRPVIADIR....ERRFYDS......LVGAGTYM..........FRIDDERVV..
Pp.Atx2c   GEVVRPVIADIR....ERRCYDS......LVGAGTYM..........FRIDDERVV..
Mp.Atx2    GEIVRPIIADIR....ERRSYDS......LVGAGTYM..........FRIDDERVV..
Nm.Atxa    GELVRPVVADMR....EKRCYDS......LVGAGTYM..........FRVDEERVV..
Nm.Atxb    GELVRPVVADMR....EKRCYDL......LVGAGTYM..........FRVDEDRVV..
Sm.Atx2b   GEIIRPTVADVR....EKRCYNS......LVGAGTYM..........FCIDNERVV..
Sm.Atx1    GEIVRSNIADIR....ERRHYDS......LVGAGTYM..........FRIDDERVV..
Sm.Atx2c   GEIVRSNIADIR....ERRHYDS......LVGAGTYM..........FRIDDERVV..
kf.Atxa    GEVVRQAVADAR....EKNLYNS......IVGAGTYM..........FRIDDQRVV..
Cv.Atx     GELVRPSVSDLR....EARCYDD......MVGAGTYV..........FRLNKALCV..
Cr.Atxr1   GELIRPVVSDVR....EKRMYND......LVGCGTYI..........FSLNGQQHI..
Vc.Atx5    GELIRPSVSDVR....EKRMYNK......LVGCGTYI..........FTLNDDQHI..
At.Atx1    GELVRPSIADKR....EQLIYNS......MVGAGTYM..........FRIDDERVI..
At.Atx2    GELVRPPIADKR....EHLIYNS......MVGAGTYM..........FRIDNERVI..
Cr.Atxr    GVRLRKPLDDVR....QRQYDA........AGYMDYM..........FAVDGAWVV..
Vc.Atxr7   GQLIRKPLDDVR....QRQYDA........AGYADYM..........FAVDDNWVV..
Cp.Atx2    GEVIQPPVADLR....EPKYRK........ERLGDYF..........FRISETEII..
Pp.Atx3    GERVRGSVADLR....EIRYHKEGK.......DC.YL..........FKINEE..I..
Mp.Atx5    GERVRRIIADQR....EKRYTKEGK.......DC.YL..........FKVSDDDDL..
Sm.Atx2a   GERIRRSVADLR....EKRYCLEGK.......HC.YL..........FKISEE..V..
Sm.Atx5a   GEQVRRSVADLR....EKRYRDQGK.......DC.YL..........FKISEE..I..
Os.Atx5    GEQVRRSVADLR....EEQYRVQGK.......DC.YL..........FKISEE..V..
Os.Atx4    GDQVRRSVADLR....EARYHREKK.......DC.YL..........FKISED..V..
At.Atx3    GVKVRRSVADLR....EANYRSQGK.......DC.YL..........FKISEE..I..
At.Atxr3   GVKVRRSVADLR....EANYRSQGK.......DC.YL..........FKISEE..I..
At.Atx4    GEQVRGSIADLR....EARYRRVGK.......DC.YL..........FKISEE..V..
At.Atx5    GEQVRGIIADLR....EARYRREGK.......DC.YL..........FKISEE..V..
Nm.Atx5    GVLVGGVVANMR....EERYQQQGK.......HC.YL..........FKIDEE..H..
kf.Atxb    GTLVRPSVANLR....EKYYRRIGK.......DC.YL..........FKVDDQ..H..
Pa.Atx5    GDIIRDSVADVR....EKRHHSHFK.......HSTYF..........FTIDKE..T..
Cr.Atxr7   GEVVRPVLAEMR....ERRYRAAGRAWACAGKDC.YL..........FHVSRE..L..
Vc.Atx2    GEAVRPVVADLR....ERRYKAQGR.......DC.FL..........FHMNGE..V..
Ol.Atxr3   GETCRSTVADLR....ETFYEDNGV.......DC.YL..........LKQDDD..T..
Ot.Atx     GETCRSTVADMR....ETAYEEEGV.......DC.YL..........LKQDDD..T..
Mpu.Atx5   GEIVKPHVADLR....EKAYDDANI.......DC.YL..........LKADEK..T..
Mr.Atx5    GERLRPSVADLR....EKVYERTGH.......DV.YL..........LAADDK..T..
Pa.Ashh1   GEVISREEARRR....SQAYEAA.......GLKDAFI..........ISLNKSELI..
Os.Ashh1   GEVISWKEAKRR....SQAYENQ.......GLTDAYI..........IYLNADESI..
kf.Ashh4b  GEIINDREAVKR....SEDYRRE.......GLRDHYM..........LILSGSEVI..
At.Ashh1   GEVISWKEAKKR....AQTYETH.......GVKDAYI..........ISLNASEAI..
Mp.Ashh1   GEVVPVKEALQR....AKAYETS.......GTTNWFI..........LNLNGGEYI..
Os.Ashh2   GEVLDITAYESR....QRYYASK.......GQKHFYF..........MALNGGEVI..
Pp.Ashh2a  GEVLDMPSFEAR....QKEYSMN.......SQKHFYF..........MTLSANEII..
Pp.Ashh2b  GEVLDMPSFEAR....QKEYSMN.......SQKHFYF..........MTLSANEII..
Mp.Ashh3a  GEVLDVGAFEER....QQDYARN.......GQKHFYF..........MTLSSTEVI..
Nm.Ashh2a  GEVLDEETFEAR....QWEYRFE.......DQRHLYF..........MTLNATEVI..
Sm.Ashh2c  GEVLDSRSFELR....QKEYARQ.......RQKHFYF..........MTLNSSEVI..
kf.Ashh1   GEVLDEATFEGR....LRQYARE.......GQRHMYF..........MTIGNGEYI..
Cr.Ashh2   GEVLEEDEYQRR....KEYYMSV.......GQRHYYF..........MNIGNGEVI..
Vc.Ashh1   GEVLEEDEYQRR....KEYYMSV.......GQRHYYF..........MNIGNGEVI..
Cv.Ashh2   GEVLEEEEYHRRQGAAWKEYFIET.......GQRHYYF..........MNVGNGEVI..
Ol.Ashh4   GEVLHEEAYKER....KRRYQDE.......GRSHYYF..........MTLSSSETI..
Ot.Ashh2   GEVLHEDAYAER....KQRYHDE.......GRSHYYF..........MTLSSSETI..
Mr.Ashh2b  GEVLHEDEYRSR....KARYDDE.......GRRHYYF..........MTLSSSETI..
Mp.Ashh2a  GEVLDKRVRAAR....EQDYIRQ.......GREHLYF..........MSLSRTQTI..
At.Ashh2   GEVLDMQSYETR....QKEYAFK.......GQKHFYF..........MTLNGNEVI..
Os.Ashh3c  GEVIDDRTCEQRL....WKMKRQ.......GDTNFYL..........CEVSSNMVI..
Nm.Ashh2b  GEVIDDEICEKRL....WAMKAM.......GETNFYL..........CELNKDMVV..
Sm.Ashr3   GEVIDDKTCEERL....WDLKER.......GENNFYL..........CEVGHDKVI..
Pp.Ashr3a  GEVIDDQTCEERL....WAMKKQ.......GEMNFYM..........CEISREMVI..
Mp.Ashh3b  GEVIDDETCEKRL....WAMKEQ.......GESNFYM..........CEISREMVI..
Os.Ashh3b  GEVINDATCEQRL....WDMKRR.......GDKNFYM..........CEISKDFTI..
Mp.Ashr3a  GEVINDAMCENRL....WAMKGK.......GISKFYM..........CEIMKDFII..
Pp.Ashr3b  GEVIDDAMCEKRL....WEMKGRR.......SICNFYM..........CEIAKDFII..
Nm.Ashr3   GEVIDDALCEKRL....WDLRAR.......GAENFYM..........CEISKNLVI..
Os.Ashh3a  GEVIDDETCEERL....EDMRRR.......GDKNFYM..........CKVKKDFVI..
At.Ashr3   GEVISDAQCEQRL....WDMKHK.......GMKDFYM..........CEIQKDFTI..
```

Contd...

```
At.Ashh4     GEVIDDKICEERL....WKLNHK........VETNFYL...........CQINWNMVI..
At.Ashh3     GEVIDDKTCEERL....WKMKHR........GETNFYL...........CEITRDMVI..
kf.Ashh4a    GEVLDSALCHQRI....SALERR........GAHNRHL...........CQLDDSFVL..
Ol.Ashh2     GEILDEHECAERL....WYDKQS........GEENFYL...........MEISANYVI..
Ot.Ashh1     GEILNEADVANRL....WLDKQE........GEENFYL...........MEISTNYVI..
Mr.Ashh2a    GEILDEHTTEKRL....WEDKKR........GEDNFYL...........MEVMPNQCI..
Cv.Ashh4     GEVIDDKECSRRA....EDAKAR........NEPHFYM...........MEMAPGLII..
Cv.Ashh1     GEYIS..NAEAQRRLKEYD...............TSGGGH..ALLVLREWLPSGTAA...
Pp.Suvr3a    GELLT..TVQSRERQSLYD.............AGNTSCGS..ALLVVREYMPSGEAC...
Sm.Suvr3     GELLT..TKEARKRHQTYD.............QSPRA..T..SLLVVREHLPKGDAC...
Kf.Suvr3     GELLT..NAEANRRHLIYDCPE.........YHSSGRPPP..ALLVVREHLPSKTAS...
Os.Suvr3     GELLT..TEEARRRQGLYD..E.........LASVGKLSP..ALIVIREHLPSGKAC...
At.Suvr3     GELLT..TDEARRRQNIYD..K.........LRSTQSFAS..ALLVVREHLPSGQAC...
Mpu.Suvr3    GEVID..AAAAAKRLRIVDENKSSNYVLVSRMGTAGGGDG..GGGDDDDEGDEAANE...
Pp.Suvr3b    GEMLT..QDQAQRYGSYY...............DALKRSY..LYDLDYPESKKT......
Mp.Suvr5a    GDVLT..QAEADERGEKY...............DQNQLSY..LFNLDHPGVEHS......
Mp.Suvr5c    GDVLT..QAEADERGEKY...............DQNQLSY..LFNLDHPGVEHS......
Nm.Suvr5b    GELLT..QAEAEERAHEY...............DASRVSC..LFNIDPPDLAPD......
Pa.Suvr3     GEMLT..EGEVSKRGLVY...............PEYNSSY..LFNVDHPAVPQE......
Pp.Suvr5a    GEVVN..DREANQRGVRY...............DQDGCSY..LYDIDAHLDMSISRAGA.
Nm.Suvr5a    GEVVD..DREANRRGERY...............DQVGCSY..LYDIDVHLDPPAFRNRV.
Mp.Suvr5b    GEVLH..DQEANKRGERY...............DQVGCSY..LYDIDAHLDISGTGRRS.
Sm.Suvr5     GEVLN..DQEANRRGERY...............DQVGCSY..LYDIDVHLNTGGRSRRGP
Pa.Suvr5b    GEVLS..DQEANKRGERY...............DKGGCSY..LYDIDPHIATNDFMEET.
Pa.Suvr5a    GEVLN..DQEANKRGERY...............DNEGCSY..LYDIDAHIDTSDLTDGV.
Pp.Suvr5b    GEVLN..DSEANKRGKRAL.VLDDLFVSLRELTKRTSWNY..LYNIDAHLDVVGVKSIS.
Pp.Suvr5c    GEVLN..DKEANERGKREPHEYTDTFVCSRY..DQVGCSY..LYNIDAHLDVIGSKSVS.
Os.Suvr5     GEVLKMKDDGAIRHVERE...............AKSGSSY..LFEITSQIDRERVQTTGT
At.Suvr5     GEVLD..QQEANKRRNQY...............GNGDCSY..ILDIDANINDIGRLMEEE
Pp.Suvr4b    GEILT..NTEMWFRNN..E..............SHRSAKH..HFSLNLDADWCSE.....
Pp.Suvr4a    GEILT..NTEMWERNNEII..............RNGEGRH..TYPVALDGDWGSE.....
Mp.Suvr4     GEIMT..NIELDKRNHAQK..............GESVGAH..TYPILLDGDWCSE.....
Os.Suvr4a    GEILT..NIELYDRT..IQ..............KTGKAKH..TYPLLLDADWGTE.....
Os.Suvr4b    GEVLT..STELHERT..LQ..............NMNNGRH..TYPVLLDADWGSE.....
Sm.Suvr1     GEILT..NTELDERNE.ER..............FLKQSRH..FYPIYLDSDVCTE.....
At.Suvr4     GEILT..NTELYDRN...V..............RSSSERH..TYPVTLDADWGSE.....
Kf.Suvr4     GEILT..TDELDTRN...R..............ALGNEKK..HFPTELNACPVTE.....
At.Suvr1     GEILT..IPELYQRS....................FEDKP..TLPVILDAHWGSE.....
At.Suvr2     GEILT..IPELFQR.....................ISDRP..TSPVILDAYWGSE.....
Nm.Suvr4     GEIVT..NLEQMERNK..N..............FEMDDVE..GFPVQLDAHYEIEHNFFD
Sm.Suvh5     GEVLD............................FSSSARH..DYQFQMPGDARFPA....
Sm.Suvh10    GEVLE............................DKGSPS...TYKFAIGPE.........
At.Suvh5     GELLE............................DKQAESL..TGKDEYLFDLGDED....
Mpu.Atxr1    GEIVRRPIADRREAAERAAIARRAKDAADSAARRGDPPPPPLSE......ADLAAASTYM
Os.Atx1      GELVRPPISDIRERRIYNSLVPEGYMWKTLVKLIGLFMEFNLKALMGVIITTFLGAGTYM
kf.Atxr3     GELIRPKISDLHEKQYRAQKMGISDMREKQYRAQKMGCYF...............FGFPP
Pp.Suvh4a    GTLR.RNDENLESMLDNSYIFELDLLQTM...QGMEGRQ......KRFGDVMPELSDEDD
Sm.Suvh4     GKVI.KSDS.LD.VKSDVYLFDLDCIQTM...RGVDGRQF...LDYQNGKVSCESRDAED
Os.Suvh4     GVLR.RTEE.VDGLLQNNYIFDIDCLQTM...KGLDGRE.....KRAGSDMHLPSLHAEN
At.Suvh4     GVVR.RTAD.VDTISDNEYIFEIDCQQTM...QGLGGRQ....RRLRDVAVPMNNGVSQS
Pa.Suvh4     GILM.RTDE.LDSVLENNFIFEIDCVQTM...KGIDGRQ......RRLGDVSKH......
Kf.Suvh4b    GEQL.RDSE..VGNRDDMYLFNLDTAKTS...LEV..........RHDRAKKLNLVDETN
Pp.Suvh4b    GELL.TATA.AADRENDEYLFNLDFHKNA...RGR...............GKPSKSKRQA
kf.Suvh4a    GQIL.TDAQ.AHECDDDSYLFNID..PTT...DGF...............VGEEFGAEAS
Mp.Suvr3     GEVV.HTSEAEQRVTSDDYLFDLNVPQ....SQSKRFGDV...SG..LVEDVSDSGDESE
Sm.Suvh6     GELL.SNEEAERRVGQDEYIFDIDCIK....GSRSRGVDI...SS..FFEE.KDGGEICE
Nm.Suvh4     GRVL.KDTDGE.KVDCDEYLFDLDLIRIMKYSAKSRWGDV...SD..IDTTDEHYASKLS
Pa.Suvh5b    GELL.SDMEAEQRTGNDEYLFDIGSG..SN..DQSLWDGL........AGLVNELPSHSI
Pa.Suvh5a    GKLL.SDTEAEQCVGNDEYLFDIGRNCTSK..EQSLWTRL........STLMPEIEADAD
Os.Suvh5c    GILL.TDKEADKRT.NDEYLFDISHNCDDE..DCSK.GRP........STISSLNSSGGC
Os.Suv5d     GEVL.EDEEAQKRT.TDEYLFAIGHN..YY..DEALWEGL........SRSIPSLQKGPD
Os.Suvh5a    GEVL.EDEEAQKRS.TDEYLFAIGHN..YY..DEALWEGL........SRSIPSLQKGPD
Os.Suvh5b    GEVL.QE.NGDEHVETDEYLFDIGHHYHDEVWEDPKFEGI...LGLESSTSKTTEDTEGS
At.Suvh6     GELL.EDSEAERRIGNDEYLFDIGNRY.....DNSLAQGM.......SELMLGTQAGRSM
Pa.Suvh7b    GEVL.SDSDSEQEDEDNDYLLRANHIQESRTDLDSISDVL.........SEQNQGTYTKP
Pa.Suvh7c    GEIL.LDEEIDQQFEENQYVLNANRSQENLTDWGNVSDML.........PEKKQDGASPT
Pa.Suvh7a    GQVVPVAEQAAICHSDGEYVLNLRHAQK.RTEWGSVSDIL.........CEEEQQQQAGS
Os.Suvh7     GEVV.DDTKVNLD.GEDDYLFRTVCPGEKTLKWNYGPELI.........GEHSINISADT
Os.Suvh8     GEVI.DELKVNLDDSEDDYIFQTVCPGEKTLKFNFGPELI.........GEESTYVSADE
Os.Suvh11    GEVI.DETKMDIDVEEDKYTFRASCPGNKALSWNLGEELL.........EEKSTAVITKN
Os.Suvh1     GEVI.DRNSI...IGEDDYIFETPS.SEQNLRWNYAPELL.........GEPSLSDSSET
Cv.Suvh4     GVLL..AHKEAESRRNDAYLFDLEHFFLMHRDPSMKGQRR.......QRLPPLPADVRPG
AtSuvh8      GVSK.TKEEVE...EDDDYLFDTSRIYHSFRWNYEPE............LLCEDACEQVS
At.Suvh7     GLRK.TKEEVE...EDDDYLFDTSKIYQRFRWNYEPE............LLLEDSWEQVS
At.Suvh3     GEVK.DNGNLRGNQEEDAYVFDTSRVFNSFKWNYEPE............LVDEDPSTEVP
At.Suvh1     GEAK.DKSKVQQTMANDDYTFDTTNVYNPFKWNYEPG............LADEDACEEMS
Pa.Suvh2c    GNLC...L..SRNTSMHKFIFPISSIPMEGNWGDLRG............VVDDELVLASP
Pa.Suvh2b    GDLI...VDPSPSNSMNKFIFPISSISMEGNWGDLRG............LVDDDVVSVSP
Pa.Suvh2a    GDLI...VDRSPNNSNNKFIFPITSSSMQG.........................YGASS
Os.Suvh2     GIVLTHQQSEIMAA.....NGDCLVRPSRFPP.RWLDWG........DVSDVYPEYVAPN
Os.Suvh9     GDVLSLDSHSGDAPLPPMEDGSSIIDPTKFPE.RWREWG........DASVVYPDRV.PH
AtSuvh2      GVVVTRLQAEILSM.....NGDVMVYPGRFTD.QWRNWG........DLSQVYPDFVRPN
At.Suvh9     GVALTREQANILTM.....NGDTLVYPARFSSARWEDWG........DLSQVLADFERPS
Cv.Suvh2     GEVYDAEEHEHLVRTVEEQDAEYTFDM....APR..............PDTNWDGTEKV
Mpu.Suvh4    GEIITQKEAAKRERNYSQLGLFYLHDVHTNYRSK...............RKSPIDRSNKI
Mpu.Suvr5    GEVLTESEAAARCVNSGDDDYIFSL...........................DHFDVAYQ
Cr.CLF       GDLITQDEANRRGSVYDH.MNNRGNKLRCANHSSEPVAKAKVLLVDGESRIAIFADKP..
Pa.Suvh10a   GELLTTVEARERQRHYDDAIVNKHNSCSALLVIREHLPSGSIL.....ARLCFFASRD..
Cp.Ashr2a    GEICDADEFRFRAIDYQK...QADVHFYFMLTEGDEKIDASRK.....SAEGRFANHS..
Cp.Ashr2b    GEIIDEEEMARRAEESEE...GAA..YYFMRLNGPYIIDAPR......ATYARFLNHS..
Mp.Ashh2b    GEVIDDETCEKRLWAMKE...QGESNFYMCEISREMVIDATFK.....GNLSRFINHS..
Pa.Suvr4     GEILTNTELYNRNNE.RM...GNEKHTYPVLLDADWCSEGVLK.....DEDALCLDAT..
Cr.Ashh1     RKGNTARLINSSCEP......NCETQKWHDAATGE.............VRVGIFALRD..
Cr.Suva      GQLITDAMAEVRKGVDHY...LFDLDFFAHIYA...........................
Os.Suvh10    GELLTTEEARRRQGLYD...........................................
Pa.Suvh10b   GEVLSDSDSEQENED.............................................
Pa.Atx6      ............................................................
Cr.Suvb      Y...........................................................
Vr.Suv       Y...........................................................
At.Suvh10    ............................................................
Ot.Ashh4     ............................................EFYAVHRKGFGVVCKE
Os.Atxr3     ..............................................YVAYRKGLGVVCNK
Mpu.Atxr5    ............................................GHGLVCVRADGIPKGA
```

Contd...

```
Pp.Atxr5       ............................................................
Nm.Atxr5       ...................................................APLLVRHDE
Pa.Atxr6a      ...................................................PPLSVVFDP
Pa.Atxr6b      ...........................................................P
At.Atxr6       ............................................................
Os.Atxr6       ............................................................
At.Atxr5       ............................................................
Os.Atxr5       ............................................................
Cv.Ashr3b      G.MVCTGEEVDDAVAAEPRGVGGLRADQIKALWMTAGGEEGGRQPPRNGWARELVIDGSE
Sm.Ashh1       ....GRGLFATKALKAGDLIFATRPLAME.............DYFNDLSLKQDILNKASD
At.Atxr1       ....GRGLFATKNIVAGTLVLVTKAVAIERGILGNGECGEKAQLIMWKNFVEEVTESVRK
Cr.Suvc        GELLRSGEAERRLRHVYDCSSSSRYGSSSGSSSRSSSGSGSTGAGFYKE.EGEEVER.KE
Mpu.Suvh10     GEMLTHSEAEKRGEHEHEDAYEGAGEYDEYLFGLNPSHPEPLAALLKGEYDDEDVKKFKA
At.Ashr2       FNHDCLPNACRFDYVDSASDG.........NTDIIIRMIHDVPEGREVCLSY........
Sm.Ashr2       LNHDCLPNACRFEYLDKPGAS.........NTDIYIRLLHDVPQGSEICVSYFPVNWN..
Nm.Ashr2       INHDCLPNVARFDYVDQSNGD.......GHNKDIILRAMHDIPKGTEITMSYFPINW...
Cv.Ashr2b      INHECLPNVARFDRFDASSAPASSAPHPGANTAVEFRALHDIPAGEELTQSY........
Vc.Ashr2       INHECLPNVARFDDFDSDLPD.........RTHVTFRALHDLPPGTELAQSY........
Mr.Ashr2       FNHSCAPN.CTWSFVHESESKPG.....GGAVSVNVRAIKPVKSGDELTISY........
Mpu.Ashr2      FNHSCAPN.CTWHFAPPGSAD........SPVSVTVKAIRPIKKGDEATVCY........
Ol.Atxr4       LNHSCAPN.CHTHWENGDS.............SLTIRALREIAPGEELTITY........
At.Ashr1       INHSCSPNAVLVFE.....EQ...........MAVVRAMDNISKDSEITISY........
Mr.Ashr1b      FNHSCVPSAEFCNV.....GT...........SLTVRSLRRVQAGEEITVSY........
Os.Atxr1       INHSCHPNARRTHV.....GD...........HAIVHASRDIKAGEEITFAY........
Cr.Ashr1       ANHSCRPSTVQVFR.....GR...........TLQLRALRPLAPGQEVTLCY........
Cv.Ashr1       ANHDCRPNTLHAFR.....GG...........RMVFRAVRAIQPGEEVTTSY........
Cr.Ashr2       ANHSFRPNATFCMARD...NK...........RFELRLLGPLAPGEEAAISYGETKPNP.
Ot.Atxr4a      INHSCEPSCEVAFIH....DA...........RAHVIATRDITKGEEITISY........
Sm.Atxr4       FNHSCDANVNIYWRE....NA...........FAQLKALQPIEPGKELCITY........
At.Atxr4       YNHDCDPNAHIIWLH....NA...........DARLNTLRDVEEGEELRICYIDASMG..
Os.Atxr4       YNHDCDPNTHIVWLA....SA...........DARLKALRNIEEGEELRICYIDASMD..
Cv.Ashr2a      .NHAADPSCDFRLNAG...EG...........CVELVAVKDLQPGQEATISY........
Mpu.Ashr1      INHACAPNAAV.AVAG...ED...........VATAYALRAIEPGEEITVSY........
Ot.Atxr4b      CNHSCDPNAEVSAISD...QG...........EVTLYSLRPIAAGEEITICYG.......
Mr.Ashh1       DLLNHTSVSDEVNCEWVADDT...........YFVVSATRDVPKNGELLFKYG.......
Sm.Suvh2       FAIGPELVIDAEKYGNVARLR...........HITMFAAKDIAAGEELTFDYS.......
Cv.Atxr        TNHSCSPSMYSKILNIDGRQ............RLVFFARHDIEVGQELTYNY........
Pa.Atx1        INHSCMPNCYARIMSIDGEES...........RIVLIAKKDVAAGEELTYDYQFD.....
Pa.Atx4        INHSCAPNCYARIMVVDGVES...........RIALIAKKDVAAGEELTYDY........
Mp.Atxr2       .NHSCRPNAKAFKREED.RDG...........AAVLLAIRPIRKGEEITISY........
Nm.Atxr2       MNHSCAPNAKAFKRDED.KDG...........SAVLLTARAIKAGEEVRISY........
Os.Atxr2       MNHSCCPNAKAYKRDED.TDG...........NAVIIALEPIKKDDEITISYIDED....
Sm.atxr2       INHSCEPNAKAFKRDED.ING...........NAVIIATRKIMKGEQIFTSY........
Ol.Atxrb       FNHDCDPNCEPMKGEED.IDG...........ACVIIARRDIAAGEELTIS.........
At.Atxr2       ..........AFKREED.RDG...........QAVIIALRRISKNEEVTISY........
consensus>50   ge.......d.r................................................
```

Contd...

```
                          70        80
                           .         .
Pa.EZA1     ........FVLDAYRKGDKLKFANHSPN...........................PNCYA
Sm.CLF      ........YVLDACRKGDKLKFANHSPN...........................PNCYA
Pa.EZA2     ........YVLDAYRKGDKLKFANHSPT...........................PNCYA
Os.CLF      ........YVLDAYRMGDKLKFANHSPD...........................PNCYA
Nm.CLF      ........FVLDAYRKGDKLKFANHSPN...........................PNCYA
Mp.CLF      ........YVLDACRKGDKLKFANHSPN...........................PNCYA
Pp.CLF      .....ASLYVLDACRKGDKLKFANHSPT...........................PNCYA
Os.SWN      ........YVLDAYRKGDKLKFANHSSN...........................PNCYA
At.CLF      ........FVLDAYRKGDKLKFANHSPE...........................PNCYA
Mp.EZA      ........YVIDAYRMGSKFKFVNHSSK...........................PNCYP
At.SWN      ........YVLDAQRKGDKLKFANHSAK...........................PNCYA
Mpu.CLF     ........WCLDAHLKGNKLKFANHSAT...........................PNCYA
Mr.CLF      ........WVLDAAVRGNKLKFANHSAT...........................PNCYA
Kf.EZA      ........FILDACHKGDKLKFANHSPA...........................PNCRP
Ot.CLF      ........FCIDAQNRGNKLRFANHSVH...........................PNVRS
Ol.CLF      ........WCVDAQYRGNKLRFANHSKN...........................PNCVP
Cv.CLF      ........WVIDARQKGNTLRFANHSTT...........................ANCRA
At.MEA      ........LEIDARRKGNEFKFLNHSAR...........................PNCYA
Mp.MEA      ........FVIDAYRLGSKFKFANHSST...........................PNCYP
Pp.Atx6a    ...........DATRKGGIARFVNHSCS...........................PNCVA
Pp.Atx6b    ...........DATRNGGIARFVNHSCS...........................PNCVA
Pp.Atxd     ...........DATRKGGIARFVNHSCSLCMCGILEIANTSGLLGYGYLIAWNSWPNCVA
Mp.Atxra    ...........DATRKGGIARFVNHSCS...........................PNCVA
Os.Atx6     ...........DATRKGGIARFINHSCQ...........................PNCVA
Pp.Atxr7a   FIVLDHVPSQLDATRRGGLARFINHSCD...........................PNCYT
Pp.Atxr7b   ...........DATQKGGLARFINHSCN...........................PNCYT
Mp.Atxr7    ...........DATMRGGLARFINHSCD...........................PNCYT
Pa.Atxr7    ...........DATKRGGLARFINHSCE...........................PNCYT
Os.Atxr7    ...........DATKRGGLARFINHSCD...........................PNCYT
Nm.Atxr7    ...........DATRHGGLARFINHCCE...........................PNCVT
kf.Atxr7a   ...........DATKMGGAARFINHCCD...........................PNCYT
Sm.Atx5b    ...........DATKHGGLARFINHSCDARIL.......................PNCYT
Cp.Atxr     ...........DATKKGNTARFINHCCD...........................PNCVA
Sm.Atxr7    ...........DATQRGGLGRFINHSCE...........................PNCYP
At.Atxr7    ...........DATKRGGIARFINHSCE...........................PNCYT
Pp.Atx2a    ...........DATTTGSIAHLINHSCE...........................PNCYS
Pp.Atx2b    ...........DATRAGSIAHLINHSCE...........................PNCYS
Pp.Atx2c    ...........DATHAGTIAHLINHSCE...........................PNCYS
Mp.Atx2     ...........DATHAGSIAHLINHSCE...........................PNCYS
Nm.Atxa     ...........DATGAGTIAHLINHACE...........................PNCFS
Nm.Atxb     ...........DATGAGTIAHLINHACE...........................PNCFS
Sm.Atx2b    ...........DATRAGSIAHLINHSCE...........................PNCYS
Sm.Atx1     ...........DATHVGSMAHLINHSCE...........................PNCYS
Sm.Atx2c    ...........DATHVGSMAHLINHSCE...........................PNCYS
kf.Atxa     ...........DATKAGSLAHLINHACE...........................PNCFS
Cv.Atx      ...........DATRAGNLAHMLNHSCD...........................PNCFS
Cr.Atxr1    ...........DATKAGNMAHLLNHSCD...........................PNCYS
Vc.Atx5     ...........DATRAGNMAHLLNHSCD...........................PNCYS
At.Atx1     ...........DATRTGSIAHLINHSCV...........................PNCYS
At.Atx2     ...........DATRTGSIAHLINHSCE...........................PNCYS
Cr.Atxr     ...........DATLAGNAARFINHCCD...........................PNCVA
Vc.Atxr7    ...........DATLAGNAARFINHCCD...........................PNCVA
Cp.Atx2     ...........DATKKANKARFVNHSCD...........................PNCSM
Pp.Atx3     .........VIDATDKGNIARLINHSCD...........................PSCYA
Mp.Atx5     .........VIDATEKGNIGRLINHSCA...........................PNCFA
Sm.Atx2a    .........VIDATENGNIGRLINHSCE...........................PNCYA
Sm.Atx5a    .........VIDATEKGNIGRLINHSCS...........................PSCYA
Os.Atx5     .........VVDATDKGNVARLINHSCT...........................PNCYA
Os.Atx4     .........VVDATEKGNIARLINHSCM...........................PNCYA
At.Atx3     .........VIDATDSGNIARLINHSCM...........................PNCYA
At.Atxr3    .........VIDATDSGNIARLINHSCM...........................PNCYA
At.Atx4     .........VVDATDKGNIARLINHSCT...........................PNCYA
At.Atx5     .........VVDATEKGNIARLINHSCM...........................PNCYA
Nm.Atx5     .........VIDATEKGNRARLINHSCE...........................PNCHA
kf.Atxb     .........VVDATETGNLGRLINHSCG...........................PNCYS
Pa.Atx5     .........VIDATMKGNVARLINHSCA...........................ANCFS
Cr.Atxr7    .........VLDSTHLGHYGRFANHSCS...........................PSLYT
Vc.Atx2     .........VLDSTHLGHYGRFTNHSCS...........................PSLYT
Ol.Atxr3    .........VVDCTFQGNFARFTNHSCN...........................PNMYS
Ot.Atx      .........VVDCTFQGNLARFTNHSCN...........................PNMYS
Mpu.Atx5    .........VIDTTMRGNIARFTNHSCN...........................PNMYT
Mr.Atx5     .........VIDTTVKGSIARFTNHSCT...........................PNMYT
Pa.Ashh1    ...........DATRKGSIARFINHSCE...........................PNCET
Os.Ashh1    ...........DATKKGSLARFINHSCQ...........................PNCET
kf.Ashh4b   ...........DATKKGSYGRFINHSCN...........................PSCVT
At.Ashh1    ...........DATKKGSLARFINHSCR...........................PNCET
Mp.Ashh1    ...........DATHFGSLARFINHSCD...........................PNCET
Os.Ashh2    ...........DACTKGNLGRFINHSCS...........................PNCRT
Pp.Ashh2a   ...........DACSKGNLGRFINHSCE...........................PNCQT
Pp.Ashh2b   ...........DACNKGNLGRFINHSCE...........................PNCQT
Mp.Ashh3a   ...........DACSKGNFGRFINHSCE...........................PNCKT
Nm.Ashh2a   ...........DACRKGNLGRFINHSCE...........................PNCET
Sm.Ashh2c   ...........DACRKGNLGRFINHSCE...........................PNCQT
kf.Ashh1    ...........DACQKGNLGRFINHSCN...........................PNAEV
Cr.Ashh2    ...........DACRKGNISRFINHSCE...........................PNCET
Vc.Ashh1    ...........DACRKGNISRFINHSCE...........................PNCET
Cv.Ashh2    ...........DASRRGNLGRFINHSCE...........................PNCET
Ol.Ashh4    ...........DATIRGNEGRFLNHSCA...........................PNCET
Ot.Ashh2    ...........DATLRGNEGRFLNHSCA...........................PNCET
Mr.Ashh2b   ...........DAAERGNAGRFLNHSCD...........................PNCET
Mp.Ashh2a   ...........DASLKGNFGRFINHSCE...........................PNCKT
At.Ashh2    ...........DAGAKGNLGRFINHSCE...........................PNCRT
Os.Ashh3c   ...........DATNKGNMSRFINHSCE...........................PNTEM
Nm.Ashh2b   ...........DASYKGNASRFINHSCN...........................PNCEL
Sm.Ashr3    ...........DATFKGNMSRFINHSCN...........................PNAQL
Pp.Ashr3a   ...........DATFKGNLSRFINHSCQ...........................PNSEL
Mp.Ashh3b   ...........DATFKGNLSRFINHSCQ...........................PNCEL
Os.Ashh3b   ...........DATFKGNTSRFLNHSCD...........................PNCKL
Mp.Ashr3a   ...........DATFKGNSSRFLNHSCQ...........................PNCKL
Pp.Ashr3b   ...........DATRKGNASRYLNHSCQ...........................PNCRL
Nm.Ashr3    ...........DASFKGNLSRYINHSCE...........................PNCQL
Os.Ashh3a   ...........DATFKGNDCRFFNHSCE...........................PNCQL
At.Ashr3    ...........DATFKGNASRFLNHSCN...........................PNCVL
```

Contd...

```
At.Ashh4     ...........DATHKGNKSRYINHSCS...........................PNTEM
At.Ashh3     ...........DATHKGNKSRYINHSCN...........................PNTQM
kf.Ashh4a    ...........DAYYKGNLSRFVPHSAR...........................PNCQL
Ol.Ashh2     ...........DAKFKGSIARFINSSCH...........................PNCET
Ot.Ashh1     ...........DAKFKGSLARFINSGCH...........................PNCET
Mr.Ashh2a    ...........DARYKGNLSRFINSSCH...........................PNCET
Cv.Ashh4     ...........DARSKGNLARLLNSSCD...........................PNCET
Cv.Ashh1     ......IRLNIDATRRGNLARFFNHSCDG..........................GNLQ.
Pp.Suvr3a    ......VRINVDATKVGNVARFINHACDG..........................GNLL.
Sm.Suvr3     ......LRFNIDATNVGNIARFINHSCDG..........................GNLL.
Kf.Suvr3     ......LRLNIDATHFGNVARFVNHSCDG..........................GNAT.
Os.Suvr3     ......LRVNIDATKVGNVARFINHSCDG..........................GNLH.
At.Suvr3     ......LRINIDATRIGNVARFINHSCDG..........................GNLS.
Mpu.Suvr3    ......VKWAIDPIRRGNVGRFLNHACDG..........................GNLR.
Pp.Suvr3b    ......PDFTLDGFYASNVARFINHSC.D..........................PNLKI
Mp.Suvr5a    ......DAIVLDGINMSNIARFINHSC.E..........................GNLMI
Mp.Suvr5c    ......DAIVLDGINMSNIARFINHSC.E..........................GNLMI
Nm.Suvr5b    ......DMLVIDGFHMSNVARFINHSC.D..........................CNIAV
Pa.Suvr3     ......DHLVLDGFKMSNVGRFINHSC.D..........................GNLSI
Pp.Suvr5a    ......KPFVIDATKHGNVARFINHSC.A..........................PNLIN
Nm.Suvr5a    ......RPFVIDATKFGNVSRFINHSC.D..........................PNLVN
Mp.Suvr5b    ......KPFVIDATSQGNVARFINHSC.A..........................PNLVN
Sm.Suvr5     SRVPRIKPFVIDATKHGNVARFINHSC.S..........................PNLVN
Pa.Suvr5b    ......KPYVIDATKYGNVARFINHSC.S..........................PNLIN
Pa.Suvr5a    ......QPYVIDATKYGNVARFINHSC.L..........................PNLVN
Pp.Suvr5b    ......KPFVIDATKYGNVARFINHGC.E..........................PNLIN
Pp.Suvr5c    ......KPFVIDATKYGNVARFINHSC.E..........................PNLIN
Os.Suvr5     ......TAYVIDATRYGNVSRFINHSC.S..........................PNLST
At.Suvr5     ......LDYAIDATTHGNISRFINHSC.S..........................PNLVN
Pp.Suvr4b    RYLKDEEALCLDGTCYGNVARFINHGCFD..........................TNLLE
Pp.Suvr4a    ANLKDEEALCLDATYFGNVARFLNHRCLD..........................ANLME
Mp.Suvr4     KGLKDEEALCLDATNFGNVARFINHRCFD..........................ANLID
Os.Suvr4a    GVLKDEEALCLDATFYGNVARFINHRCFD..........................ANIIG
Os.Suvr4b    GVLKDEEALSLDSTFYGNVGRFINHRCYD..........................ANLVE
Sm.Suvr1     RILEDDHLLCLDCTHYGNVARFINHRCGD..........................SNLVD
At.Suvr4     KDLKDEEALCLDATICGNVARFINHRCED..........................ANMID
Kf.Suvr4     DCIDNKTGLCLDATKLGNVARFFNHRCED..........................ATLID
At.Suvr1     ERLEGDKALCLDGMFYGNISRFLNHRCLD..........................ANLIE
At.Suvr2     DISGDDKALSLEGTHYGNISRFINHRCLD..........................ANLIE
Nm.Suvr4     EGLGDDDLLNLDATVFGNVARFLNHRCED..........................ACLVM
Sm.Suvh5     ......NLVVVDAVAYGNVARFINHRCDG..........................GNIRV
Sm.Suvh10    ........LVIDAEKYGNVARFVNHSCDG..........................GNVHI
At.Suvh5     ......DPFTINAAQKGNIGRFINHSCSP...........................NLYA
Mpu.Atxr1    FTVDAAAGLIVDATRKGNVARFVNHC.CE..........................PNCVA
Os.Atx1      FRIDDER..VIDATRAGSIAHLINHS.CE..........................PNCYS
kf.Atxr3     SKQDHLEDLVVDATRRGNAARNINHS.CG..........................HNCVT
Pp.Suvh4a    LMMQDAPAYVLDAGKNGSVSRFLNHS.CE..........................PNVFI
Sm.Suvh4     AEHHGQAEFCLDGGECGAVARFINHS.CE..........................PNLFI
Os.Suvh4     DSDPPAPEYCIDAGSIGNFARFINHS.CE..........................PNLFV
At.Suvh4     SEDENAPEFCIDAGSTGNFARFINHS.CE..........................PNLFV
Pa.Suvh4     ...........DNGTAGGVARFINHS.CD..........................PNLFV
Kf.Suvh4b    KEHFEDVEWCLDAAKFGNITRFINHS.CD..........................PNLFV
Pp.Suvh4b    LVEELSAHYVIDCRLSGNVARFINHS.CN..........................PNLFV
kf.Suvh4a    ISKLPQVEYSIDALRAGNVARFVNHS.CT..........................PNAVV
Mp.Suvr3     KDYKTYDSWTIDARQYGNVARFINHS.CD..........................GNLFV
Sm.Suvh6     VVEDGHMS..IDAGSCGNVSRFINHS.CD..........................PNMFV
Nm.Suvh4     DLHDDNVDYCMDASKAGNVSRFANHS.CS..........................PNMFV
Pa.Suvh5b    AETVEDAGYTIDAAKYGGVGRFINHS.CS..........................PNLYA
Pa.Suvh5a    CDMVDDMGFTVDAANYGNVGRFVNHS.CS..........................PNLYA
Os.Suvh5c    SQTMEDVCFTIDASEYGNIGRFINHS.CS..........................PNLYA
Os.Suv5d     KD..EEASFAVDASKMGNFAKFINHS.CT..........................PNLYA
Os.Suvh5a    KD..EEAGFAVDASKMGNFAKFINHS.CT..........................PNLYA
Os.Suvh5b    KTTEDTEGSTIDASKCSNVGRFINHS.CS..........................PNLYA
At.Suvh6     AEGDESSGFTIDAASKGNVGRFINHS.CS..........................PNLYA
Pa.Suvh7b    QIQF...NIAIDASKMGNVSRFMNHS.CS..........................PNLFR
Pa.Suvh7c    RLEL...KFTINAREMGNVARFINHS.CS..........................PNVFL
Pa.Suvh7a    SLAFPALNFLIDANKFGNVSRFIKHS.SC..........................PNVFI
Os.Suvh7     FEPLP...IKISAMKMGNVARFMNHS.CN..........................PNTFW
Os.Suvh8     FEPLP...IKISAKKMGNVSRFMNHS.CS..........................PNVFW
Os.Suvh11    FKKLP...IIIRANNEGNVARFLNHS.CS..........................PNLLW
Os.Suvh1     PKQLP...IIISAKRTGNIARFMNHS.CS..........................PNVFW
Cv.Suvh4     QEDDDDEVLVIDAASTGNLARFINHS.CE..........................PNLVL
AtSuvh8      EDANLPTQVLISAKEKGNVGRFMNHN.CW..........................PNVFW
At.Suvh7     EFINLPTQVLISAKEKGNVGRFMNHS.CS..........................PNVFW
At.Suvh3     EEFNLPSPLLISAKKFGNVARFMNHS.CS..........................PNVFW
At.Suvh1     EESEIPLPLIISAKNVGNVARFMNHS.CS..........................PNVFW
Pa.Suvh2c    FQLVENKGFKVDASRSGNASRFMRRS.SR..........................PNIFV
Pa.Suvh2b    FQLVEDVGFKVDASSSGNASRFVRRS.SR..........................PNIFS
Pa.Suvh2a    FQLVEDKGYKVDASRSGNVCRFIRKSKSS..........................ANIFA
Os.Suvh2     NPAVPDLKFSIDVSRARNVACYFSHS.CS..........................PNVFV
Os.Suvh9     FPLFAGARYRLDVSQRRNVACYISHS.CS..........................PNVFL
AtSuvh2      YPSLPPLDFSMDVSRMRNVACYISHS.KE..........................PNVMV
At.Suvh9     YPDIPPVDFAMDVSKMRNVACYISHS.TD..........................PNVIV
Cv.Suvh2     VPDQAKAEFVACGLRKRNVGAFLNHS.CA..........................PNCFV
Mpu.Suvh4    VISMQYKEYTIDATMCGNVARMLNHN.CE..........................PNVTT
Mpu.Suvr5    KPKDEDLNLGVDARVTGGVARFINHH.SE..........................PNLII
Cr.CLF       .........VTPGMEITYDY.......................................
Pa.Suvh10a   .........ICEGEELTFCYGIVRKN.................................
Cp.Ashr2a    .........CNPNLEALKW........................................
Cp.Ashr2b    .........CDPNCVTRK.........................................
Mp.Ashh2b    .........CQPNCELQKWFI......................................
Pa.Suvr4     .........HYGNVARFINH.......................................
Cr.Ashh1     .........ILPGEELEYDYSF.....................................
Cr.Suva      ............................................................
Os.Suvh10    ............................................................
Pa.Suvh10b   ............................................................
Pa.Atx6      ............................................................
Cr.Suvb      ............................................................
Vr.Suv       ............................................................
At.Suvh10    ............................................................
Ot.Ashh4     PIKKG..EFL....IDFLGEIYPPWAWAEKQDA...................IRLVQKNR
Os.Atxr3     TGGFGVDDFV....IEFFGEVYPSWRWYEKQDG...................IKHIQNNS
Mpu.Atxr5    SSFLAPPDWSPYDRVRVVNALYPGWRWFEKEVA...................AQRVRRDV
```

Contd...

```
Pp.Atxr5        RQGFVVEADEDIKDMTFIAEYTGEVDYMCCRHY....................D.SGNSIM
Nm.Atxr5        KQGFVVEAADHIPDLTIIAEYTGDVDFMCNREN....................D.PGDSIM
Pa.Atxr6a       REGFTVEADNNIKDLTIITEYTGDVDYLRKREK....................D.DGDCMM
Pa.Atxr6b       REGFTVEADNNIKDFTIITEYTGDVDYLRKREN....................D.DGDCMM
At.Atxr6        ..GFTVEADRFIKDWTIITEYVGDVDYLSNRED....................DYDGDSMM
Os.Atxr6        ..GFTVEADRFIKDLTIITEYVGDVDYLTRREH....................D.DGDSMM
At.Atxr5        ..GYTVEADGPIKDLTFIAEYTGDVDYLKNREK....................D.DCDSIM
Os.Atxr5        ......QADADIKDMTFIAEYTGDVDFLENRAN....................D.DGDSIM
Cv.Ashr3b       SSCRLRFMNAYDGLRAEANVVCVEAEDVGERTP....................HAFLVTAA
Sm.Ashh1        LSRVAELLHYLD.GASRSSAPPPNRLLVPSGDM....................EDYMGDLS
At.Atxr1        CGRTRRVVSALSTGQGEDSLEIPEIALFRPDEA....................FETCGDWK
Cr.Suvc         VGRATDACVRGQGDRGGGAGRQRPRIGGDGGT.....................REG.GDSA
Mpu.Suvh10      SGRTTPTPTQVQKLLDLAGLSAADAETQFELDG....................KRA.GSFA
At.Ashr2        .............................................................
Sm.Ashr2        .............................................................
Nm.Ashr2        .............................................................
Cv.Ashr2b       .............................................................
Vc.Ashr2        .............................................................
Mr.Ashr2        .............................................................
Mpu.Ashr2       .............................................................
Ol.Atxr4        .............................................................
At.Ashr1        .............................................................
Mr.Ashr1b       .............................................................
Os.Atxr1        .............................................................
Cr.Ashr1        .............................................................
Cv.Ashr1        .............................................................
Cr.Ashr2        .............................................................
Ot.Atxr4a       .............................................................
Sm.Atxr4        .............................................................
At.Atxr4        .............................................................
Os.Atxr4        .............................................................
Cv.Ashr2a       .............................................................
Mpu.Ashr1       .............................................................
Ot.Atxr4b       .............................................................
Mr.Ashh1        .............................................................
Sm.Suvh2        .............................................................
Cv.Atxr         .............................................................
Pa.Atx1         .............................................................
Pa.Atx4         .............................................................
Mp.Atxr2        .............................................................
Nm.Atxr2        .............................................................
Os.Atxr2        .............................................................
Sm.atxr2        .............................................................
Ol.Atxrb        .............................................................
At.Atxr2        .............................................................
consensus>50    ...........da...gn..rfinhsc.............................pn...
```

Contd...

```
                  90                 100                 110
                  .                   .                   .
Pa.EZA1      KVIM.....VAG.......DHRVGIFAK.....ERIASG..........EELFYDYRYE
Sm.CLF       KVIM.....VAG.......DHRVGIFAK.....ERISAG..........EELFYDYRYE
Pa.EZA2      KVIM.....VAG.......DHRVGIFAK.....ERIAAG..........EELFYDYRYE
Os.CLF       KVIM.....VAG.......DHRVGIFAK.....ERISAG..........EELFYDYRYE
Nm.CLF       KVMM.....VAG.......DHRVGIFAK.....ERIAAG..........EELFYDYRYE
Mp.CLF       KVIM.....VAG.......DHRVGIFAK.....ERINAG..........EELFYDYR..
Pp.CLF       KVIM.....VSG.......DHRVGIFAK.....ERIGAG..........EELFYDYQYE
Os.SWN       KVML.....VAG.......DHRVGIYAK.....DRIEAS..........EELFYDYRY.
At.CLF       KVIM.....VAG.......DHRVGIFAK.....ERILAG..........EELFYDY...
Mp.EZA       KIVK.....VAG.......EHRVGIFAK.....RKIEAG..........EELFFDYGYE
At.SWN       KVMF.....VAG.......DHRVGIFAN.....ERIEAS..........EELFYDY...
Mpu.CLF      KVLM.....VRG.......DHRVGIFAK.....DNIAPG..........EELTYDYRYE
Mr.CLF       KVLM.....VRG.......DHRVGIFAK.....EHIAPG..........EELTYDYRYE
Kf.EZA       KVVS.....VLG.......DHRVAIHVE.....RNMESG..........EEIFYDYN..
Ot.CLF       AVMA.....VNG.......DNRLAMFAL.....RDIAPG..........EELFFDYRY.
Ol.CLF       RVLA.....VNG.......DHRLALISD.....KDIKPG..........DELLFDYNY.
Cv.CLF       EILM.....VDG.......DHRVAIVAN.....RDVAAG..........DELFYNYNYD
At.MEA       KLMI.....VRG.......DQRIGLFAE.....RAIEEG..........EELFFDY...
Mp.MEA       KVVL.....VAG.......EH.........................................
Pp.Atx6a     KVIC.....VEN.......LKKVVFFAK.....RDIYAG..........EEVTYDYKFN
Pp.Atx6b     KVIC.....VEN.......LKKVIFFAK.....RNIDAG..........EEVTYDYKFN
Pp.Atxd      KVIC.....VEN.......LKKVVFFAK.....RNIYAA..........EEVTYDYKFN
Mp.Atxra     KVIC.....VKS.......QNKVVFFAQ.....RDIYAG..........EEITYDYQFN
Os.Atx6      KIIS.....VRN.......EKKVVFFAE.....RHINPG..........EEITYDYHFN
Pp.Atxr7a    KIIT.....VEG.......QKKVVIYSK.....QRIVPG..........EELTYDYKFS
Pp.Atxr7b    KIIT.....VEG.......RKKVVIYSK.....RAIGAG..........EELTYDYKFS
Mp.Atxr7     KIIT.....VDG.......QKKVVIYSK.....RHIRAG..........EELTYDYKFP
Pa.Atxr7     KIIN.....VEG.......QKKIFIYAK.....KPILAG..........EELTYNYKFP
Os.Atxr7     KVIT.....VEG.......QKKIVIYAK.....RRIYAG..........EELTYNYKFP
Nm.Atxr7     KIIN.....VEG.......QKKIVIYSK.....RLIAPG..........EELTYDYKFP
kf.Atxr7a    KVIW.....VEG.......QKKIVIYSK.....RRIEPG..........EELTYDYKFP
Sm.Atx5b     KILT.....VEG.......QKRIFIYSK.....KHIKVG..........EELTYDYKFP
Cp.Atxr      KIIT.....VDG.......AKKIVIYAK.....RDIAVG..........EEITYDYKFP
Sm.Atxr7     KIIT.....VEG.......QKRVFIYAR.....THIAPG..........TELTYDYKFP
At.Atxr7     KIIS.....VEG.......KKKIFIYAK.....RHIDAG..........EEISYNY...
Pp.Atx2a     RTVT.....ASG.......EDRIIIFAK.....RDLEIG..........EELTYDYRF.
Pp.Atx2b     RTVT.....ASG.......EDRIIIFAK.....RNIEIG..........EELTYDYRF.
Pp.Atx2c     RTVT.....ASG.......EDRIIIFAK.....RNIEVG..........EELTYDYRF.
Mp.Atx2      RVVA.....ASG.......EDHIIIFAK.....RDIPEG..........VELTYDYRFA
Nm.Atxa      KMVQ.....VNG.......EERIIIFAK.....RNLAAG..........EELTYDYRFN
Nm.Atxb      KMVQ.....MKG.......EQRIIIFAK.....RDLAAG..........EELTYDYRFN
Sm.Atx2b     RVVT.....TNG.......KERIVIFAK.....QDIAGG..........DEVTYDYRF.
Sm.Atx1      RIIT.....VDA.......KDSIIIFAK.....RDIHPW..........EELTYDYRFA
Sm.Atx2c     RIIT.....VDA.......KDSIIIFAK.....RDIHPW..........EELTYDYRFA
kf.Atxa      RVVR.....ADG.......DEHIVITAK.....RDILPG..........EEFTYDYRFN
Cv.Atx       RTIR.....V.........VDHVIIFAK.....KDIEVA..........EELTYDYRF.
Cr.Atxr1     RAIT.....LTD.PLTGATTDHVIITAK.....RDLQPW..........EELTYDYRFN
Vc.Atx5      RTIT.....LTD.PVTRATSDHVIITAKVCVLWRDIEAW..........EELTYDYRFN
At.Atx1      RVIT.....VNG.......DEHIIIFAK.....RHIPKW..........EELTYDY...
At.Atx2      RVIS.....VNG.......DEHIIIFAK.....RDVAKW..........EELTYDY...
Cr.Atxr      RIVE.....VGG.......EKKIAIYTL.....RRVEAG..........EELAYDYKLS
Vc.Atxr7     RIVE.....VGG.......EKRIAIYTL.....RRVEAG..........EELHYDYK..
Cp.Atx2      TIIT.....IRN.......KSKIVCLAA.....RDIEES..........QEITYDYKFE
Pp.Atx3      KILD.....FQRDDGEGDS..RIVLIAR.....KYIAAG..........EELTYNYRFD
Mp.Atx5      KIWE.....ASRDGEKGSETHHIMLIAR.....KDVAAG..........EELTYNYRFA
Sm.Atx2a     RIVS.....VEGEGES.....HIVLIAR.....KDVSVG..........EELTYDYQFD
Sm.Atx5a     RILC.....VDGE.ES.....RIVLIAK.....RNVAAG..........EELTYDYQFE
Os.Atx5      RIMS.....VGHD.ES.....RIVLIAK.....KNVSAG..........EELTYDYLFD
Os.Atx4      RIMS.....VGDE.KS.....QIILIAK.....RDVSAG..........EELTYDYLFD
At.Atx3      RIVS.....MGDGEDN.....RIVLIAK.....TNVAAG..........EELTYDY...
At.Atxr3     RIVS.....MGDGEDN.....RIVLIAK.....TNVAAG..........EELTYDY...
At.Atx4      RIMS.....VGDEES......RIVLIAK.....ANVAVG..........EELTYDY...
At.Atx5      RIMS.....VGDDES......RIVLIAK.....TTVASC..........EELTYDY...
Nm.Atx5      KILE.....VEGQN.......RIILVAR.....KDISAG..........EELTYDYRFD
kf.Atxb      QITR.....VDGIT.......RIILRAK.....RDVQAG..........EELTYDYRFA
Pa.Atx5      RILA.....FNGVDS......RIAIFAK.....KDIEAG..........EEITYDYRLS
Cr.Atxr7     KVLE.....FEGGR.....RVRLAFCAR.....VDIRAG..........QELTFDYRF.
Vc.Atx2      KVLE.....FEGGR......VRLVFCAR.....TDIRAG..........QELTFDYRF.
Ol.Atxr3     KIVK.....VDDAN.......HIIFFAR.....TDVRPG..........EELTYNYRFE
Ot.Atx       KIVK.....VDGEN.......HIIFFAR.....NDIKAG..........EELTYNYRFE
Mpu.Atx5     KIVS.....VDGSN.......HIIFFAR.....VDVQPG..........EEMTYDYRFD
Mr.Atx5      KLVA.....LDGDS.......RIFFFTR.....IDVHPG..........QELTYNYRFD
Pa.Ashh1     RKWT.....VLG.......ESRVGIFAK.....RDIPCG..........TELAYDYKFE
Os.Ashh1     RKWN.....VLG.......EVRVGIFAK.....QDIPIG..........TELSYDYNFE
kf.Ashh4b    KKWN.....VLG.......EVRVGVFAH.....RDIPRG..........EELTYDYDFE
At.Ashh1     RKWN.....VLG.......EVRVGIFAK.....ESISPR..........TELAYDYN..
Mp.Ashh1     AKWT.....VLG.......EERIGIFAI.....QDISAG..........TELTFDYKYT
Os.Ashh2     EKWM.....VNG.......EVCIGIFAM.....RNIKKG..........EELTFDYNYV
Pp.Ashh2a    EKWM.....VDG.......EVCIGLFAI.....RDVKKG..........EEVTFDYNFV
Pp.Ashh2b    EKWM.....VDG.......EVCIGLFAI.....RDIKER..........EEVTFDYNFV
Mp.Ashh3a    EKWM.....VNG.......EVCIGLFAI.....RDIAEG..........EEVTFDYNYV
Nm.Ashh2a    QKWI.....VKG.......EVCIGLFSK.....RDISAG..........EEITFNYNYE
Sm.Ashh2c    EKWC.....VNG.......EICIGLFAI.....RDVAKN..........EEITFNYNFE
kf.Ashh1     QKWQ.....VRG.......ELCVGLFTL.....RDVVAG..........EELTFDYNYD
Cr.Ashh2     QKWL.....VHG.......ELAIGLFAV.....RDIPKD..........TELTFDYNFE
Vc.Ashh1     QKWL.....VRG.......ELAIGLFAV.....RDIPKD..........TELTFDYNFE
Cv.Ashh2     QKWV.....VHG.......ELAIGLFTL.....EDISAG..........TELTFDYNFE
Ol.Ashh4     QKWM.....VRG.......ELCIGIFAT.....RDIEEG..........EELTIDYKFE
Ot.Ashh2     QKWM.....VRG.......ELRIGIFAT.....RDVQAG..........EELTIDYKFE
Mr.Ashh2b    QKWM.....VNG.......ELCIGIYAL.....TDIDAG..........DELTFDYNFE
Mp.Ashh2a    EKWM.....VDG.......EICIGVFAI.....TDLEKG..........EEITIDYNYV
At.Ashh2     EKWM.....VNG.......EICVGIFSM.....QDLK.....................
Os.Ashh3c    QKWT.....VEG.......ETRVGIFAL.....RDIKTG..........EELTYDYKFV
Nm.Ashh2b    QKWE.....MEG.......SLRVGVFAV.....KNIAKG..........VHLTYDYQFI
Sm.Ashr3     RKWQ.....CDG.......ELRIGVFAV.....SRILKG..........QEITYDYKYI
Pp.Ashr3a    QKWD.....IDG.......ETRIGVFAI.....TDIKRG..........DFVTYDYQFI
Mp.Ashh3b    QKWQ.....IDG.......EIRIGVFST.....VDIKKG..........DYVNYDYQFI
Os.Ashh3b    EKWQ.....VDG.......ETRVGVFAS.....RSIQVG..........EHLTYDYRYS
Mp.Ashr3a    EKWR.....VDG.......ETRAGVFAL.....RDIKLG..........EPLTYDYKYV
Pp.Ashr3b    EKWR.....VDG.......ETRVGVFAG.....RNIIAG..........EELTYDYKYV
Nm.Ashr3     QKWQ.....VDG.......ETRIGVFAS.....VDIPFG..........ESLTYDYKFV
Os.Ashh3a    QKWQ.....VNG.......KTRLGVFAS.....KAIEVG..........EPLTYDYRFE
At.Ashr3     EKWQ.....VEG.......ETRVGVFAA.....RQIEAG..........EPLTYDY...
```

Contd...

```
At.Ashh4     QKWI......IDG.......ETRIGIFAT.....RFINKG..........EQLTYDY...
At.Ashh3     QKWI......IDG.......ETRIGIFAT.....RGIKKG..........EHLTYDY...
kf.Ashh4a    QRWR......VDG.......ELRVGLFAT.....EPIRKG..........DAIAYDYK..
Ol.Ashh2     QRWV....DASTN.......ETRVGIFAT.....EDIASG..........TELTYDYNFA
Ot.Ashh1     QRWV....DASTN.......ETRVGIFAI.....EDIPAG..........TELTYDY...
Mr.Ashh2a    QKWQ....DSATG.......ETRVGIFAI.....QDIPEG..........TELTYDYN..
Cv.Ashh4     QKWH....DAGNS.......EVRVGIFSL.....RDVLPG..........EELTYDYQF.
Cv.Ashh1     .LLLA......RHTGCLLP...RVVFVTS.....RAVQQG..........EELTC.....
Pp.Suvr3a    .PCLV......RASGSVIP...RLALFAR.....QDIHDG..........EELRYSYGS.
Sm.Suvr3     .SCLV......RSAGCCVP...RLAFFTR.....KEIQSG..........QELTFSYG..
Kf.Suvr3     .RVLV......RQTGWPLP...HVALFAN.....REILAG..........EEITMSYGEV
Os.Suvr3     .PVLV......RSSGSLLP...RLCFFAA.....RDIIEG..........EELTFSYGD.
At.Suvr3     .TVLL......RSSGALLP...RLCFFAA.....KDIIAE..........EELSFSYG..
Mpu.Suvr3    .PMTL.........GPAPA...RIAFFAS.....EDIERG..........EELRWKYGEP
Pp.Suvr3b    YRVYV......ETTYKWLS...HIGMYAM.....RDIEPG..........EELSYDY...
Mp.Suvr5a    FRVFS......ETTDLRYF...RIGMYAI.....QDIEVG..........DELSYDYNYA
Mp.Suvr5c    FRVFS......ETTDLRYF...RIGMYAI.....QDIEVG..........DELSYDYNYA
Nm.Suvr5b    YRVYV......DMIDRRLA...HLGMYAV.....KDIEVG..........DELAYDYNY.
Pa.Suvr3     YRVYT......ETLDKRIF...RIGLYAG.....RDIEIG..........EELTYDYKY.
Pp.Suvr5a    YEVLV......ESMDCQLA...HIGFFAN.....RDISAG..........EELAYDYRY.
Nm.Suvr5a    YQVLV......ESMDVRLA...HVGLYAN.....SDIKAG..........DELAYDYRY.
Mp.Suvr5b    YQVLV......ESMDAQLA...HIGLFAS.....RDIEIG..........EELAYDYRYR
Sm.Suvr5     YQVLV......ESMDYQLA...HIGLFAS.....RDILCG..........EELSYDYRY.
Pa.Suvr5b    YQVLI......ESMDCQLA...HIGLYAS.....RDITAG..........EELAYDY...
Pa.Suvr5a    YQVLV......ESMDCQLA...HIGLYAS.....RDIAAG..........EELAYDYRY.
Pp.Suvr5b    YEVLV......ESLDCQLA...HIGFFAK.....RDIAPG..........EELAYDFRY.
Pp.Suvr5c    YEVLV......ESMDCQLA...HIGFFAN.....RDIAIG..........EELAYDYRY.
Os.Suvr5     RLVSV......ESKDCQLA...HIGLFAN.....QDILMG..........EELAYDYG..
At.Suvr5     HQVIV......ESMESPLA...HIGLYAS.....MDIAAG..........EEITRDYG..
Pp.Suvr4b    VPVEI......ESPDHHYY...HLAFFTS.....KDVAAN..........EELIWDYGLD
Pp.Suvr4a    MPVEI......ESPDRHYY...HVAFFTN.....RHVKAK..........EELTWDYGID
Mp.Suvr4     MPVTI......ESPDRHYY...HVAFFTS.....RAVRAN..........EELTWDYKID
Os.Suvr4a    IPVEI......ETPDHHYY...HLAFFTT.....RIIEPF..........EELTWDYGID
Os.Suvr4b    IPVEV......ETPDHHYY...HLAFFTT.....KKVEAF..........EELTWDYGID
Sm.Suvr1     IPVEI......ECPDRHFY...HASFSIS.......FRAM..........EELTWDYQLD
At.Suvr4     IPIEI......ETPDRHYY...HIAFFTL.....RDVKAM..........DELTWDY...
Kf.Suvr4     VPVEI......EGPKHNYY...HLAFFTN.....CFVEAG..........TELTWDYGID
At.Suvr1     IPVQV......ETPDQHYY...HLAFFTT.....RDIEAM..........EELAWDYG..
At.Suvr2     IPVHA......ETTDSHYY...HLAFFTT.....REIDAM..........EELTWDYG..
Nm.Suvr4     RPVSI......ETRDTHYY...HVAFFTG.....RDVEPM..........EELTWDYGIQ
Sm.Suvh5     EHVPY......EGLDDDERPMFHIMMFAS.....RDIKVG..........EELCFDY...
Sm.Suvh10    ECVSY......GHHDGRLR...HITMFAA.....KDIAAS..........EELTFDY...
At.Suvh5     QDVLY......DHEEIRIP...HIMFFAL.....DNIPPL..........QELSYDYN..
Mpu.Atxr1    KSVHV.............EGARRVVLFTT.....RDVAAG..........EELFYDYQ..
Os.Atx1      RVISV.............LGDEHIIIFAK.....RDINPW..........EELTYDYRFV
kf.Atxr3     KAVVI.............NGVRHLIVFAI.....RTIMPG..........EEITINYCFR
Pp.Suvh4a    QCVLS........H.HNDVTMPRIVMFAA.....DNIHPLELIDCVPWNPQELCYDYGY.
Sm.Suvh4     QCVLS........T.HHDMRIPRIVLFAA.....DNIAPL..........QELSYDYGYA
Os.Suvh4     QCVLS........S.HNDVKLAKVTLFAA.....DTILPL..........QELSYDYGYV
At.Suvh4     QCVLS........S.HQDIRLARVVLFAA.....DNISPM..........QELTYDYG..
Pa.Suvh4     QCVLS........S.HHDPKLARVMLCAA.....DNIPPL..........QELTYDYGYR
Kf.Suvh4b    QSVFV........E.HHDVREPRLFLVAM.....QNIKPL..........EELSYDYGY.
Pp.Suvh4b    QGVLH........D.HGDLNRGHIMLFAG.....EDIAAG..........TELAYDYGYE
kf.Suvh4a    QSVLF........D.HHNPSLSHIALFAY.....DNIPPG..........REITYDYN..
Mp.Suvr3     QCVFY........E.HHDMRFPHIMLFAM.....EKISPF..........KELTYDYGEE
Sm.Suvh6     QCVFN........D.HNDMAYPHVMMFAM.....KNIRPF..........EELSYDYGYE
Nm.Suvh4     QCVLH........D.HHDLELPHICLFAG.....EHIPAL..........QELTYDYAYE
Pa.Suvh5b    QNVLY........D.HDDKTVPHIMLFAA.....ENIPPL..........QELSYHYNY.
Pa.Suvh5a    QNVLY........D.HDDRKLPHIMLFAA.....ENIPPM..........RELFYHYNY.
Os.Suvh5c    QNVLW........D.HDDQRVPHIMFFAA.....ENIPPL..........QELTYDYNYK
Os.Suv5d     QNVLY........D.HDDKSVPHIMFFAC.....EDIPPR..........QELSYHYNY.
Os.Suvh5a    QNVLY........D.HDDKSVPHIMFFAC.....EDIPPR..........QELSYHYNY.
Os.Suvh5b    QNVLW........D.HDDMKKPHIMFFAT.....ENIPPL..........QELTYDYNY.
At.Suvh6     QNVLY........D.HEDSRIPHVMFFAQ.....DNIPPL..........QELCYDYN..
Pa.Suvh7b    QSILY........D.HHDTQFPHIMFFAL.....ENIPPL..........TELTYDYC..
Pa.Suvh7c    QSVVY........D.HQDTRFPHIMLFSI.....EHIPPL..........TELTLDYG..
Pa.Suvh7a    QFVLH........D.HLDVRFPHVMLFAR.....ENIPPL..........TELTFDYGSV
Os.Suvh7     QPVQF........D.HGEDGYPHIMFFAL.....KHIPPM..........TELTYDYGD.
Os.Suvh8     QPVQH........D.HGDDSHPHIMFFAL.....KHIPPM..........TELTFDYG..
Os.Suvh11    QAVQY........D.HGDDSYPHIMFFAM.....EHIPPM..........TELTYDYG..
Os.Suvh1     QPVLY........D.HGDEGYPHIAFFAI.....KHIPPM..........TELTYDYG..
Cv.Suvh4     NPVLR........P.GDSGMRYCVAIFAG.....RDIPRG..........TELCYDYGY.
AtSuvh8      QPIEY........DDNNGHIYVRIGLFAM.....KHIPPM..........TELTYDYG..
At.Suvh7     QPIEY........E.NRGDVYLLIGLFAM.....KHIPPM..........TELTYDYG..
At.Suvh3     QPVIR........E.GNGESVIHIAFFAM.....RHIPPM..........AELTYDYG..
At.Suvh1     QPVSY........E.NNSQLFVHVAFFAI.....SHIPPM..........TELTYDYG..
Pa.Suvh2c    QYVLH........D.HHEKDIPHVMLFAL.....ENIAPQ..........LELSIS....
Pa.Suvh2b    QYVLH........D.HHDKRIPHVMLFAL.....ENISPR..........LELSIN....
Pa.Suvh2a    QYVLH........D.HRNINIPHVMLFAL.....QNIPPE..........LELFIS....
Os.Suvh2     QFVLF........D.HYNAAYPHLMIFAM.....ENIPPL..........RELSIDYG..
Os.Suvh9     QYVIR........G.NEDESYPHMMVFAM.....ETIPPM..........RDLSIDYGLD
AtSuvh2      QFVLH........D.HNHLMFPRVMLFAL.....ENISPL..........AELSLDYG..
At.Suvh9     QFVLH........D.HNSLMFPRVMLFAA.....ENIPPM..........TELSLDYG..
Cv.Suvh2     QPVL.........DTHHDRRCPKICIFAS.....ENIAPM..........TELTLDYGEA
Mpu.Suvh4    LEVAVVRDPALSGDAARVPKLPRVGFFAT.....RDIEAN..........EELCIDYSP.
Mpu.Suvr5    QSVFT...PGAEGCRANNQRLYRICLFAG.....RDIGAM..........EELTYDYG..
Cr.CLF       ............................................................
Pa.Suvh10a   ............................................................
Cp.Ashr2a    ............................................................
Cp.Ashr2b    ............................................................
Mp.Ashh2b    ............................................................
Pa.Suvr4     ............................................................
Cr.Ashh1     ............................................................
Cr.Suva      ............................................................
Os.Suvh10    ............................................................
Pa.Suvh10b   ............................................................
Pa.Atx6      ............................................................
Cr.Suvb      ............................................................
Vr.Suv       ............................................................
At.Suvh10    ............................................................
Ot.Ashh4     GLRDKGPPEFYNMQIERPGGDEEGYSVLFCDAMHENNYAGRLSHTCDPNVEVNLKAINGK
Os.Atxr3     ....DDQAPEFYNIMLERPKGDRDGYDLVFVDAMHKANYASRICHSCNPNCEAKVTAVDGH
Mpu.Atxr5    R.DDEDVPVFYNAAVERDVADPKGYDMLFVDGMVKGSLLTRASHSCEPNAEMRVRVREGS
```

Contd...

```
Pp.Atxr5        GLLFSDDPIKELVICP....DKRSNIARFLSGINNHTEEGRK....KQNVRCVRYSINGE
Nm.Atxr5        GLLFTGDPNTELVICP....DKRGNVARFLSGINNHNKDGRK....KINVRCVRFDLDGE
Pa.Atxr6a       TLLFTGDSSKDLVICP....DKRGNIARFINGINNHTAKGKK....KQNLKCVRYNVDGE
Pa.Atxr6b       TLLSTGISSKDLVICP....DKCGNIARFINGINNHTAKGKK....KENLKCVRYNVDGE
At.Atxr6        TLLHASDPSQCLVICP....DRRSNIARFISGINNHSPEGRK....KQNLKCVRFNINGE
Os.Atxr6        TLLSAATPSRSLVICP....DKRSNIARFINGINNHTPDGRK....KQNLKCVRFDVGGE
At.Atxr5        TLLLSEDPSKTLVICP....DKFGNISRFINGINNHNPVAKK....KQNCKCVRYSINGE
Os.Atxr5        TLLLTEDPSKRLVICP....DKRGNISRFINGINNHTLDGKK....KKNIKCVRYDIDGE
Cv.Ashr3b       EVSAGEELLLDYG...............................................
Sm.Ashh1        TAMDPELESRVEAVVRAYTERGHPSLSPRNV......LWPLLTLINHCCVPTITLRVVGF
At.Atxr1        QSLDTEKLLSILDVNSLVEDAVSGKVMGKNKEYYGVGLWTLASFINHSCIPNARRLHVGD
Cr.Suvc         DGAGAGEGHALLVVREVLPSGLALRLNIDATRLGNVARFFNHSCDGGCLLPVVVRRRGSL
Mpu.Suvh10      RFINSSDQPNLFAQAVVTGHLDPRQCRICLFACFDIPAMTELSYDYGSEY..........
At.Ashr2        ............................................................
Sm.Ashr2        ............................................................
Nm.Ashr2        ............................................................
Cv.Ashr2b       ............................................................
Vc.Ashr2        ............................................................
Mr.Ashr2        ............................................................
Mpu.Ashr2       ............................................................
Ol.Atxr4        ............................................................
At.Ashr1        ............................................................
Mr.Ashr1b       ............................................................
Os.Atxr1        ............................................................
Cr.Ashr1        ............................................................
Cv.Ashr1        ............................................................
Cr.Ashr2        ............................................................
Ot.Atxr4a       ............................................................
Sm.Atxr4        ............................................................
At.Atxr4        ............................................................
Os.Atxr4        ............................................................
Cv.Ashr2a       ............................................................
Mpu.Ashr1       ............................................................
Ot.Atxr4b       ............................................................
Mr.Ashh1        ............................................................
Sm.Suvh2        ............................................................
Cv.Atxr         ............................................................
Pa.Atx1         ............................................................
Pa.Atx4         ............................................................
Mp.Atxr2        ............................................................
Nm.Atxr2        ............................................................
Os.Atxr2        ............................................................
Sm.atxr2        ............................................................
Ol.Atxrb        ............................................................
At.Atxr2        ............................................................
consensus>50    ..v....................i..fa.......di..g..........eel.ydy...
```

Contd...

```
Pa.EZA1      .........................
Sm.CLF       .........................
Pa.EZA2      P........................
Os.CLF       .........................
Nm.CLF       .........................
Mp.CLF       .........................
Pp.CLF       .........................
Os.SWN       .........................
At.CLF       .........................
Mp.EZA       K........................
At.SWN       .........................
Mpu.CLF      RDKA.....................
Mr.CLF       .........................
Kf.EZA       .........................
Ot.CLF       .........................
Ol.CLF       .........................
Cv.CLF       K........................
At.MEA       .........................
Mp.MEA       .........................
Pp.Atx6a     CDE......................
Pp.Atx6b     YDE......................
Pp.Atxd      CDEH.....................
Mp.Atxra     FEQEGDK..................
Os.Atx6      REDE.....................
Pp.Atxr7a    LEEVKIPCFCGAAK...........
Pp.Atxr7b    LEDK.....................
Mp.Atxr7     LEEK.....................
Pa.Atxr7     LEEK.....................
Os.Atxr7     LEEK.....................
Nm.Atxr7     IEEK.....................
kf.Atxr7a    FEN......................
Sm.Atx5b     FEE......................
Cp.Atxr      IE.......................
Sm.Atxr7     HED......................
At.Atxr7     .........................
Pp.Atx2a     .........................
Pp.Atx2b     .........................
Pp.Atx2c     .........................
Mp.Atx2      SKDE.....................
Nm.Atxa      SKDE.....................
Nm.Atxb      AKDE.....................
Sm.Atx2b     .........................
Sm.Atx1      SKGA.....................
Sm.Atx2c     SKGA.....................
kf.Atxa      SKDE.....................
Cv.Atx       .........................
Cr.Atxr1     SA.......................
Vc.Atx5      SSEE.....................
At.Atx1      .........................
At.Atx2      .........................
Cr.Atxr      C........................
Vc.Atxr7     .........................
Cp.Atx2      REE......................
Pp.Atx3      .........................
Mp.Atx5      PEDQKLLCLCGAPTCSQYINV....
Sm.Atx2a     KEDK.....................
Sm.Atx5a     .........................
Os.Atx5      .........................
Os.Atx4      .........................
At.Atx3      .........................
At.Atxr3     .........................
At.Atx4      .........................
At.Atx5      .........................
Nm.Atx5      .........................
kf.Atxb      QEED.....................
Pa.Atx5      KGD......................
Cr.Atxr7     .........................
Vc.Atx2      .........................
Ol.Atxr3     SED......................
Ot.Atx       SE.......................
Mpu.Atx5     .........................
Mr.Atx5      .........................
Pa.Ashh1     WYG......................
Os.Ashh1     WFG......................
kf.Ashh4b    WYG......................
At.Ashh1     .........................
Mp.Ashh1     WFG......................
Os.Ashh2     R........................
Pp.Ashh2a    R........................
Pp.Ashh2b    R........................
Mp.Ashh3a    R........................
Nm.Ashh2a    RHG......................
Sm.Ashh2c    R........................
kf.Ashh1     KSRK.....................
Cr.Ashh2     RYGD.....................
Vc.Ashh1     RYGD.....................
Cv.Ashh2     RYGD.....................
Ol.Ashh4     RFGE.....................
Ot.Ashh2     RFG......................
Mr.Ashh2b    RYGD.....................
Mp.Ashh2a    FA.......................
At.Ashh2     .........................
Os.Ashh3c    QFGA.....................
Nm.Ashh2b    QFG......................
Sm.Ashr3     QFGT.....................
Pp.Ashr3a    QFG......................
Mp.Ashh3b    QFGT.....................
Os.Ashh3b    S........................
Mp.Ashr3a    .........................
Pp.Ashr3b    .........................
Nm.Ashr3     GYE......................
Os.Ashh3a    QH.......................
At.Ashr3     .........................
```

Contd...

```
At.Ashh4      .........................
At.Ashh3      .........................
kf.Ashh4a     .........................
Ol.Ashh2      HFGD.....................
Ot.Ashh1      .........................
Mr.Ashh2a     .........................
Cv.Ashh4      .........................
Cv.Ashh1      .........................
Pp.Suvr3a     .........................
Sm.Suvr3      .........................
Kf.Suvr3      KADE.....................
Os.Suvr3      .........................
At.Suvr3      .........................
Mpu.Suvr3     KKFA.....................
Pp.Suvr3b     .........................
Mp.Suvr5a     SSKD.....................
Mp.Suvr5c     SSKD.....................
Nm.Suvr5b     .........................
Pa.Suvr3      .........................
Pp.Suvr5a     .........................
Nm.Suvr5a     .........................
Mp.Suvr5b     L........................
Sm.Suvr5      .........................
Pa.Suvr5b     .........................
Pa.Suvr5a     .........................
Pp.Suvr5b     .........................
Pp.Suvr5c     .........................
Os.Suvr5      .........................
At.Suvr5      .........................
Pp.Suvr4b     FNDK.....................
Pp.Suvr4a     FGDE.....................
Mp.Suvr4      FDDN.....................
Os.Suvr4a     FDD......................
Os.Suvr4b     FGD......................
Sm.Suvr1      FADE.....................
At.Suvr4      .........................
Kf.Suvr4      FEDE.....................
At.Suvr1      .........................
At.Suvr2      .........................
Nm.Suvr4      FDGE.....................
Sm.Suvh5      .........................
Sm.Suvh10     .........................
At.Suvh5      .........................
Mpu.Atxr1     .........................
Os.Atx1       SSDQRLPCYCGFPKCRGVVNDVEA.
kf.Atxr3      .........................
Pp.Suvh4a     .........................
Sm.Suvh4      LNS......................
Os.Suvh4      LDS......................
At.Suvh4      .........................
Pa.Suvh4      LDS......................
Kf.Suvh4b     .........................
Pp.Suvh4b     LNS......................
kf.Suvh4a     .........................
Mp.Suvr3      .........................
Sm.Suvh6      IDS......................
Nm.Suvh4      .........................
Pa.Suvh5b     .........................
Pa.Suvh5a     .........................
Os.Suvh5c     IGEVR..DLNGRVKVKD........
Os.Suv5d      .........................
Os.Suvh5a     .........................
Os.Suvh5b     .........................
At.Suvh6      .........................
Pa.Suvh7b     .........................
Pa.Suvh7c     .........................
Pa.Suvh7a     .........................
Os.Suvh7      .........................
Os.Suvh8      .........................
Os.Suvh11     .........................
Os.Suvh1      .........................
Cv.Suvh4      .........................
AtSuvh8       .........................
At.Suvh7      .........................
At.Suvh3      .........................
At.Suvh1      .........................
Pa.Suvh2c     .........................
Pa.Suvh2b     .........................
Pa.Suvh2a     .........................
Os.Suvh2      .........................
Os.Suvh9      .........................
AtSuvh2       .........................
At.Suvh9      .........................
Cv.Suvh2      Y........................
Mpu.Suvh4     .........................
Mpu.Suvr5     .........................
Cr.CLF        .........................
Pa.Suvh10a    .........................
Cp.Ashr2a     .........................
Cp.Ashr2b     .........................
Mp.Ashh2b     .........................
Pa.Suvr4      .........................
Cr.Ashh1      .........................
Cr.Suva       .........................
Os.Suvh10     .........................
Pa.Suvh10b    .........................
Pa.Atx6       .........................
Cr.Suvb       .........................
Vr.Suv        .........................
At.Suvh10     .........................
Ot.Ashh4      YE.IHFITNRDIEPGEELAYNY...
Os.Atxr3      YQ.IGIYTVRPIAEGEEITFDYNSV
Mpu.Atxr5     YA.VEMVSTCHIARGEEVCWDYN..
```

Contd...

```
Pp.Atxr5        AR.VILIAMRDILKGERLYYDYN..
Nm.Atxr5        AL.ALLIAIRDIRKGDFLYYDYN..
Pa.Atxr6a       SR.VLLVAIKDIAKGERLYYDYN..
Pa.Atxr6b       SR.VLLVAIKDIARGERLYYDYN..
At.Atxr6        AR.VLLVANRDISKGERLYYDYN..
Os.Atxr6        CR.VLLVANRDISKGERLYYDYN..
At.Atxr5        CR.VLLVATRDISKGERLYYDYN..
Os.Atxr5        SH.VLLVACRDIACGEKLYYDYN..
Cv.Ashr3b       .........................
Sm.Ashh1        T..LFCRAARDLKPGEELLSSYG..
At.Atxr1        Y..VIVHASRDIKTGEEISFAY...
Cr.Suvc         VPGVGLFARRDISVGEELTFPY...
Mpu.Suvh10      .........................
At.Ashr2        .........................
Sm.Ashr2        .........................
Nm.Ashr2        .........................
Cv.Ashr2b       .........................
Vc.Ashr2        .........................
Mr.Ashr2        .........................
Mpu.Ashr2       .........................
Ol.Atxr4        .........................
At.Ashr1        .........................
Mr.Ashr1b       .........................
Os.Atxr1        .........................
Cr.Ashr1        .........................
Cv.Ashr1        .........................
Cr.Ashr2        .........................
Ot.Atxr4a       .........................
Sm.Atxr4        .........................
At.Atxr4        .........................
Os.Atxr4        .........................
Cv.Ashr2a       .........................
Mpu.Ashr1       .........................
Ot.Atxr4b       .........................
Mr.Ashh1        .........................
Sm.Suvh2        .........................
Cv.Atxr         .........................
Pa.Atx1         .........................
Pa.Atx4         .........................
Mp.Atxr2        .........................
Nm.Atxr2        .........................
Os.Atxr2        .........................
Sm.atxr2        .........................
Ol.Atxrb        .........................
At.Atxr2        .........................
consensus>50    .........................
```

Additional file 3: Fig S1. Multiple sequence alignment of the 251 SET domain protein sequences from E(z), Ash, Trx and Su(var) of 16 Archeplastida species.
