# Supplementary material for: Phylogenetic relationship and domain organisation of SET domain proteins of Archaeplastida
Source: BMC Plant Biol. 2017 Dec 11;17:238. doi: 10.1186/s12870-017-1177-1 (PMC5725981; doi:10.1186/s12870-017-1177-1)
Supplement: Supplementary file 4 — Introduction of the domains in the Ash SET protein in cryptogam plant lineages. The black arrow indicates the introduction of the indicated domain in the specifically mentioned Archaeplastida species. (PDF 212 kb) [file 12870_2017_1177_MOESM4_ESM.pdf]

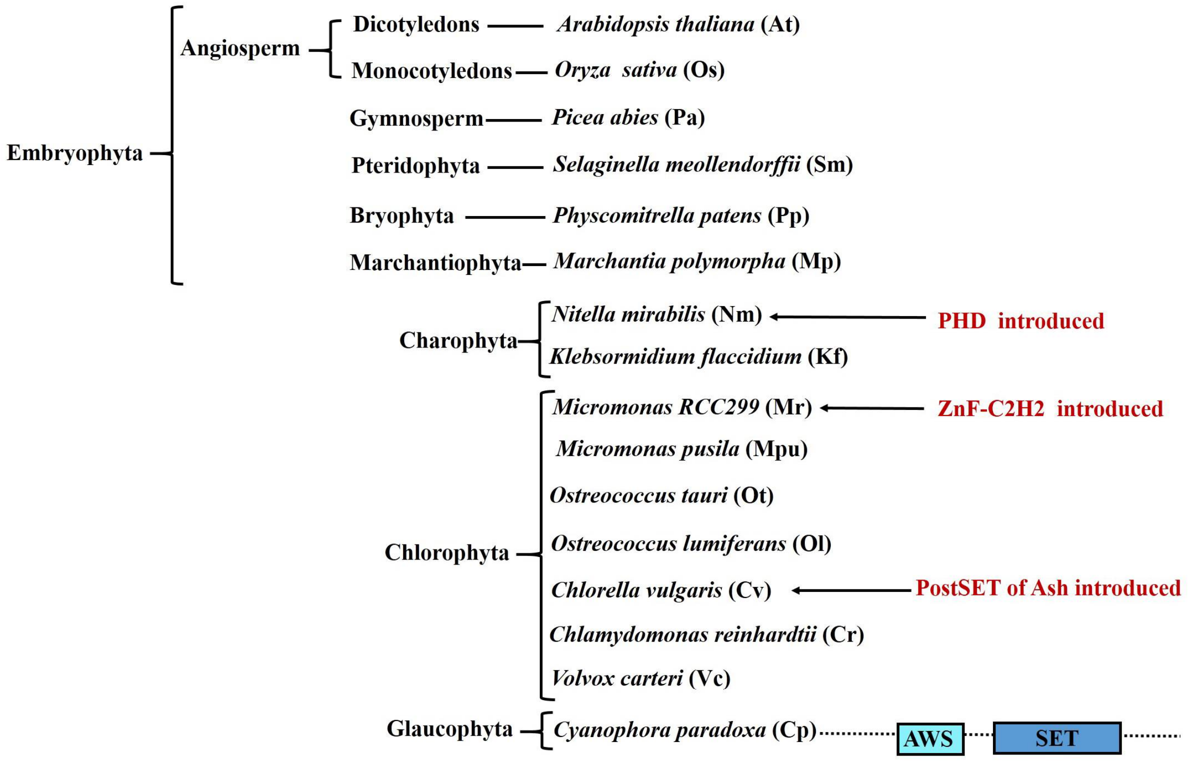

Additional file 4: Fig. S2. Introduction of the domains in the Ash SET protein in plant lineages. The black arrow indicates the introduction of the indicated domain in the specifically mentioned Archaeplastida species.
