# Supplementary material for: Phylogenetic relationship and domain organisation of SET domain proteins of Archaeplastida
Source: BMC Plant Biol. 2017 Dec 11;17:238. doi: 10.1186/s12870-017-1177-1 (PMC5725981; doi:10.1186/s12870-017-1177-1)
Supplement: Supplementary file 7 — Schematic diagrams showing the domain organization of Orphan proteins. (PDF 550 kb) [file 12870_2017_1177_MOESM7_ESM.pdf]

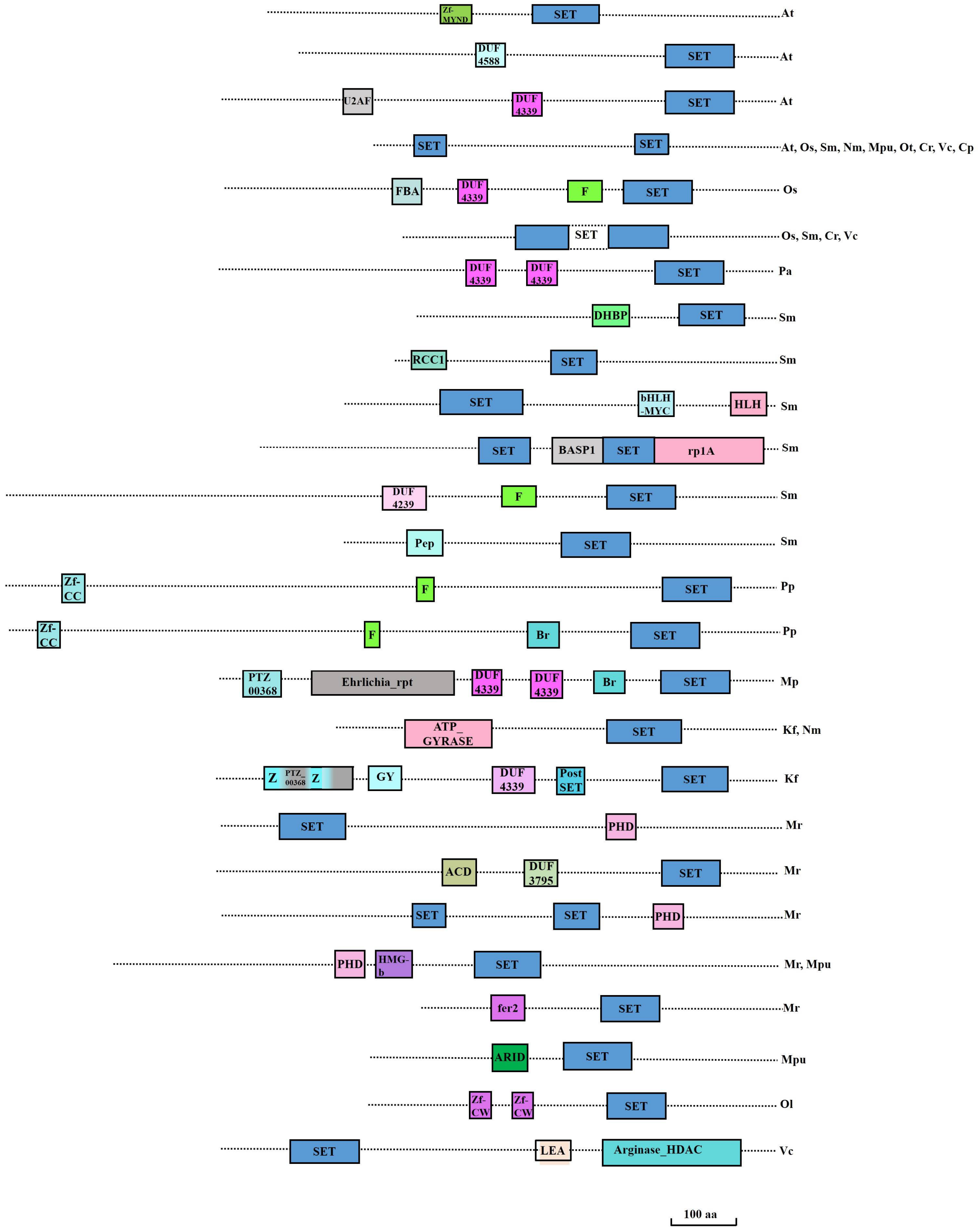

Additional file 7: Fig. S5. Schematic diagrams showing the domain organization of Orphan proteins. 26 different types of Orphan proteins are identified based on varied domain combinations. Species sharing the specific domain arrangement are indicated on right-hand side. Different protein domains are colored differently as indicated. DHBP: 3, 4-Dihydroxy-2-

butanone 4-phosphate; RCC1:Regulator of chromosome condensation; bHLH-MYC: Basic helix loop helix binding to MYC transcription factors; HLH: Helix loop helix; BASP1: Brain acid soluble protein 1; rp1A: Ribosomal protein L1; DUF4239:Domain of unknown function; F-Box: part of Skp1-cullin-F-box protein ligase; Pep: Peptidase; Ehrlichia rpt: tandem repeat found in immunodominant outer membrane protein of *Ehrlichia chaffeensis*; DUF4339: Domain of unknown function; ATP_GYRASE _4 superfamily: ATP-dependent carboxylate-amine ligase; PTZ_00368: Hypothetical protein; GY: GYF domain contains conserved Gly-Tyr-Phe; ACD: alpha-crystallin domain; DUF3795: Domain of unknown function; Fer2: 2Fe-2S iron-sulfur cluster binding domain; 20G_FeII_Oxy-2-Oxy glutarate: 2-oxoglutarate (2OG) and Fe(II)-dependent oxygenase superfamily; ARID: AT rich interaction domain; LEA: Late embryogenesis abundant domain; Arginase: Arginase-like amidino hydrolase family and histone/histone-like deacetylase; RecX-Receiver domain. Domains are not drawn to scale. Scale bars indicate 100 amino acids.
