# Supplementary material for: Phylogenetic relationship and domain organisation of SET domain proteins of Archaeplastida
Source: BMC Plant Biol. 2017 Dec 11;17:238. doi: 10.1186/s12870-017-1177-1 (PMC5725981; doi:10.1186/s12870-017-1177-1)
Supplement: Supplementary file 8 — Domain architecture of Class V- A) SETD and B) TPR family. (PDF 507 kb) [file 12870_2017_1177_MOESM8_ESM.pdf]

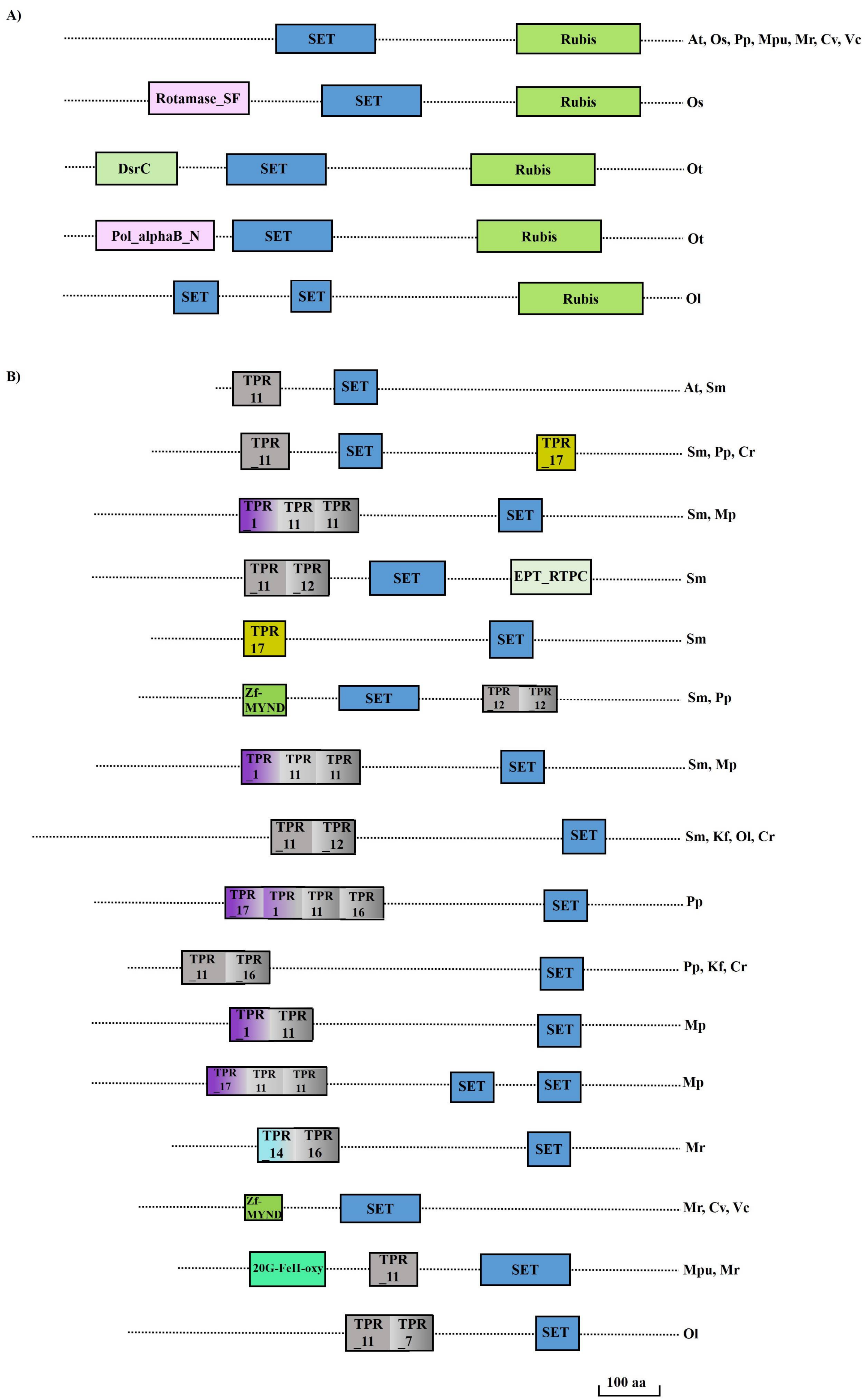
A)
SET
Rubis
At, Os, Pp, Mpu, Mr, Cv, Vc
Rotamase_SF
SET
Rubis
Os
DsrC
SET
Rubis
Ot
Pol_alphaB_N
SET
Rubis
Ot
SET
SET
Rubis
Ol
B)
TPR 11
SET
At, Sm
TPR _11
SET
TPR _17
Sm, Pp, Cr
TPR _1
TPR 11
TPR 11
SET
Sm, Mp
TPR _11
TPR _12
SET
EPT_RTPC
Sm
TPR 17
SET
Sm
Zf-MYND
SET
TPR 12
TPR 12
Sm, Pp
TPR _1
TPR 11
TPR 11
SET
Sm, Mp
TPR _11
TPR _12
SET
Sm, Kf, Ol, Cr
TPR 17
TPR 1
TPR 11
TPR 16
SET
Pp
TPR _11
TPR _16
SET
Pp, Kf, Cr
TPR _1
TPR 11
SET
Mp
TPR _17
TPR 11
TPR 11
SET
SET
Mp
TPR _14
TPR 16
SET
Mr
Zf-MYND
SET
Mr, Cv, Vc
20G-FeII-oxy
TPR _11
SET
Mpu, Mr
TPR _11
TPR _7
SET
Ol
100 aa

Additional file 8: Fig. S6. Domain architecture of Class V- A) SETD and B) TPR family. Schematic diagrams showing the domain organization of SETD and TPR family proteins. 5 different types of SETD and 16 types of TPR proteins are identified based on varied domain combinations. Species sharing the specific domain arrangement are indicated in right hand side. Different protein domains are colored differently as indicated. DsrC: Desulfovibrio vulgaris dissimilatory sulfite reductase; Pol_alphaB_N: DNA polymerase alpha subunit B N-terminal; EPT_RTPC: Enolpyruvate transferase (EPT) family and the RNA 3' phosphate cyclase family (RTPC); Ag: Agenet-related to TUDOR domain. Domains are not drawn to scale. Scale bars indicate 100 amino acids.
